# Acupuncture attenuates experimental autoimmune thyroiditis by modulating intestinal microbiota and palmitic acid metabolism

**DOI:** 10.3389/fimmu.2025.1541728

**Published:** 2025-04-28

**Authors:** Huimin Li, Fengjun Qi, Dan Li, Yun Fan, Jianmin Liu, Cui Li, Jun Chen, Xinyue Wu, Xiaojing Zhang, Fei Xu

**Affiliations:** College of Acupuncture and Orthopedics, Hubei University of Chinese Medicine, Wuhan, China

**Keywords:** autoimmune thyroiditis, acupuncture, intestinal microbiota, metabolomics, apoptosis

## Abstract

**Introduction:**

Autoimmune thyroiditis (AIT) is a common and chronic autoimmune disease. Recent evidence indicates that serum metabolites and dysbiosis of the intestinal microbiota are associated with AIT, with the underlying mechanism involving the apoptosis of thyroid follicular epithelial cells. Acupuncture, a traditional Chinese therapy, has demonstrated potential regulatory effects on various immune-related diseases. Clinical symptoms in AIT patients have shown improvement following acupuncture intervention. However, the mechanism underlying its therapeutic effects remain poorly understood.

**Methods:**

In this study, we investigated the mechanisms of acupuncture (Acu) treatment in rats with established experimental autoimmune thyroiditis (EAT) and evaluated the relationship between microbiota and serum metabolites after Acu treatment. After six weeks of acupuncture and Selenium yeast intervention (used as a positive control), enzyme-linked immunosorbent assay was used to employed to assess the expressions of serum thyroid function and inflammatory markers. Pathological changes in the thyroid gland were observed using hematoxylin-eosin staining and electron microscopy. Thyroid apoptosis was evaluated through TUNEL staining, immunohistochemistry and Western blot analysis. Additionally, changes in intestinal microbiota and serum metabolic profile were analyzed by 16S ribosomal RNA (16S rRNA) sequencing and LC-MS metabolomics, aiming to identify potential therapeutic targets for acupuncture intervention in AIT.

**Results:**

The results revealed that Acu could effectively improve thyroid dysfunction and histopathological changes in EAT rats. Following Acu treatment, the content of B-cell lymphoma-2 (Bcl-2) increased, while the levels of Bax and the proportion of cleaved caspase-3 in thyroid tissue decreased. This may be associated with the amelioration of intestinal microbiota dysbiosis and metabolic disorders in EAT rats. Acu mitigated EAT-induced metabolic disorders by regulating the metabolism of palmitoleic acid, and adjusted intestinal microbiota dysbiosis by increasing the abundance of Prevotella. Furthermore, the microbiota (Prevotella) and metabolites (Cyclohexanecarboxylic acid, Tetradecanedioic acid) may serve as co-targets for both Acu and Selenium yeast treatment in EAT.

**Discussion:**

Acu improves the apoptosis of thyroid follicular epithelial cells in rats in EAT model, and its mechanism may be related to intestinal microbiota and metabolism.

## Introduction

Autoimmune thyroiditis (AIT) is a chronic inflammatory condition characterized by the production of thyroid tissue-specific autoantibodies and lymphocyte infiltration, with high inflammatory load and cellular apoptosis. This immune response results in elevated levels of thyroid peroxidase and thyroglobulin antibodies, ultimately leading to thyroid tissue destruction and dysfunction (1, 2). The most prevalent form of AIT is Hashimoto’s thyroiditis (HT), which frequently manifests as endocrine regulatory disorders, followed by symptoms such as weight changes, anxiety, and palpitations, thereby diminishing quality of life (1). The prevalence of AIT varies by ethnicity, age, sex, and geographic region, with females being much more common than males (3, 4). The pathogenesis of AIT is complex, involving genetics, environmental factors, and aberrant activation of the immune system (2, 5). Among them, studies related to non-genetic factors have shown that the pathogenesis of AIT is closely related to intestinal microbiota and selenium deficiency (6).

With the recent advances in multiple omics techniques, gut microbiota and metabolomics analysis in the occurrence and development of diseases have received extensive attention (7). Numerous studies have demonstrated that the thyroid and gut share a common embryonic origin, and that AIT is linked to the diversity and abundance of certain gut microbiota, promoted by the negative effects on the immune system and the inflammatory regulation of the impaired microbiota, possibly through an imbalance in the gut microbiota that indirectly triggers apoptosis and inflammatory processes in healthy thyroid cells (8–10). Metabolomics examines the small molecule compounds in biological samples, which can reflect the metabolic changes resulting from intestinal dysbiosis in AIT patients, thereby providing crucial information for disease diagnosis and treatment (11).

In recent years, Acu has demonstrated significant efficacy and safety in the treatment of various autoimmune diseases (12–14). Acu exerts therapeutic effects by stimulating specific Acu points to modulate the body’s neuro-endocrine-immune network (15). Gut microbiota and metabolomics have been widely used for Acu intervention in autoimmune diseases (16, 17). However, the interaction between gut microbiota and serum metabolites, as well as their role in Acu treatment on AIT is still unclear.

Multiple studies have demonstrated that Zusanli (ST36) acupoint has been shown to play an anti-inflammatory and immunomodulatory role in the treatment of autoimmune diseases (18, 19). Stimulation of Sanyinjiao (SP6) and guanyuan (CV4) can delay inflammation by regulating metabolites or inhibiting the cleavage of apoptosis-related factors caspase (20, 21). We combined these acupoints to enhance the therapeutic effect synergistically and explored the underlying mechanism on autoimmune thyroiditis. In this study, the effect of Acu intervention was investigated by establishing an experimental autoimmune thyroiditis (EAT) rat model and using selenium yeast (Se-yeast) as a positive control (22). We hypothesized that acupuncture could play a role in alleviating thyroid inflammation and apoptosis by adjusting serum metabolite and intestinal microbial balance in EAT rats, which was verified by 16S rRNA sequencing of fecal samples and non-targeted metabolomics analysis of serum samples. We hope to clarify the mechanism of acupuncture treatment of EAT from the changes of metabolites and gut microbiota, and provide new clues for its treatment.

## Materials and methods

### EAT model establishment

Forty healthy female Sprague-Dawley rats with a weight of 170-200g were purchased from Experimental Animal Center of Hubei Province (SCXK(E) 2020-0018, Wuhan, China). In addition to the modeling and intervention phases, the rats were housed in the Laboratory Animal Center of Hubei University of Chinese Medicine with a temperature of (25 ± 0.5) °C, relative humidity (55 ± 5) %, 12 h/12 h circadian rhythm, and were allowed to drink and eat freely. The handling procedures of all animals during the experiment are in accordance with the regulations on the handling of experimental animals issued by the Animal Ethics Committee of Hubei University of Chinese Medicine (ethics approval number: HUCMS 00303194).

**Figure 1:**
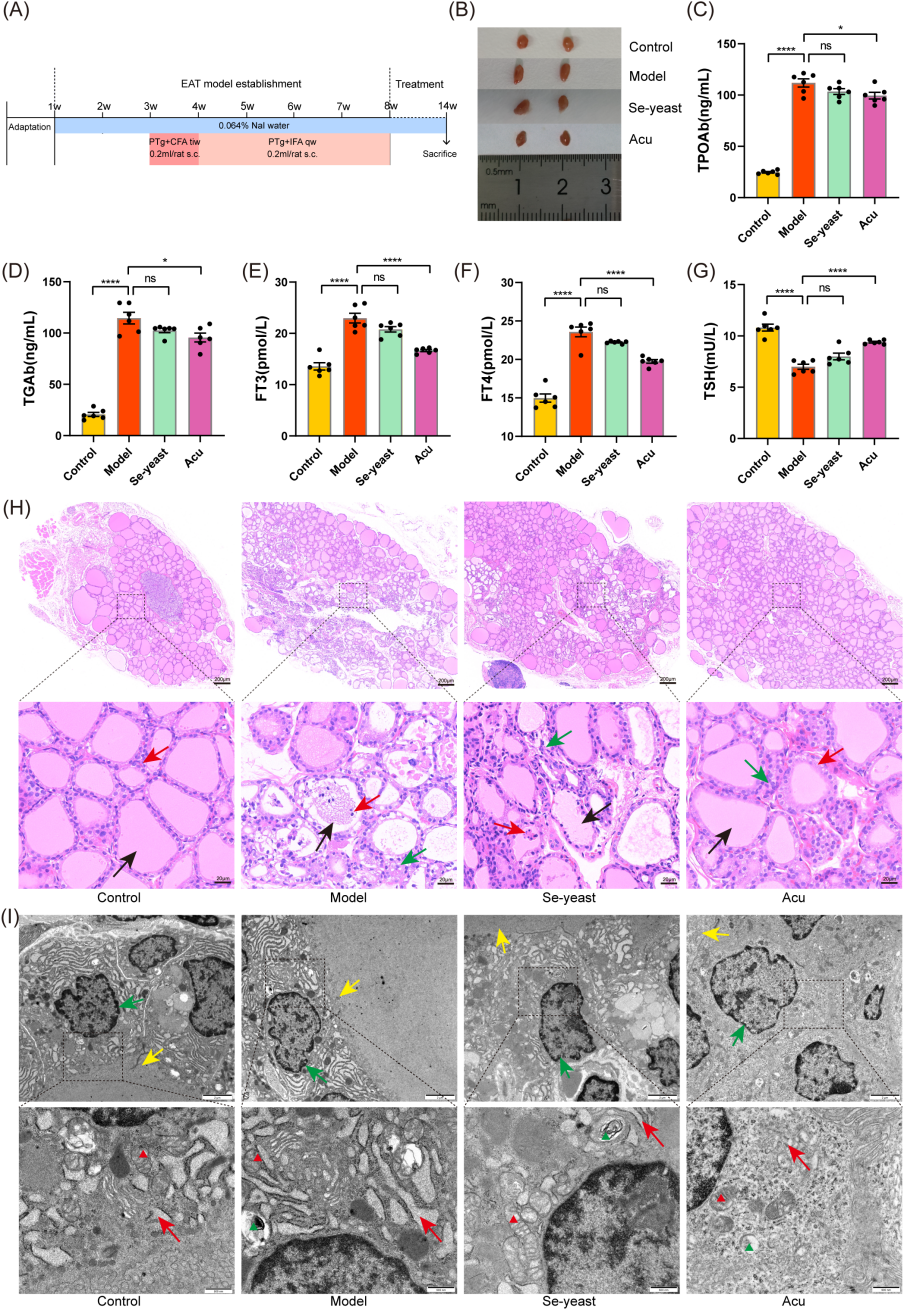
Acu attenuates thyroid dysfunction and structural abnormalities in EAT rats. **(A)** Experimental flow chart of autoimmune thyroiditis rat model preparation and Acu intervention. **(B)** Thyroid size comparison. **(C-G)** Serum test results of TPOAb, TGAb, FT3, FT4 and TSH (n=6). *P < 0.05, ****P < 0.0001, ns= P > 0.05 (ns, not statistically significant). **(H)** HE staining of thyroid tissue. The black arrow points to the thyroid follicles, the red arrow points to the follicular epithelial cells, and the green arrow points to the inflammatory cells. **(I)** Transmission electron microscope image of thyroid tissue. The green arrow points to the nuclear membrane, the yellow arrow points to the cilia, and the red arrow points to the endoplasmic reticulum. The red triangle marks the mitochondria and the green triangle marks the autophagy lysosomes.

After adaptive feeding for one week, the rats were randomly divided into four groups: control group, model group, selenium yeast (Se-yeast) group, and Acu (Acu) group (n=10 per group). Except for the control group, the EAT model was established using high-iodine water combined with subcutaneous injection of thyroglobulin emulsified with Complete Freund’s adjuvant (CFA) or Incomplete Freund’s adjuvant (IFA) (23). Sodium iodide was dissolved in distilled water to prepare 0.064% high-iodine water, and rats except the control group were allowed to drink high-iodine water freely from the second to the eighth week. A total of 0.1 mg of thyroglobulin (cat. no. T885815; Macklin) was dissolved in 100 μL of PBS and emulsified with 100 μL of CFA/IFA (cat. no. F5881/F5506; Sigma), and the final preparation of 0.05% thyroglobulin emulsifier is maintained. Primary immunization is performed at the fourth week, freshly prepared thyroglobulin emulsifier was injected subcutaneously into the bilateral foot pads of rats, 0.2mL per injection, twice a week. From the second to the eighth week, booster immunization was carried out, and thyroglobulin emulsified of IFA was injected into the dorsal subcutaneous part of the rat, 0.2mL per injection, once a week. Meanwhile, rats in the control group were allowed to drink distilled water freely during this period. Following the modeling process, tail vein blood was collected, and serum TPOAb level was detected by ELISA. After the successful modeling, the intervention was carried out over a total duration of six weeks (**Figure 1A**).

### Experiment design

The following intervention was implemented in the Acu group: the rats were anesthetized with 5% Isoflurane (cat. no. R510-22-10; RWD Life Science Co. Ltd, China) for 5 min by inhalation, and then maintained with 2% isoflurane in a mixture of 70% nitrous oxide and 30% oxygen for 15 min during acupuncture stimulation. rats were first lightly anesthetized before the Acu (needle size: 0.30mm × 25mm; Beijing Zhongyan Taihe) procedure to minimize pain and prevent unnecessary motor interference. After disinfecting the acupoints, sterile Acu needles were inserted into the fixed rats at a certain depth for 15min/rat/day. The selected Acu points included Zusanli (ST36), Sanyinjiao (SP6) and Guanyuan (CV4). The ST36 point is located beneath the knee joint of the rat and 3 mm below the fibular head in the muscle groove, and the SP6 is located 10 mm above the tip of the inner malleolus of the hind limb. Moreover, the CV4 is located 25 mm below the umbilicus. The remaining three groups of rats were fixed using the same method but did not receive Acu. In the Se-yeast group, rats were treated by gavage with a Selenium yeast solution containing Selenium 21μg/kg once a day (22). And the remaining three groups were given distilled water by gavage using the same method. This treatment lasted for 6 consecutive days followed by 1 day of rest, total 6 weeks of intervention. Except for the control group, all rats had free access to 0.064% iodine water throughout the intervention period.

### 16S rRNA gene sequencing analysis

After the intervention, the feces of the rats were aseptically collected and placed in a 5mL EP tube. The samples were then immediately frozen using liquid nitrogen and stored at -80°C for subsequent analysis of intestinal bacterial microbial diversity.

Genomic DNA was extracted and analyzed using 1% agarose gel electrophoresis. Polymerase chain reaction (PCR) was conducted with TransStart Fastpfu DNA Polymerase (cat.no. AP221-02; TransGen). All samples were processed under controlled experimental conditions, 3 replicates per sample, PCR products of the same sample were mixed and detected by 2% agarose gel electrophoresis, and PCR products were recovered by cutting gel using the AxyPrep DNA Gel Recovery Kit (Axygen, Silicon Valley, United States). PCR products were fluorescently quantified using the QuantiFluor-ST™ Blue Fluorescence Quantification System (Promega, Wisconsin, United States), and then mixed in proportions according to the amount of sequencing required for each sample. Qualified libraries were constructed using the TruSeq™ DNA Sample Prep Kit and sequenced on the machine. The 16S rRNA sequencing work was provided by Majorbio (Shanghai, China).

### LC–MS untargeted metabolomics

In order to avoid food interference with the content of serum metabolites, the rats were fasted for 12h before euthanasia, but water was not restricted. Rats were anesthetized with 5% isoflurane inhalation for 5 minutes, followed by maintenance of anesthesia with 2% isoflurane in a mixture of 70% nitrous oxide and 30% oxygen. After confirming deep anesthesia in the rats, blood was collected from the abdominal aorta, followed by decapitation for euthanasia. To test for serum metabolites, the serum was obtained by centrifugation at 2000rpm at 4°C for 10min after abdominal aorta blood resting for 30min, and subsequently stored at -80°C.

After thawing, 400 µL of a cold methanol solution (methanol: water = 4:1) was added to the appropriate amount of sample. At low temperature, the samples were disrupted using a high-throughput tissue breaker, and then ultrasonically extracted three times on ice, with each extraction lasting 10 min after vortex mixing. The samples were placed at -20°C for 30 min, centrifuged at 4°C and 13,000 g for 15 min, and then the supernatant was analyzed by ultra-performance liquid chromatography tandem time-of-flight mass spectrometry UPLC-Triple TOF system (AB SCIEX). The identified metabolites were compared to the Kyoto Encyclopedia of Genes and Genomes (KEGG) compound database (https://www.kegg.jp/kegg/compound/) and the Human Metabolome Database (HMDB, https://hmdb.ca/) to obtain the metabolite classification profile, which was subsequently statistically graphed. Univariate statistical analysis (t-test) was combined with multivariate statistical analysis (OPLS-DA/PLS-DA) and fold change value (FC) to screen for differential metabolites, and the screening conditions were P<0.05 and VIP>1. LC–MS untargeted metabolite analysis was provided by Majorbio (Shanghai, China).

### Serum biochemistry analysis

Serum levels of FT3, FT4, TSH, TgAb, TPOAb, and tumor necrosis factor-α (TNF-α) and Interleukin-10 (IL-10) were detected by ELISA with bispecific antibodies. The serum sample was 100 μL, and the absorbance (OD value) was measured with a microplate reader at 450 nm wavelength to calculate the sample concentration.

### Transmission electron microscopy

After euthanasia, one unilateral thyroid gland was excised and immediately immersed in a 2.5% glutaraldehyde solution in 0.1M phosphate buffer saline (PBS, pH 7.4) for 4 h. Subsequent to post-fixation, dehydration, permeation, and embedding were carried out sequentially, the samples were sliced with an ultramicrotome (cat.no. Leica-UC7; Leica). After uranium-lead double staining (2% uranium acetate saturated aqueous solution and lead citrate, staining for 15 min each), the sections were observed under transmission electron microscope (cat.no. JEM-1400; Japan Electron). Images were acquired to analyze the ultrastructure.

### Histology examination

Unilateral thyroids from three rats in each group were excised and immediately fixed in 4% paraformaldehyde (cat.no. BL539A; Biosharp) for at least 24 h. The dehydration, transparency and wax impregnation are completed in the biological tissue dehydrator (cat.no. JT-12J; Wuhan Junjie), so that the tissue blocks soaked in wax are wrapped in paraffin blocks. The tissue structure was examined under a light microscope (cat.no. Flexacam-C1; Leica) after sectioning and HE staining.

### Immunohistochemistry

The thyroid wax sections were deparaffinized by an electric ceramic oven for antigen retrieval used 0.01M Tris-EDTA repair solution (pH 9.0). 3% hydrogen peroxide blocked endogenous peroxidase, normal goat serum (cat. no. AR1009; Boster) was blocked at room temperature for 30 min, and then specific antibody rabbit polyanti-Cleaved-caspase3 (1:200, cat. no. AF7022; Affnity) was added, and incubated at 4°C for 15 h. After PBS rinsing, HRP-labeled goat anti-rabbit/mouse secondary antibody was added dropwise and incubated at 37°C for 30 min. Drying sections were acquired under the microscope after DAB color development, hematoxylin counterstaining, PBS sufficient blue-back dehydration and mounting.

### TUNEL assay

Following the instructions for the TUNEL Apoptosis Assay (cat. no. 11684817910; Roche Applied Science), proteinase K working solution (10 μg/mL) was added dropwise to dewaxed sections, reacted at 25°C for 30 min, and washed with PBS 3 times for 5 min each time. In the treatment group, 50 μL of TUNEL reaction mixture (50 μL of enzyme solution + 450 μL of fluorescein labeling solution) was added dropwise to the samples, while only 50 μL of fluorescein labeling solution was added to the negative control group, and incubated at 37°C for 60 min in the dark. 50μL -100μL of DAB chromogenic solution dropwise was added. Harris hematoxylin was counterstained for 1 min, washed with 1% hydrochloric acid alcohol after water differentiation, and then washed back to blue with PBS. After dehydrated transparent and air-dried neutral gum mounting, dried sections can be viewed under a microscope.

### Western blot analysis

Three samples were selected from each group and extracted with RIPA lysis buffer (cat. no. P0013D; Beyotime) with a protease inhibitor cocktail (cat. no. 4693132001; MERCK) and phosphatase inhibitor cocktail (cat. no. P1046, Beyotime). The total protein solution was heated at 98°C for 10 min. And then denatured and gel filling, electrophoresis and transfer were carried out according to the molecular weight of the protein. The PDVF membrane was sealed with 5% skimmed milk powder for 1 h, diluted with primary antibody diluent and incubated overnight at 4°C according to the following ratio: Bax (1:1000, cat. no. A12009; Abclonal), Bcl-2 (1:1000, cat. no. A11025; Abclonal), caspase-3 (1:1000, cat. no. A0214; Abclonal), cleaved-caspase-3 (1:1000, cat. no. AF7022; Affinity), internal reference protein β-Tubulin (1:1000, cat. no. ab179513; abcam). The secondary antibody HRP-Goat anti Rabbit (1:3000, cat. no. JAC-115-035-003; JacKson) was added and diluted in 5% skimmed milk powder and incubated for 1 h, washed with TBS with Tween-20 (TBST) for 5 times for 5 min each time. Luminescence detection was performed, the exposure conditions were adjusted according to different light intensities, the development and fixing. The optical density value of the target band was analyzed by the ImageJ software processing system after scanning.

### Statistical analysis

Results were analyzed using GraphPad Prism 8 software (San Diego, United States) and expressed as mean ± standard deviations. The Shapiro-Wilk test was used to evaluate the normality of the data, and if the normal distribution and variance homogeneity were satisfied, the data were analyzed by one-way analysis of variance (ANOVA). The non-normal distribution of data between the two groups was compared using the Mann-Whitney U test and the nonparametric Kruskal-Wallis test of three or more groups. P<0.05 was considered statistically significant. 16S rRNA sequencing and untargeted metabolomics analysis were performed using the online platform of Majorbio Cloud Platform (https://www.majorbio.com/).

## Results

### Changes in thyroid function and tissue structure of EAT rats

Compared to the control group, the thyroid tissue volume in the model group increased, and the volume decreased after selenium yeast and Acu intervention (**Figure 1B**). The serum levels of TPOAb, TGAb, FT3 and FT4 increased significantly (P<0.0001, **Figure 1C–F**), and the TSH decreased in the model group (P<0.0001, **Figure 1G**). The indicators showed an improvement trend after selenium yeast intervention. After Acu intervention, TPOAb (P=0.0360), TGAb (P=0.0109), FT3 (P<0.0001) and FT4 (P<0.0001) decreased and TSH (P<0.0001) increased.

Furthermore, HE staining was used to demonstrate the impact of Acu on the histopathological changes of EAT rats. Under light microscopy, it was observed that the thyroid follicular cavity was intact, the size was moderate, the glial staining in the cavity was uniform, and the morphology of thyroid follicular epithelial cells was regular in the control group. In contrast, the thyroid follicular cavity was damaged of the model group, the glial staining was pale and uneven, or even vacuole-like without staining, and the follicular epithelial cells were swollen, damaged, sloughed off and necrotic in the cavity, and a large number of lymphocytes could be infiltrated in the stroma. However, the Acu and selenium yeast treatment preserved the integrity of the follicular epithelial cells and reduced lymphocytic infiltration in the EAT rats (**Figure 1H**).

Transmission electron microscopy revealed that the nuclear membrane of thyroid follicular epithelial cells was intact, normal in size and morphology, and the endoplasmic reticulum in the control group, mitochondria and other organelles were in complete morphology and normal in structure. In the model group, the nuclear membrane was ruptured, the endoplasmic reticulum and mitochondria were swollen and destroyed, the cell membrane boundary was blurred, and the number of microvilli was reduced. Notably, Acu and selenium yeast treatment alleviate this phenomenon (**Figure 1I**). Altogether, these findings suggest that Acu therapy may enhance thyroid function as well as alleviate tissue structure destruction in the EAT rats.

### Effect of Acu on thyroid cell apoptosis in EAT rats

**Figure 2:**
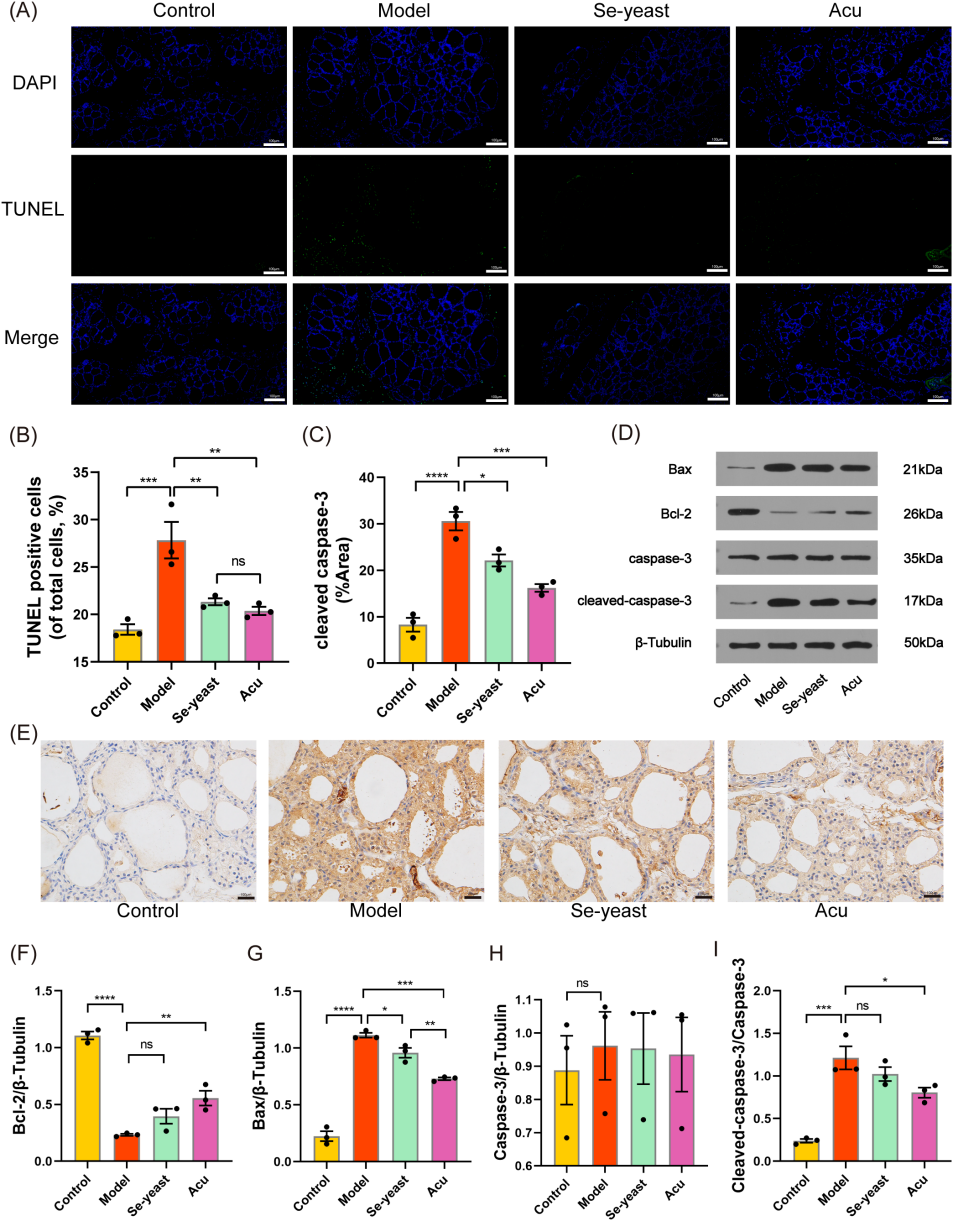
Acu attenuates thyroid apoptosis in EAT rats. **(A, B)** TUNEL staining (green) shows the level of apoptosis in thyroid tissue (n=3). **(C, E)** Cleaved caspase-3 protein levels in thyroid tissues shown by immunohistochemical staining (n=3). **(D, F-I)** Representative western blot bands and quantitative analysis of apoptosis marker in different groups (n=3). *P<0.05, **P<0.01, ***P<0.001, ****P<0.0001, ns=P>0.05 (ns, not statistically significant).

TUNEL staining was used to detect thyroid tissue apoptosis, IHC-P was used to detect the positive expression rate of cleaved caspase-3, and WB was used to detect the relative expression levels of Bax, Bcl-2, caspase-3 and cleaved caspase-3 proteins. Compared with the control group, the rate of TUNEL apoptosis-positive cells in the model group was significantly increased (P=0.0009, **Figures 2A, B**). Following treatment with selenium yeast and Acu, the rate of apoptosis-positive cells decreased (P=0.0095, P=0.0042). The positive expression rate of cleaved caspase-3 in the model group was significantly increased (P<0.0001), and the positive expression rate of cleaved caspase-3 decreased in Acu group and Se-yeast group (P=0.0206, P=0.0144, **Figures 2C, E**). Compared with the control group, the Bcl-2 content in the model group decreased (P<0.0001), the Bax content and the ratio of cleaved caspase-3 to caspase-3 increased (P<0.0001, P=0.0002, **Figure 2D**). These results were reversed after following Acu and selenium yeast treatment (**Figures 2F–I**).

### Effect of Acu on serum metabolic profile in rats

**Figure 3:**
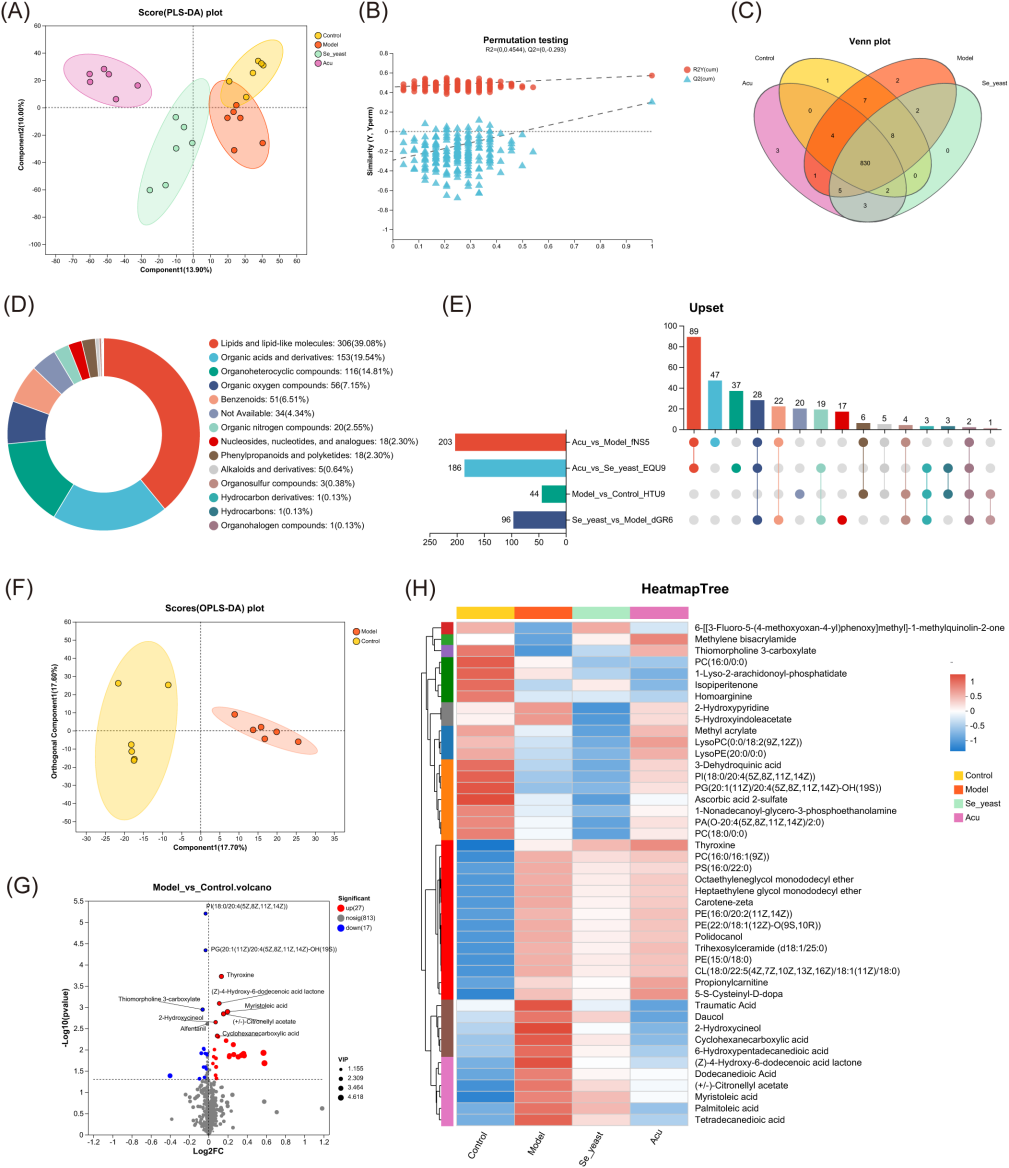
Serum metabolomics of the control, model, Se-yeast and Acu groups. **(A)** PLS-DA scores plots of four groups. The abscissa and ordinate represent the degree of interpretation of Component1 and Component2 respectively. **(B)** Permutation testing. The abscissa represents the displacement retention of the displacement test, the ordinate represents the values of the R2 and Q2 displacement tests. **(C)** Venn distribution of metabolites. **(D)** Metabolite proportion super class chart of HMDB. **(E)** Upset chart of comparison of metabolites data across. **(F)** OPLS-DA scores plots of control with model. **(G)** Volcano plot between control and model groups. The red dots represent significantly up-regulated, the blue dots represent significantly down-regulated, and the gray dots are the non-significantly differential metabolites. **(H)** Heatmap tree displaying 44 differential metabolites used for hierarchical clustering. Blue color indicates down regulation whereas red color shows up regulation. N=6 in each group.

The study on LC-MS non-targeted serum metabolomics revealed that Acu effectively reversed serum metabolomic disorders in EAT rats. The Partial Least Squares Discriminant Analysis (PLS-DA) diagram showed that the metabolites of four groups relatively separated in the positive and negative ion mixed mode, indicating that the serum metabolism disorder appeared in the EAT rats, that Acu and selenium yeast had a significant effect on serum metabolite profiles (**Figure 3A**). In the PLS-DA displacement test chart, with the decrease of displacement retention, R2 (0.571, P<0.05) and Q2 (0.299, P<0.05) decreased, and the regression line showed an upward trend, indicating that the replacement test passed (**Figure 3B**).

Subsequently, a Venn diagram was employed to identify a total of 830 metabolites across the four groups (**Figure 3C**). A metabolite set of 830 metabolites was created and the compounds were classified in the Human Metabolism Database (HMDB, https://hmdb.ca/). According to the number of metabolites, the names of the top 20 metabolites in the **Figure 3D** were obtained. After the four groups of metabolites in pairs, the Upset plot showed that there were 44 differential metabolites in the control group and the model group, 96 in the model group and selenium yeast group, and 203 in the model group and Acu group, indicating that Acu had a significant effect on serum metabolism in EAT rats (**Figure 3E**). The OPLS-DA plot showed that the metabolites of the control and model group were clustered on both sides, and the differences were significantly separated and comparable (**Figure 3F**). There were 44 differential metabolites in EAT rats, of which 27 were upregulated and 17 were downregulated (**Figure 3G**). A metabolite cluster analysis was conducted on these 44 differential metabolites, and the resulting heat map revealed that 4 clusters of serum metabolites were upregulated while 6 clusters were downregulated, which were reversed after Acu intervention. This evidence supports the conclusion that Acu could effectively alleviate serum metabolic disorders in EAT rats (**Figure 3H**).

**Figure 4:**
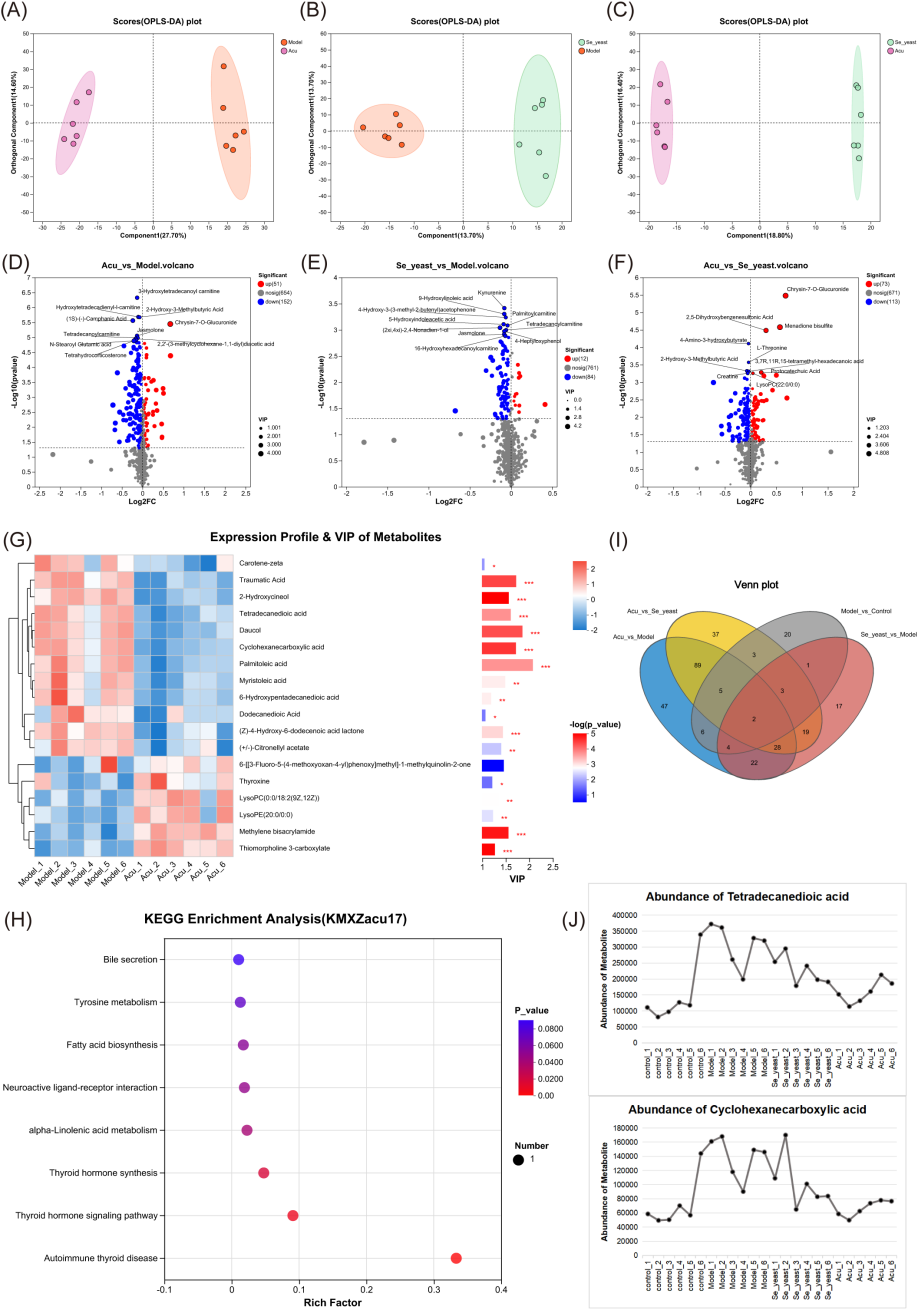
Metabolic set analysis. **(A)** OPLS-DA scores plots of Acu with model. **(B)** OPLS-DA scores plots of se-yeast with model. **(C)** OPLS-DA scores plots of Se-yeast with Acu. **(D)** Volcano plot showing the differential abundance of metabolites between Acu and model groups. **(E)** Volcano plot showing the differential abundance of metabolites between Se-yeast and model groups. **(F)** Volcano plot showing the differential abundance of metabolites between Se-yeast and Acu groups. **(G)** The VIP score used to rank the discriminate contribution of metabolites. The red represents the high expression of the metabolite, and the blue represents the low expression. *P<0.05, **P<0.01, ***P<0.001. **(H)** Dot plot of KEGG pathways Enrichment analysis influenced by Acu. **(I)** Venn distribution of metabolites of analysis of pairwise comparisons. **(J)** Abundance of metabolites. Sample size n=6 in each group.

The 44 differential metabolites identified from both the control group and model groups underwent further analysis. Pairwise comparison of OPLS-DA plots among the model group, Se-yeast group, and Acu group are presented in **Figures 4A–C**, indicating that both selenium yeast and Acu have a significant effect on EAT rats. The volcano graphs of differential metabolites compared between the groups are shown in **Figures 4D–F**. The differences between the Acu and model group were analyzed among the 44 differential metabolites, and 18 differential metabolites were obtained, which were shown in the VIP plot (**Figure 4G**). When applying a VIP threshold of <1, 12 metabolites were found to be down-regulated, including palmitoleic acid, daucol, traumatic Acid, cyclohexanecarboxylic acid, tetradecanedioic acid, 2-Hydroxycineol, myristoleic acid, (Z)-4-Hydroxy-6-dodecenoic acid lactone, (+/-)-Citronellyl acetate, dodecanedioic acid, 6-Hydroxypentadecanedioic acid and carotene-zeta. Conversely, 5 metabolites were up-regulated, including methylene bisacrylamide, lysoPC(0:0/18:2(9Z,12Z)), thyroxine, thiomorpholine 3-carboxylate and ysoPE(20:0/0:0). KEGG enrichment analysis showed that the 8 metabolic pathways were greatly affected, including bile secretion, tyrosine metabolism, fatty acid biosynthesis, and neuroactive ligand-receptor interaction, alpha-Linolenic acid metabolism, Thyroid hormone synthesis, thyroid hormone signaling pathway and autoimmune thyroid disease (**Figure 4H**). Collectively, these results suggest that Acu regulates serum metabolism and subsequent metabolite synthesis, particularly palmitoleic acid, which may influence autoimmune thyroiditis through circulation.

The Venn diagrams illustrating the four pairwise comparisons showed that there were two metabolites that were co-intersected, namely cyclohexanecarboxylic acid and tetradecanedioic acid (**Figure 4I**). The expression distribution of both groups increased in the model group and decreased after selenium yeast and Acu, indicating that they may be common targets for Acu and selenium yeast in autoimmune thyroiditis (**Figure 4J**).

### Combined analysis of serum metabolites and thyroid function and inflammatory factors by Acu intervention

**Figure 5:**
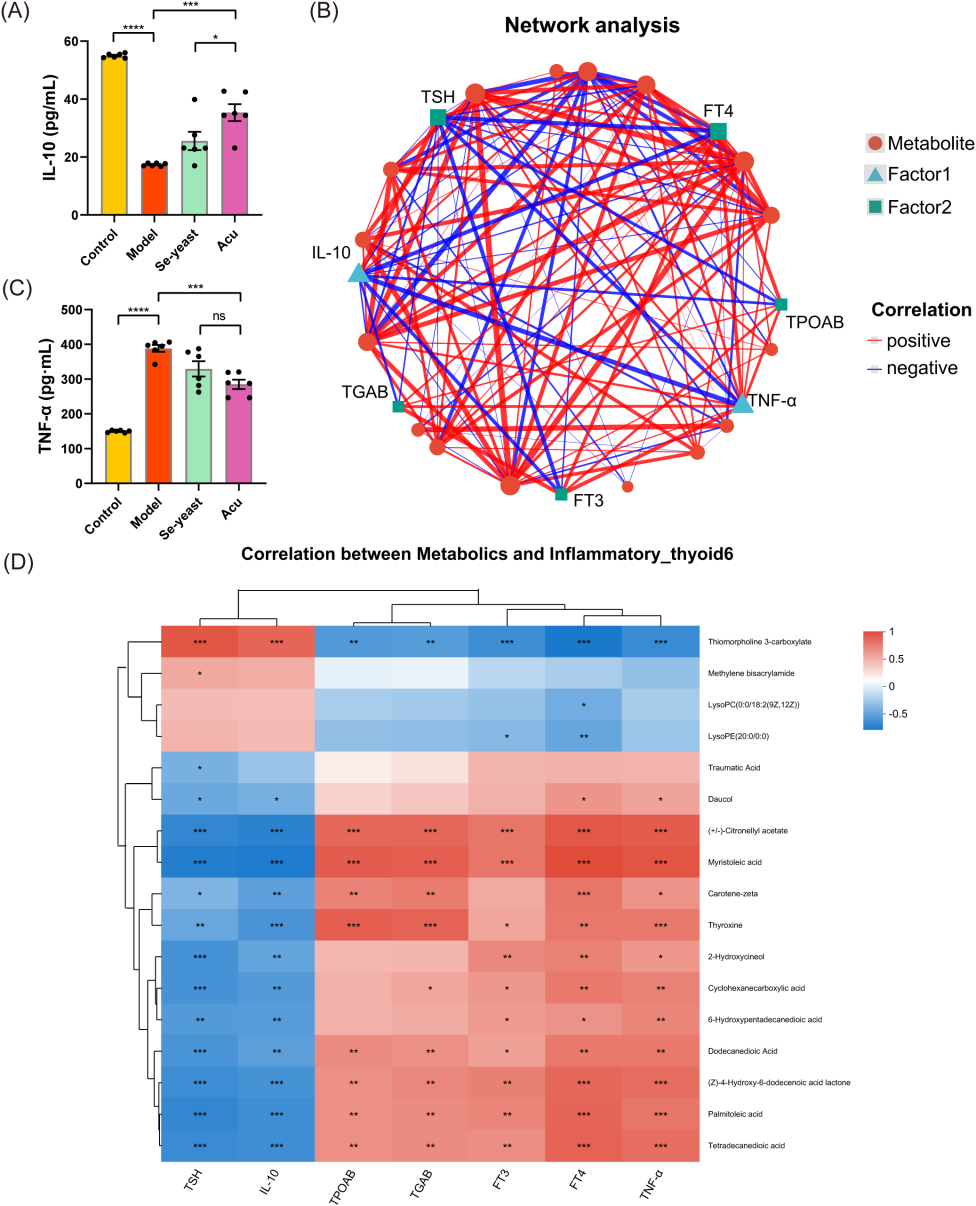
Combined analysis of serum metabolites, thyroid function and inflammatory factors under Acu intervention. **(A, B)** Serum test results of TNF-α and IL-10 (n=6). *P<0.05, **P<0.01, ***P<0.001, ****P<0.0001, ns=P>0.05 (ns, not statistically significant). Network Analysis. The size of the node in the figure represents the degree size of the node. The thickness of the line indicates the magnitude of the correlation coefficient. **(D)** Heatmap of correlation of dominant serum metabolites and the correlation between key metabolites, thyroid function and inflammatory factors under Acu intervention. *P<0.05, **P<0.01, ***P<0.001. Sample size n=6 in each group.

To investigate the effects of thyroid function and inflammatory factor indexes on metabolites, we examined 17 differential metabolites obtained by Acu intervention in the serum metabolome analysis of EAT rats were combined with thyroid function indexes, inflammatory factors TNF-α and IL-10. Serum TNF-α increased (P<0.0001) and IL-10 decreased (P<0.0001) in EAT rats, which were reversed by Acu and selenium yeast intervention (**Figures 5A, B**). The multi-factor correlation network diagram visually shows the closeness of the interconnection (**Figure 5C**). In the correlation heat map, the different colors represent the magnitude of the correlation coefficient between the attributes. Notably, thiomorpholine 3-carboxylate, (+/-)-Citronellyl acetate, myristoleic acid, thyroxine, dodecanedioic acid, (Z)-4-Hydroxy-6-dodecenoic acid lactone, palmitoleic Acid and tetradecanedioic acid showed strong correlation with thyroid function, TNF-α and IL-10 at the same time, further refining the target range of Acu in EAT rats (**Figure 5D**).

### Effect of Acu on intestinal microbiota in EAT rats

**Figure 6:**
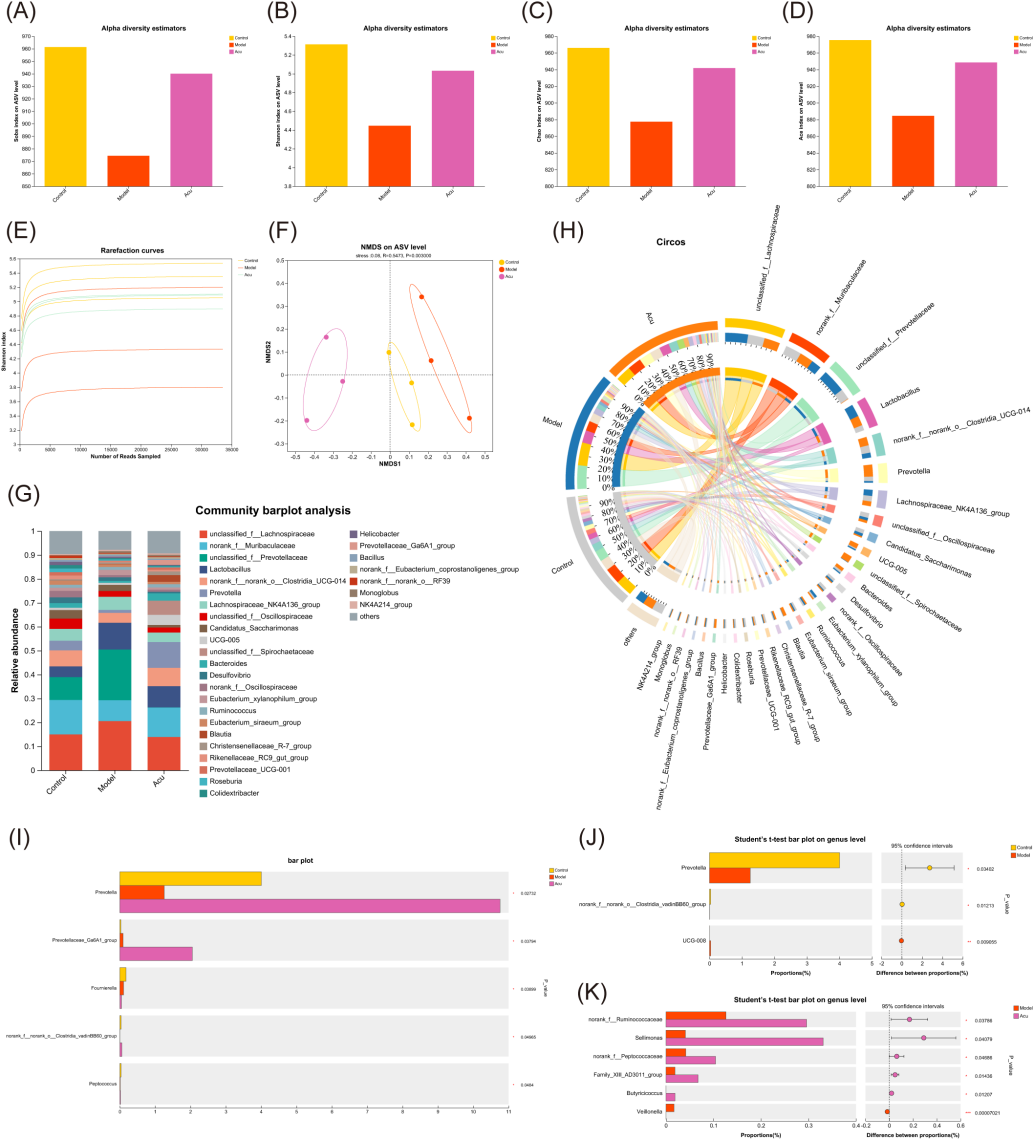
Gut microbial alterations in EAT rats treated with Acu. **(A-D)** Sobs, Shannon, Chao and Ace index chart of Alpha diversity statistics. **(E)** Rarefaction curves of Shannon index. **(F)** Non-metric multidimensional scaling analysis on ASV level. **(G)** Bar plot of community abundance analysis on genus level. **(H)** Circos plot of community abundance analysis on genus level. **(I)** Multi-group comparative analysis bar plot on genus level. **(J)** Student’s t-test bar plot on genus level of Model with Control. **(K)** Student’s t-test bar plot on genus level of Acu with Model. *P<0.05, ***P<0.001.

To investigate the regulatory effect of Acu on the intestinal microbiota of EAT rats, the changes of intestinal microbiota in feces were analyzed by 16S rRNA gene sequencing. The alpha diversity of Sobs, Shannon, Chao, and Ace indices at amplicon sequence variant (ASV) levels showed a decrease in the model group. In contrast, the Acu group was significantly reversed, indicating that it protected the gut microbiota diversity in EAT rats (**Figures 6A–D**). The end of the trend of the dilution curve tended to be flat, indicating that the amount of sequencing data of the samples was reasonable (**Figure 6E**). Non-metric multidimensional scaling analysis (NMDS) was used to analyze the ASV level between groups (R=0.5473, P=0.003<0.01). The distinct separation of samples from each group, indicates that the intestinal microbiota of EAT rats became disordered and altered following Acu intervention (**Figure 6F**).

Subsequently, the community structure of each group was analyzed at the genus level. The dominant strains were *unclassified_f:Lachnospiraceae*, *norank_f:Muribaculaceae*, *unclassified_f:Prevotellaceae* and *Lactobacillus*. The relative levels of *unclassified_f:Lachnospiraceae*, *unclassified_f:Prevotellaceae*, and *Lactobacillus* increased and *norank_f:Muribaculaceae* decreased in the model group, while decreased after Acu intervention (**Figures 6G, H**). The Kruskal-Wallis rank-sum test was used to compare and analyze the community abundance data at the genus level according to the community abundance data, and the species information with significant differences between the groups was obtained. The results showed that there were 5 significantly different species in the three groups (**Figure 6I**), and the contents of *Prevotella*, *norank_f:norank_o:Clostridia_vadinBB60_group* and *Peptococcus* in the model group decreased, while increased after Acu treatment. The results of pairwise comparison at the genus level showed that *Prevotella* changed significantly between the control group and the model group, suggesting that it may be the target of Acu intervention in the intestinal microbiota of EAT rats (**Figures 6J, K**).

**Figure 7:**
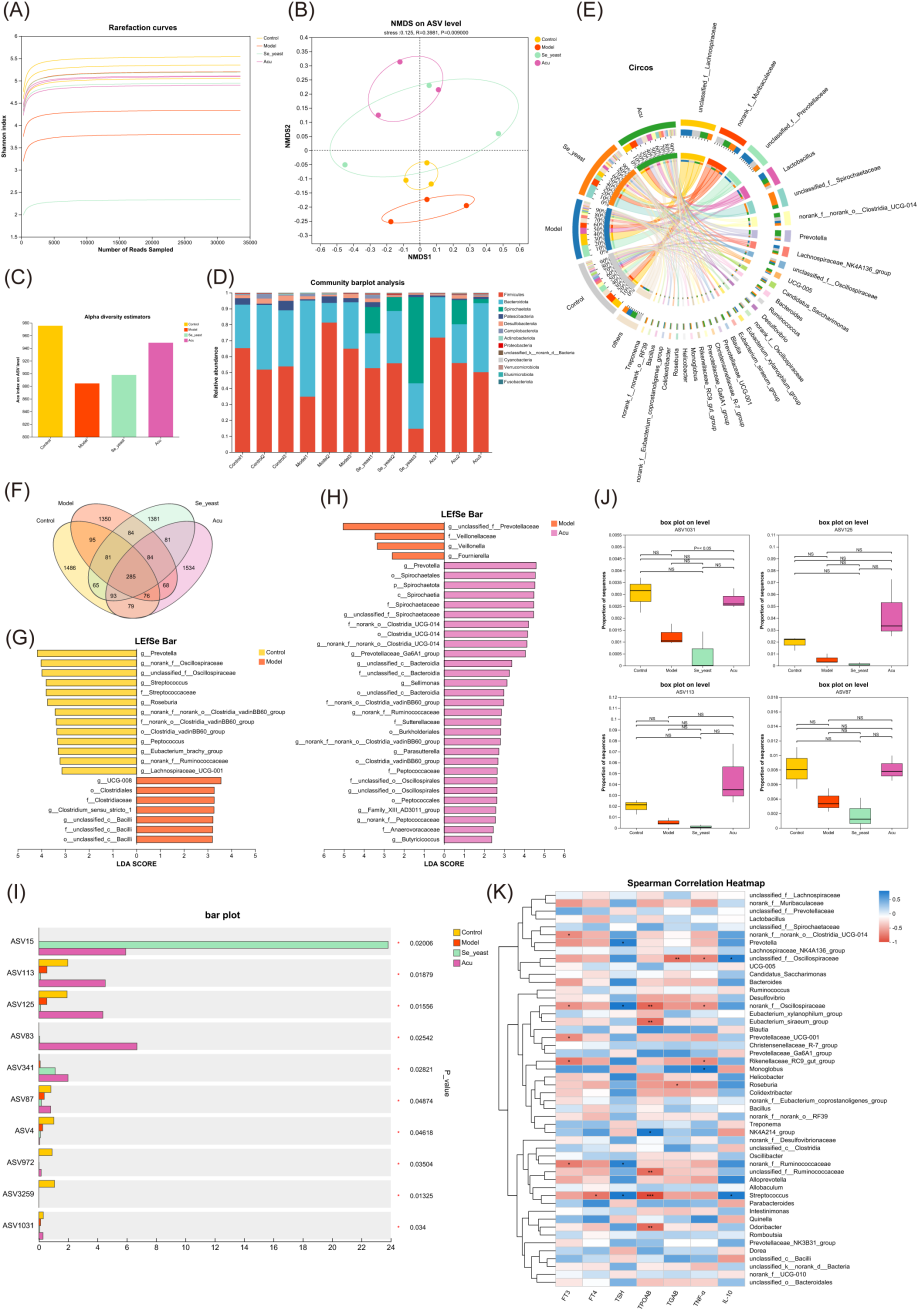
Gut microbial alterations in EAT rats treated with selenium yeast and Acu. **(A)** Rarefaction curves of Shannon index. **(B)** Non-metric multidimensional scaling analysis on ASV level. **(C)** Ace index chart of Alpha diversity statistics. **(D)** Bar plot of community abundance analysis on Phylum level. **(E)** Circos plot of community abundance analysis on genus level. **(F)** Venn diagram on ASV level. **(G)** LDA discriminant histogram of Model with Control. **(H)** LDA discriminant histogram of Acu with Model. **(I)** Multi-group comparative analysis bar plot on ASV level. **(J)** Box plot of ASV1031, ASV125, ASV113 and ASSV87. **(K)** Spearman correlation Heatmap between thyroid function, inflammatory factors and gut microbiota. *P<0.05, **P<0.01.

Then the Se-yeast group was added for analysis to find the common target of Acu and selenium yeast in regulating the intestinal microbiota of EAT rats. The end of the alpha diversity tends to be flat, so the amount of sequencing data in the sample is reasonable (**Figure 7A**). NMDS analysis of ASV levels indicated that the microbiota was changed after Acu and selenium yeast intervention (R=0.3981, P=0.009, **Figure 7B**). Acu and Se-yeast significantly increased the ACE index and protected the gut microbiota diversity in EAT rats (**Figure 7C**). Subsequently, the community structure of each group was analyzed at different taxonomic levels. At the phylum level, *Firmicutes*, *Bacteroidota* and *Spirochaetota* were the dominant strains, but there were little differences among the groups. Compared with the control group, the relative content of *Desulfobacterota* in the model group decreased, while increased after Acu intervention (**Figure 7D**). At the genus level, *unclassified_f:Lachnospiraceae*, *norank_f:Muribaculaceae*, *unclassified_f:Prevotellaceae* and *Lactobacillus* were the dominant strains, and the relative content of *unclassified_f:Lachnospiraceae* in the model group was higher than that in the control group, and decreased after Acu and selenium yeast intervention (**Figure 7E**).

There was a total of 285 species in the four groups, and the ASV number of the model group decreased, the Acu and Se-yeast group increased, indicating that Acu and selenium yeast intervention played an important role in maintaining the abundance of intestinal microbiota in EAT rats (**Figure 7F**). Linear discriminant analysis of effect size (LefSe) was used to compare the changes of fecal microbes in the Control, Model, Se-yeast and Acu groups in pairwise to find the biomarkers of intestinal microbiota in each group (LDA>2). A total of 20 species in the control and model groups were found to have changed abundance (**Figure 6G**). The Acu intervention had a significant effect on the abundance of 33 species (**Figure 7H**). The selenium yeast intervention significantly changed the abundance of 25 species compared with the model group. While Acu compared with Se-yeast showed significant changes in the abundance of 7 species. In conclusion, Acu and selenium yeast can have a beneficial effect by modulating the structure of the gut microbiota in EAT rats.

A multi-group comparative analysis of 4 groups was performed at the ASV level based on the obtained community abundance data. The results showed that there were 10 significantly different species in the four groups (**Figure 7I**), among which the contents of ASV1031, ASV125, ASV113 and ASV87 in the model group decreased while increased after Acu intervention (**Figure 7J**). The taxonomic information table of ASV showed that all four belonged to the phylum *Bacteroidota*, ASV125 and ASV113 belonged to the genera *Prevotella*, while ASV1031 and ASV87 belonged to the genera *Muribaculaceae*. These results suggest that *Prevotella* and *Muribaculaceae* may be co-targets for Acu and selenium yeast intervention in EAT.

Combined with thyroid function indexes, inflammatory factors TNF-α and IL-10, the correlation analysis between clinical factors and intestinal microbiota was carried out, and the microbial biomarker with greater correlation with clinical factors was screened through the correlation coefficient between dominant microorganisms and clinical factors. The correlation heat map showed that there were 6 species associated with TPOAb, 4 species associated with TNF-α, and 2 species associated with IL-10, among which *norank_fOscillospiraceae* and *Streptococcus* showed strong correlation with thyroid function and inflammatory factors (**Figure 7K**).

### Combined analysis of intestinal microbiota and metabolomics

**Figure 8:**
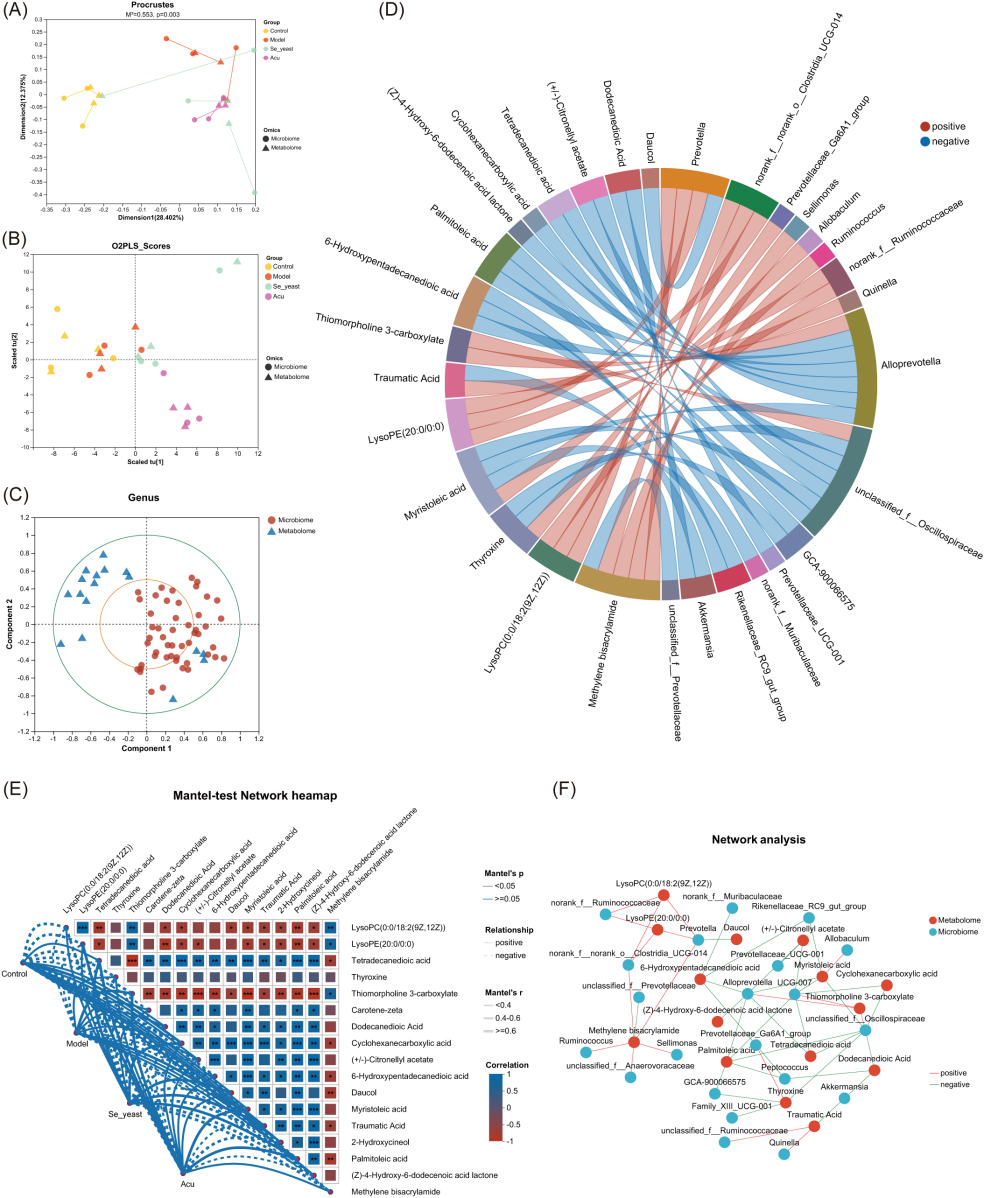
Combined analysis of serum metabolites and gut microbiota. **(A)** Procrustes analysis that the sorting method was PcoA. **(B)** Multiple regression analysis of the O2PLS. **(C)** Canonical correlation analysis based on the top 50 species of relevance. **(D)** Chord diagram based on key metabolites. **(E)** Mantel-test Network heatmap. *P<0.05, **P<0.01, ***P<0.001. **(F)** Network analysis.

Procrustes analysis results indicated that the microbiome abundance and metabolomic expression of the samples were significantly consistent across different groups (M2 = 0.553, P=0.003< 0.01, **Figure 8A**). The results of the multiple regression analysis under the two-way orthogonal partial least squares (O2PLS) were similar to those described above (**Figure 8B**). Canonical correlation analysis (CCA) shows the distribution of the top 50 species with correlations (**Figure 8C**). The Spearman chord diagram was analyzed when the correlation coefficient threshold was 0.3. Each chord in the circle indicates that the metabolites have a significant correlation with the microorganism. Palmitoleic acid is negatively correlated with *Alloprevotilla*, *unclassified_f:Oscillospiraceae*, *GCA-900066575*. Tetradecanedioic acid is negatively correlated with *Alloprevotilla*, *unclassified_f:Oscillospiraceae*. (**Figure 8D**). The Mantel-test heat map shows the correlation of communities and metabolites between different groups through lines of different thicknesses (**Figure 8E**). The network diagram based on genus levels shows the top 50 gut microbes with key metabolites and correlations (**Figure 8F**).

**Figure 9:**
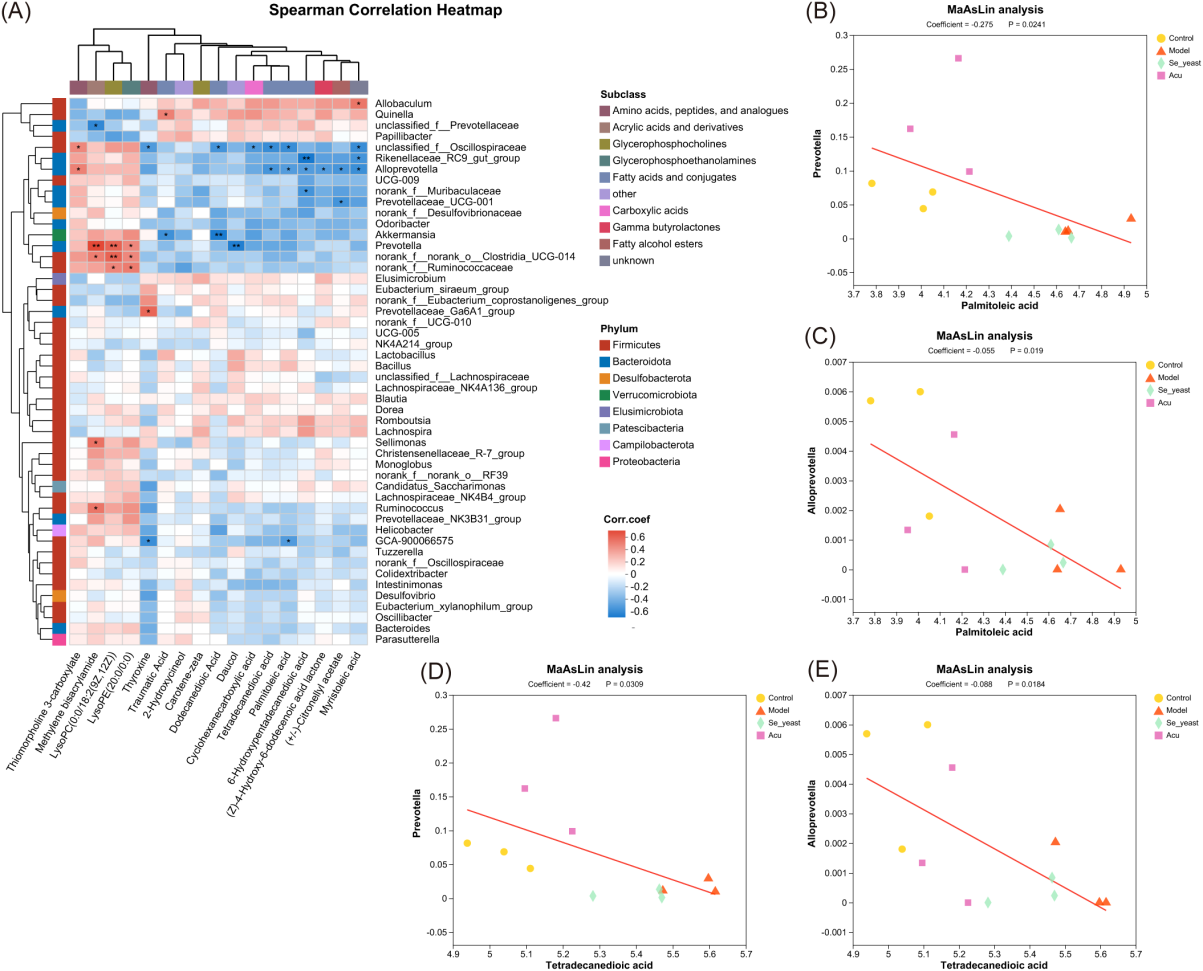
Combined analysis of Specific metabolites and gut microbiota. **(A)** Spearman correlation heatmap based on key metabolites. *P<0.05, **P<0.01. **(B)** MaAsLin analysis of palmitoleic acid with *Prevotella*. **(C)** MaAsLin analysis of palmitoleic acid with *Alloprevotella*. **(D)** MaAsLin analysis of tetradecanedioic acid with *Prevotella*. **(E)** MaAsLin analysis of tetradecanedioic acid with *Alloprevotella*.

The Spearman correlation analysis of specific metabolites and microorganisms revealed that Prevotella was positively correlated with methylene bisacrylamide, lysoPC (0:0/18:2 (9Z,12Z)), and lysoPE (20:0/0:0), while showing a negative correlation with daucol. *Alloprevotella* was positively correlated with thiomorpholine 3-carboxylate, and was negatively correlated with tetradecanedioic acid, palmitoleic acid, 6-Hydroxypentadecanedioic acid, (Z)-4-Hydroxy-6-dodecenoic acid lactone, (+/-)- Citronellyl acetate, myristoleic acid and muribaculaceae (**Figure 9A**). The MaAsLin analysis showed that with the increase of the content of tetradecanedioic acid and palmitoleic acid in the model group, The abundance of microbial *Prevotella* and *Alloprevotella* decreased, which was reversed by Acu and selenium yeast interventions, and microbial abundance increased more in the Acu group (**Figures 9B–E**).

## Discussion

**Figure 10:**
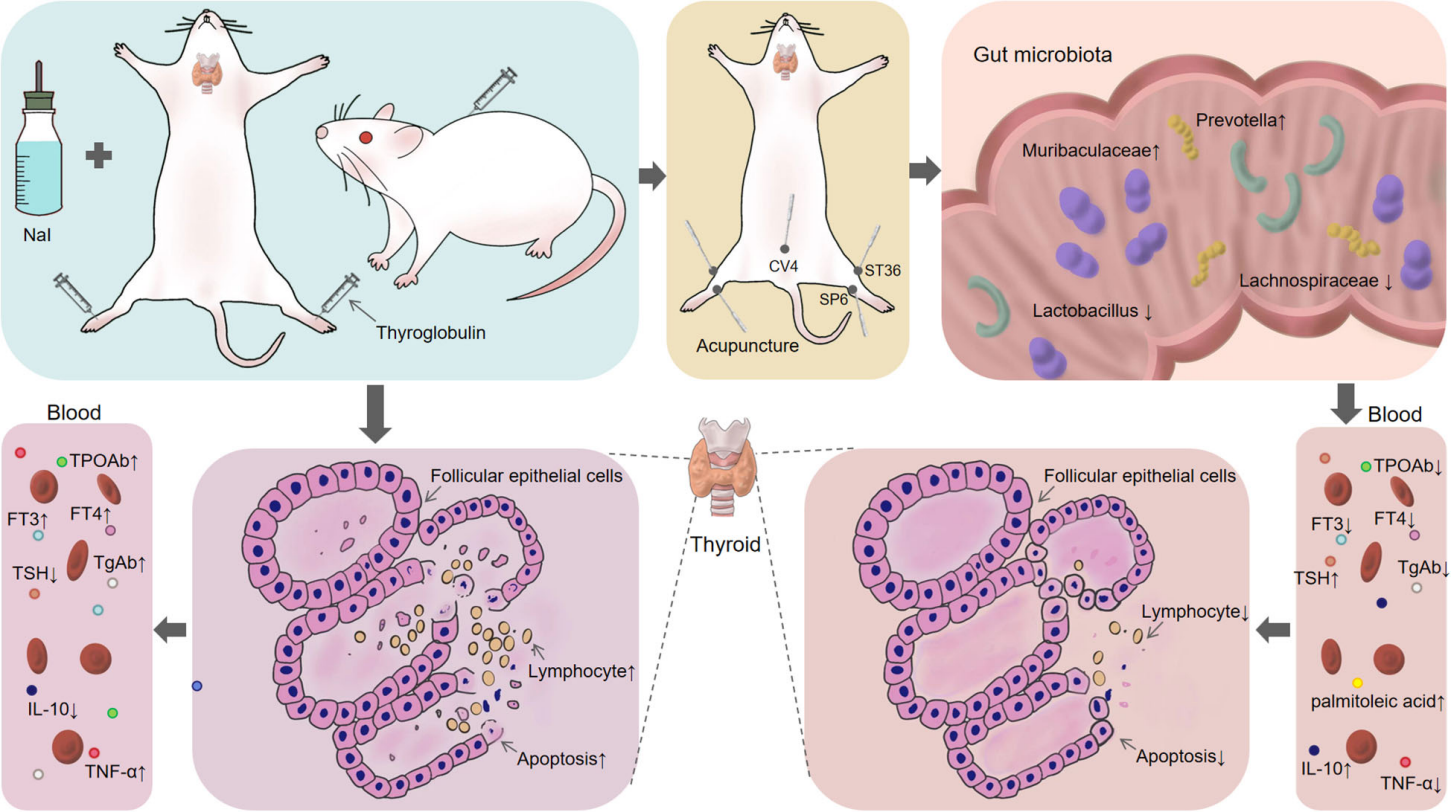
Acu alleviates the apoptosis of thyroid follicular epithelial cells in EAT by regulating intestinal microbiota-palmitoleic acid metabolism.

In recent years, although there are many underlying mechanisms to be further elucidated, the crosstalk between gut microbiota and serum metabolites is still regarded as a critical target for improving metabolic diseases and has received increasing attention from researchers. In this study, we demonstrated that Acu decreased thyroglobulin antibodies, reduced thyroid tissue lymphocyte infiltration, and alleviated thyroid follicular epithelial cell apoptosis in EAT rats (**Figure 10**). This finding provides evidence for the regulatory role of gut microbiota-palmitoleic acid balance on EAT.

### Acu delayed EAT thyroid dysfunction, lymphocyte infiltration, and thyroid follicular epithelial cell apoptosis

AIT is a specific autoimmune disease mediated by T lymphocytes characterized by damage to thyroid follicular epithelial cells (24). Clinical diagnosis was mainly based on thyroid function indexes, TPOAb and TgAb detection. Additionally, FT3 and FT4 increased and TSH decreased during Hashitoxicosis stage (25). The results of this experiment indicated that the serum levels of FT3, FT4, TPOAb and TgAb were increased, the TSH was decreased after modeling. At this time, the EAT rats were in the state of Hashitoxicosis, and acupuncture could relieve this symptom. The pathogenesis of EAT is complex, and it is believed that tissue damage results from autoimmune inflammation, and the mechanism may be related to apoptosis. Apoptosis is a spontaneous death process of normal cells after being stimulated, which plays an important role in the pathogenesis of autoimmune diseases. It is also an important mechanism for the destruction of thyroid follicular cells in AIT patients, and the capital cause of hypothyroidism in the later stage (26).

Acu is a treatment in which needles are inserted into specific Acu points in the body to prevent and treat diseases. In recent years, Acu has demonstrated significant therapeutic efficacy in a range of autoimmune diseases, including rheumatoid arthritis (12), systemic lupus erythematosus (13), and ulcerative colitis (14). In this experiment, we observed that FT3, FT4, TPOAb and TgAb levels decreased and TSH increased after Acu intervention. Under light microscopy, it was found that the uneven glial state in the thyroid follicular cavity in the Acu group was reduced, and the infiltration of stromal inflammatory cells was reduced. Under transmission electron microscopy, it was observed that in the Acu group the nuclear membrane of thyroid follicular epithelial cells was relatively intact, the structure was abnormally reduced, some endoplasmic reticulum was swollen, the intercellular space was acceptable, and the number of microvilli increased. These findings suggest that Acu improved the abnormal thyroid function indexes of EAT rats, reduced lymphocyte infiltration of thyroid tissue, and played a positive role in repairing the structure of thyroid follicles and epithelial cells.

Studies have shown that the underlying mechanisms that mediate the effects of Acu include anti-inflammatory and oxidative stress, inhibition of apoptosis, and others (14, 27). The mitochondrial apoptosis pathway is regulated by the Bcl-2 family, the caspase family, apoptosis-inducing factors, and caspase second mitochondria-derived activators. A large number of studies have shown that Acu can promote the mRNA and protein expression of the Bcl-2, while reducing the mRNA and protein expression levels of caspase-3, and effectively reduce the protein expression levels of Bax and cleaved caspase-3 (28). In the context of autoimmune diseases, Acu has also demonstrated significant anti-apoptotic effects (29). To evaluate the impact of Acu on apoptosis in EAT rats, apoptosis-related targets were assessed using TUNEL staining, IHC and WB. Compared to the control group, the rate of TUNEL-positive apoptotic cells, the positive expression rate of cleaved caspase-3, the content of Bcl-2 decreased, and the contents of Bax, Bcl-2 and the ratio of cleaved caspase-3 to caspase-3 increased, which was reversed by Acu intervention.

### Acu can modulate the disturbance of the metabolic profile of the EAT

To further explore the mechanism by which Acu-enhancing immunity, serum and fecal samples from female EAT rats were analyzed using non-targeted metabolomics analysis and microbiome sequencing methods, which provided insights into the relationship among bacterial populations. These findings may reveal new metabolic targets. The results of this experiment showed that serum metabolism disorders occurred in EAT rats, and Acu significantly affected the serum metabolite profile. After Acu intervention, 17 metabolites were reversed, with their enrichment observed in bile secretion, tyrosine metabolism, fatty acid biosynthesis, neuroactive ligand-receptor interaction, alpha-Linolenic acid metabolism, and thyroid hormone synthesis, thyroid hormone signaling pathway and autoimmune thyroid disease pathway, three of which are directly related to EAT.

The combined analysis of serum metabolites with thyroid function, TPOAb, TgAb, TNF-α and IL-10, revealed that thiomorpholine 3-carboxylate, (+/-)-Citronellyl acetate, mristoleic acid, thyroxine, dodecanedioic acid, and (Z)-4-Hydroxy-6-dodecenoic acid lactone, palmitoleic acid and tetradecanedioic acid exhibited strong correlation with thyroid function, TNF-α and IL-10 at the same time, which may be the targets of Acu in EAT rats.

Most studies have found that palmitoleic acid can regulate immunity and suppress inflammation by inhibiting Th17 cell differentiation (30, 31). Interestingly, other studies of autoimmune diseases have shown the opposite results. Palmitoleic acid expression in feces of patients with autoimmune uveitis is increased, and palmitoleic acid concentrations show a trend associated with the severity of autoimmune uveitis (30). Moreover, the increase in plasma palmitoleic acid promotes benzene-induced inflammation and hematopoietic injury in mice (32). Additionally, palmitoleic acid has been found to promote apoptosis (30). Although recent studies on palmitoleic acid and health are inconsistent, the results of this experiment confirm that Acu can reduce the content of palmitoleic acid in serum of EAT rats, slow down the apoptosis of thyroid follicular epithelial cells, and alleviate autoimmune inflammation. Palmitoleic acid has been found to cause apoptosis of adipocytes (33), which is consistent with the results of this study, in which palmitoleic acid levels were elevated in EAT rats, and Acu reversed this phenomenon. Taken together, these results suggest that Acu regulates serum metabolic profile and subsequent metabolite synthesis, particularly palmitoleic acid, through circulation affecting autoimmune thyroiditis.

### Acu can modulate intestinal microbiota disturbances in EAT

The microbiome regulates the transcriptome and metabolome of the host, and the abundance of the gut microbiota is an important reference to reflect the intestinal health and immunity, which has a profound impact on the physiology of the host. Recent research has shown that autoimmune thyroiditis is associated with dysregulation of the gut microbiome, which is attributed to the interaction between the hypothalamic-pituitary-thyroid (HPT) axis and the gut microbial composition (34). In our study, we utilized high-iodine water during the modeling process. Excess iodine intake was found to exacerbate autoimmune thyroiditis by reducing apoptosis of thyroid follicular cells, thereby promoting disease progression (35, 36). Moreover, excessive iodine intake not only impacts the thyroid gland directly but also alters the gut microbiome composition, leading to changes in the microbiome-gut-thyroid axis metabolism in autoimmune thyroiditis (37). The results of this study indicate that *Firmicutes*, *Bacteroidota*, and *Spirochaetota* are the dominant intestinal species in rats, which is consistent with the current study (38). *Prevotella* belongs to the genus *Bacteroidota* and is often considered a probiotic of the gut. It has also been shown to downregulate caspase-3 and caspase-8 and improve the inflammatory response in ovariectomized mice via gut microbiota-dependent (39). The results of this experiment are consistent with this. After Acu and selenium yeast intervention, *Prevotella* increased, restraining apoptosis of follicular cells of the thyroid gland. The results showed that *unclassified_f:Lachnospiraceae*, *norank_f:Muribaculaceae*, *unclassified_f:Prevotellaceae* and *Lactobacillus* were the dominant strains. The relative contents of *unclassified_f:Lachnospiraceae*, *unclassified_f:Prevotellaceae* and *Lactobacillus* increased and the *norank_f:Muribaculaceae* decreased significantly in the model groups. While the content was significantly reversed after Acu intervention. The comparative analysis of multiple groups showed that the contents of *Prevotella*, *norank_f:norank_o:Clostridia_vadinBB60_group* and *Peptococcus* in the model group decreased while increased after Acu treatment. Among them, *Prevotella* changed significantly between the control group and the model group, suggesting that it may be the target of Acu intervention in the intestinal microbiota of EAT rats.

### Acu improves the thyroid inflammatory response in EAT rats by modulating Palmitoleic acid/*Prevotella*

Zhang, L et al. (32) found that abnormally elevated levels of palmitoleic acid can exacerbate interleukin-5 (IL-5)-mediated immune inflammation and hematopoietic damage by disrupting in the gut microbiota. Acu at ST36, CV4, and SP6 points can mitigate autoimmune and metabolic diseases by regulating the content of metabolites and the abundance of gut microbes (40–43). Acu can increase the abundance of microbiota in *Prevotella*, reduce the pro-inflammatory factor TNF-α, and increase the content of the protective inflammatory factor IL-10, thereby reducing the infiltration of inflammatory cells (44). This study utilized Acu at ST36, CV4 and SP6 to investigate their intervention effects on EAT rats. The combined analysis of serum metabolites and fecal metabolomics showed that the serum TNF-α increased, IL-10 decreased, the serum metabolite Palmitoleic acid content increased, and the abundance of fecal microorganisms *Prevotella* and *Alloprevotella* decreased. *Prevotella* may be one of the axes of action for Acu to improve the thyroid inflammatory response in EAT rats.

### There are co-metabolites and gut microbial targets for the intervention of Acu and selenium yeast in EAT rats

Selenium (Se) is a crucial trace element that plays an important role in thyroid physiology. Selenium supplementation has been found to reduce the level of autoimmune thyroid antibodies and attenuate thyroid follicular damage during EAT (45). In this study, selenium yeast gavage intervention was used as the positive control group, and serum metabolomics and intestinal microbiota analysis were carried out together with Acu intervention. The results showed that compared with the control group, the expression levels of cyclohexanecarboxylic acid and tetradecanedioic acid in the model group increased, and the expression levels of selenium yeast and Acu decreased after Acu intervention. This suggests that cyclohexanecarboxylic acid and tetradecanedioic acid may serve as common serum metabolite targets for Acu and selenium yeast interventions in autoimmune thyroiditis.

Specifically, 16S rRNA gene sequence analysis showed that *Firmicutes*, *Bacteroidota* and *Spirochaetota* were the dominant strains at the phylum level. Compared with the control group, the relative content of *Desulfobacterota* in the model group decreased and increased after Acu intervention. At the genus level, *Lachnospiraceae*, *Muribaculaceae*, *Prevotellaceae* and *Lactobacillus* were the dominant strains, and the relative content of *Lachnospiraceae* in the model group was higher than that in the control group, but decreased after Acu and selenium yeast intervention. Acu increased the contents of *Ruminococcaceae*, *Sellimonas*, *Peptococccaceae*, *Family_XIII_AD3011_group* and *Butyricicoccus* in the intestinal tract of EAT rats, but decreased the content of *Veillonella*, indicating that Acu intervention played an important role in regulating the abundance and structure of intestinal microbiota in EAT rats. The results of the comparative analysis of the four groups showed that *Prevotella* and *Muribaculaceae* were differential species, and combined with the previous analysis, *Prevotella* may be a microbial biomarker with a greater correlation between Acu and selenium yeast intervention in EAT. The correlation heat map of thyroid function indexes, inflammatory factor TNF-α, IL-10 and intestinal microbiota showed that *Oscillospiraceae* displayed a notable correlation with TPOAb, thyroid function and inflammatory factor TNF-α at the same time, but the difference was not large, which may be due to the small sample size and large individual differences.

This experiment has several limitations. Firstly, the changes in the serum metabolic profile and intestinal microbiota were observed in the control group, model group, Se-yeast group and Acu group. Additionally, this study also studied the regulatory effect of Acu and selenium yeast on apoptosis of rat thyroid follicular epithelial cells, and explored the metabolomic changes of fecal and serum samples in EAT rats before and after modeling and intervention, but the combination of multi-locus and multi-omics studies can more accurately determine the regulatory network related to EAT development. Therefore, in future studies, multiple analytical methods should be employed to identify more metabolic pathways associated with Acu-mediated EAT intervention, which will be validated for metabolite or microbiota targets in the next experiments.

## Conclusion

This study is the first to investigate the underlying mechanism of Acu anti-EAT dysfunction by integrating 16s rRNA sequencing, multi-metabolomics, and apoptosis analysis. The findings indicate that Acu could effectively improve thyroid dysfunction and thyroid histopathological changes in EAT rats, and had a regulatory effect on intestinal microbiota dysbiosis and serum metabolism disorders in EAT rats. Interestingly, this study found a link between palmitoleic acid and *Prevotella*, and Acu could reduce the serum metabolite palmitoleic acid content and increase the abundance of fecal microorganisms *Prevotella* and *Alloprevotella*, which may be a key factor in the effect of Acu on EAT intervention. Metabolites (cyclohexanecarboxylic acid, tetradecanedioi acid) and microbiota (*Prevotella*) may be the co-targets of Acu and selenium yeast intervention in EAT. In summary, the results of this experimental study imply that Acu as a therapeutic means can prevent the development of EAT through microbiome-metabolite profile-inflammation-apoptosis. The use of multi-omics, spatial transcriptomics and spatial metabolomics analysis to explore the exact mechanism of Acu-mediated EAT therapy requires further research.

## Data Availability

The data presented in the study are deposited in the Figshare repository, accession number of metabolomics: https://doi.org/10.6084/m9.figshare.28801472.v2 and intestinal microorganisms: https://doi.org/10.6084/m9.figshare.28801577.v1.
