# Supplementary figures and images for: Acupuncture attenuates experimental autoimmune thyroiditis by modulating intestinal microbiota and palmitic acid metabolism
Source: Front Immunol. 2025 Apr 28;16:1541728. doi: 10.3389/fimmu.2025.1541728 (PMC12066539; doi:10.3389/fimmu.2025.1541728)

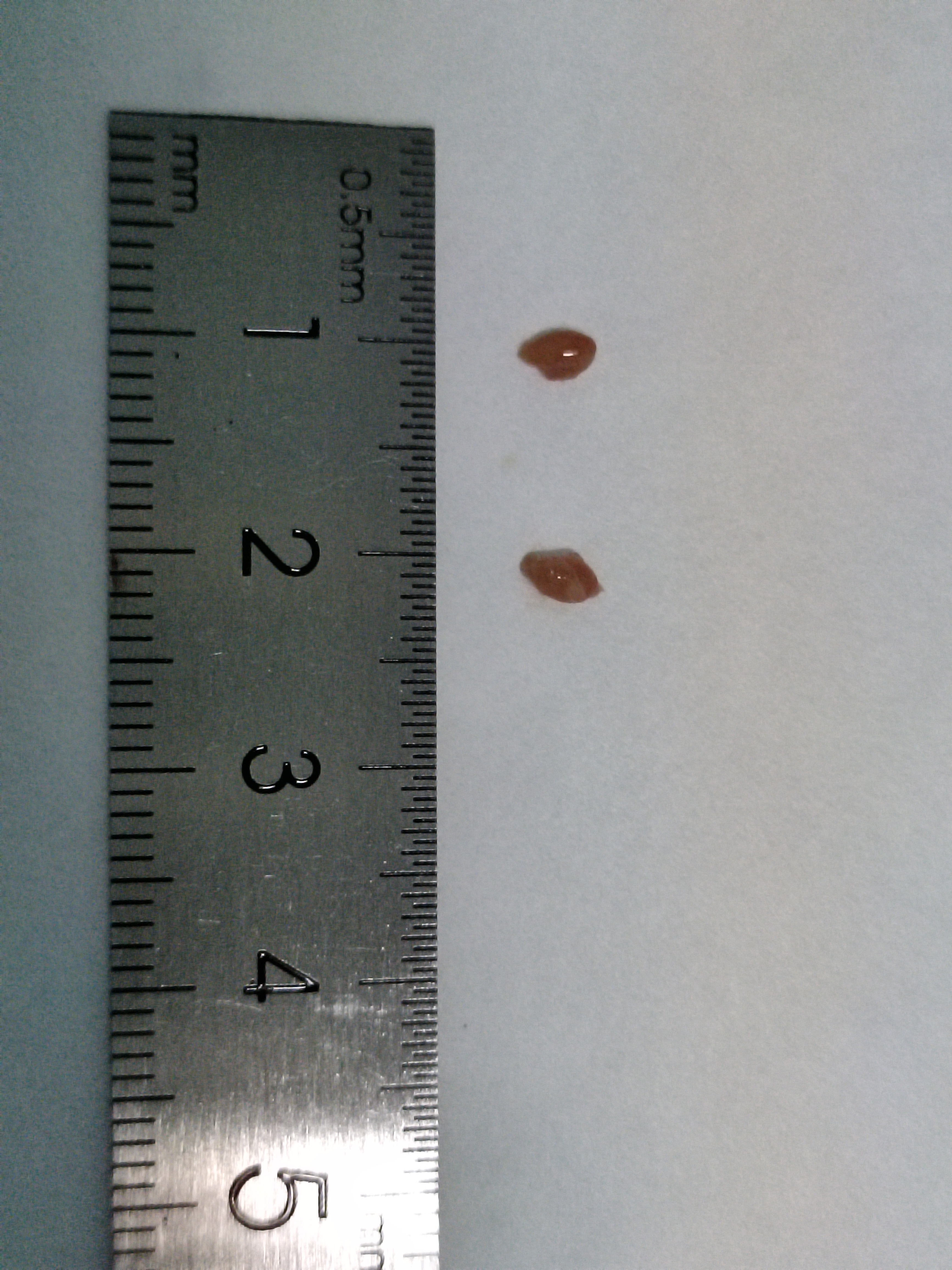

Supplement: Supplementary file 2 [file DataSheet2.zip › thyroid images/Acu.jpg]

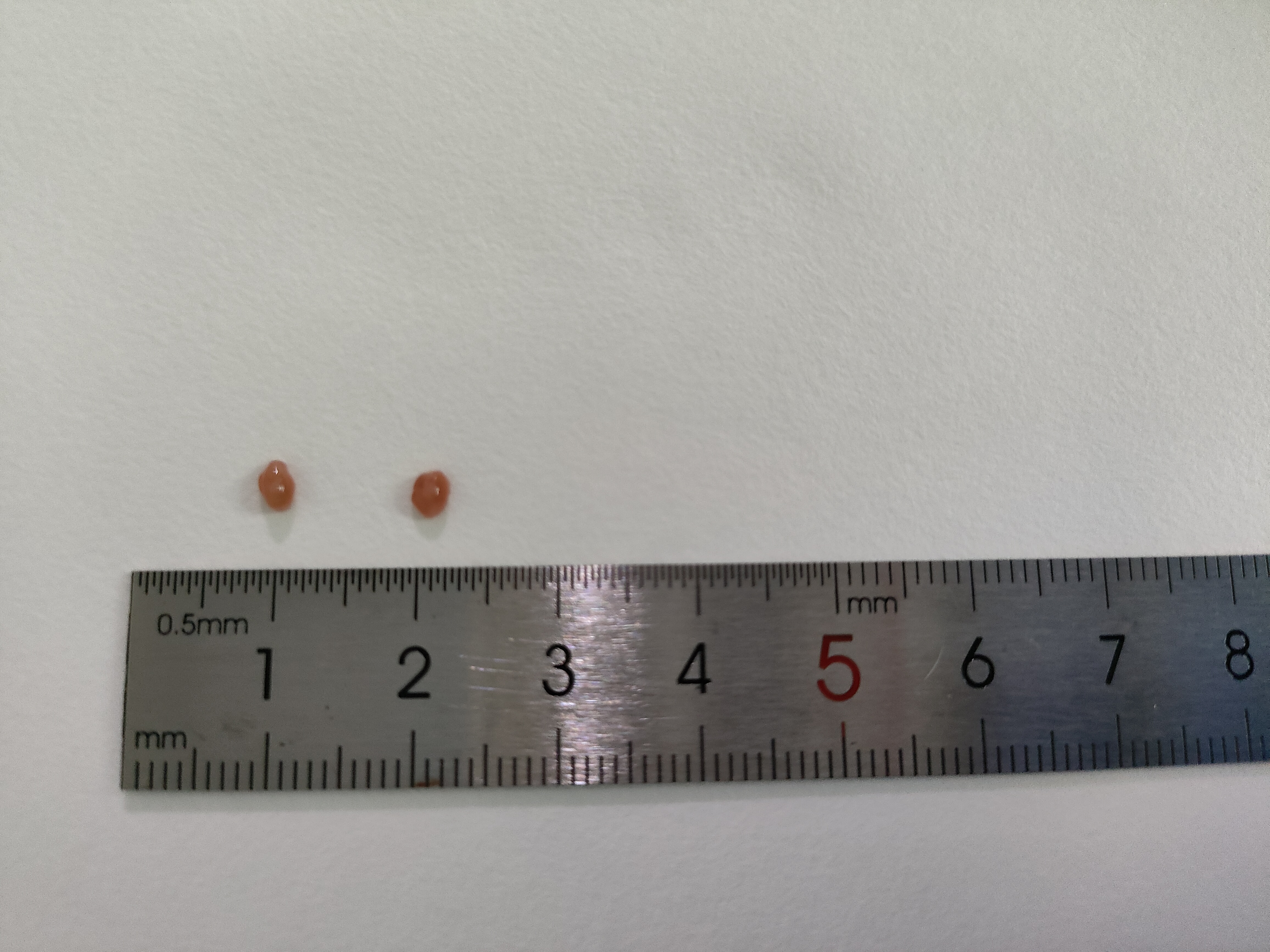

Supplement: Supplementary file 2 [file DataSheet2.zip › thyroid images/Control.jpg]

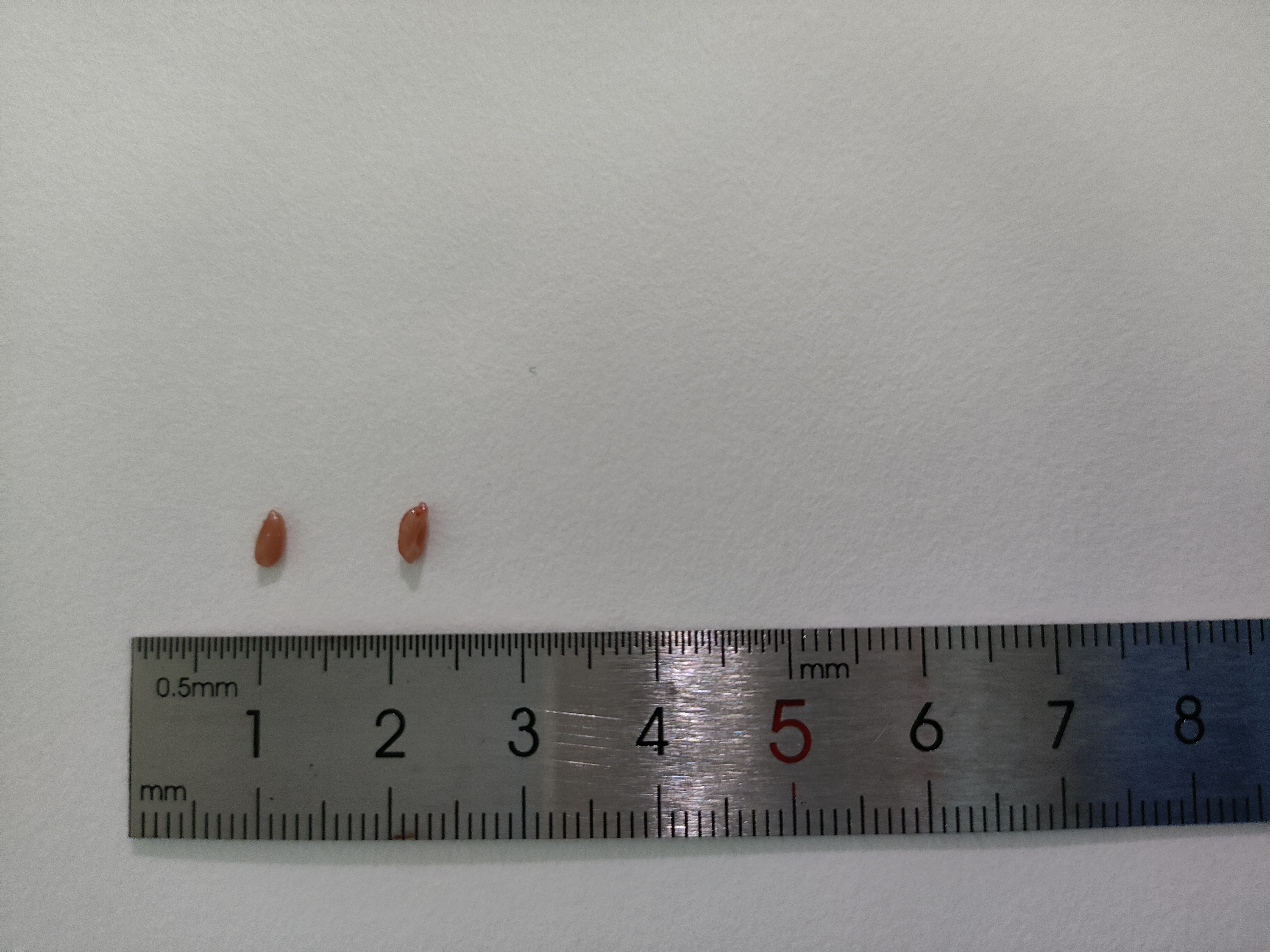

Supplement: Supplementary file 2 [file DataSheet2.zip › thyroid images/Model.jpg]

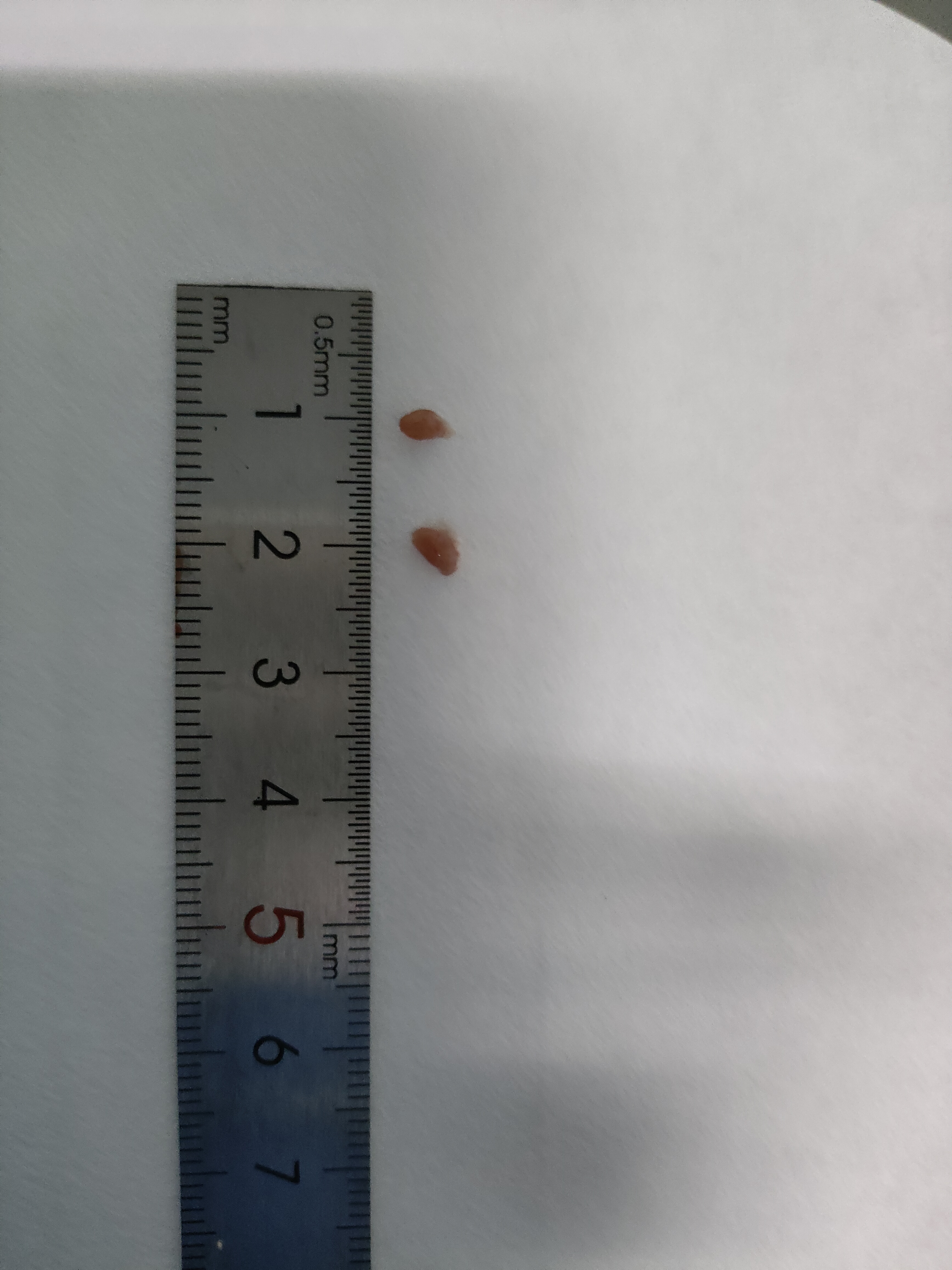

Supplement: Supplementary file 2 [file DataSheet2.zip › thyroid images/Se-yeast.jpg]

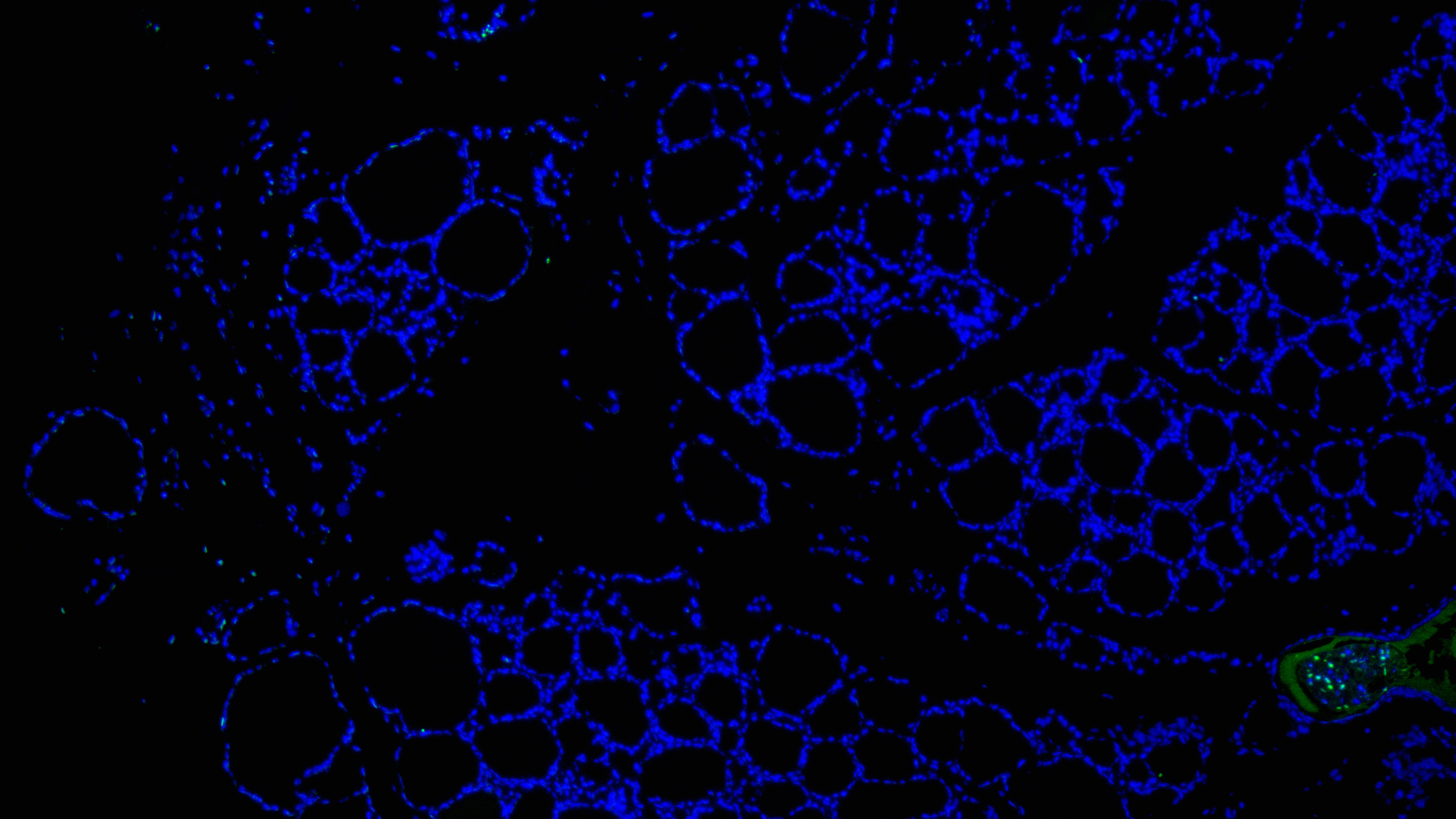

Supplement: Supplementary file 3 [file DataSheet3.zip › TUNEL images 1/A3 JZ TUNEL100-3+4.tif]

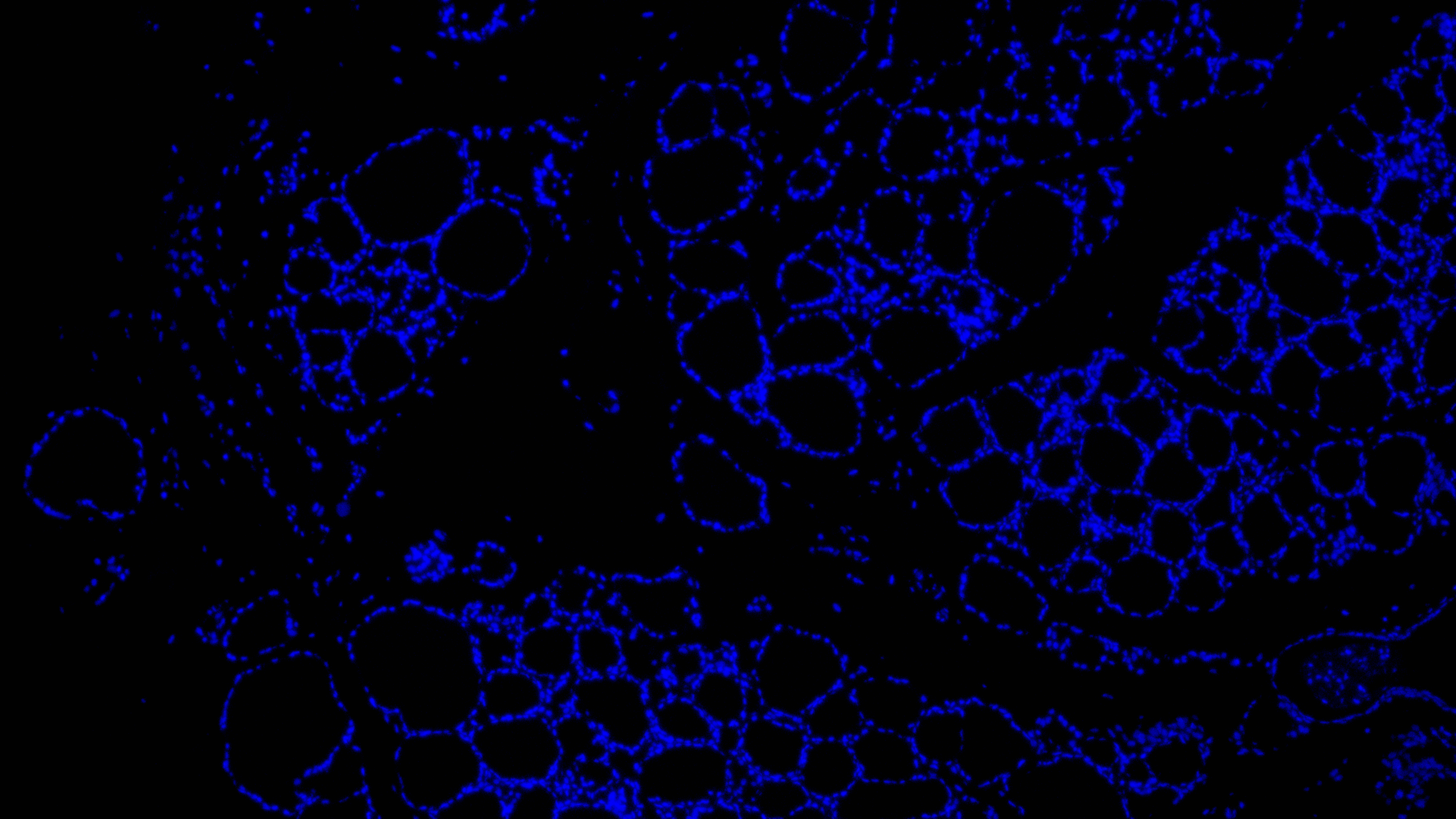

Supplement: Supplementary file 3 [file DataSheet3.zip › TUNEL images 1/A3 JZ TUNEL100-3.tif]

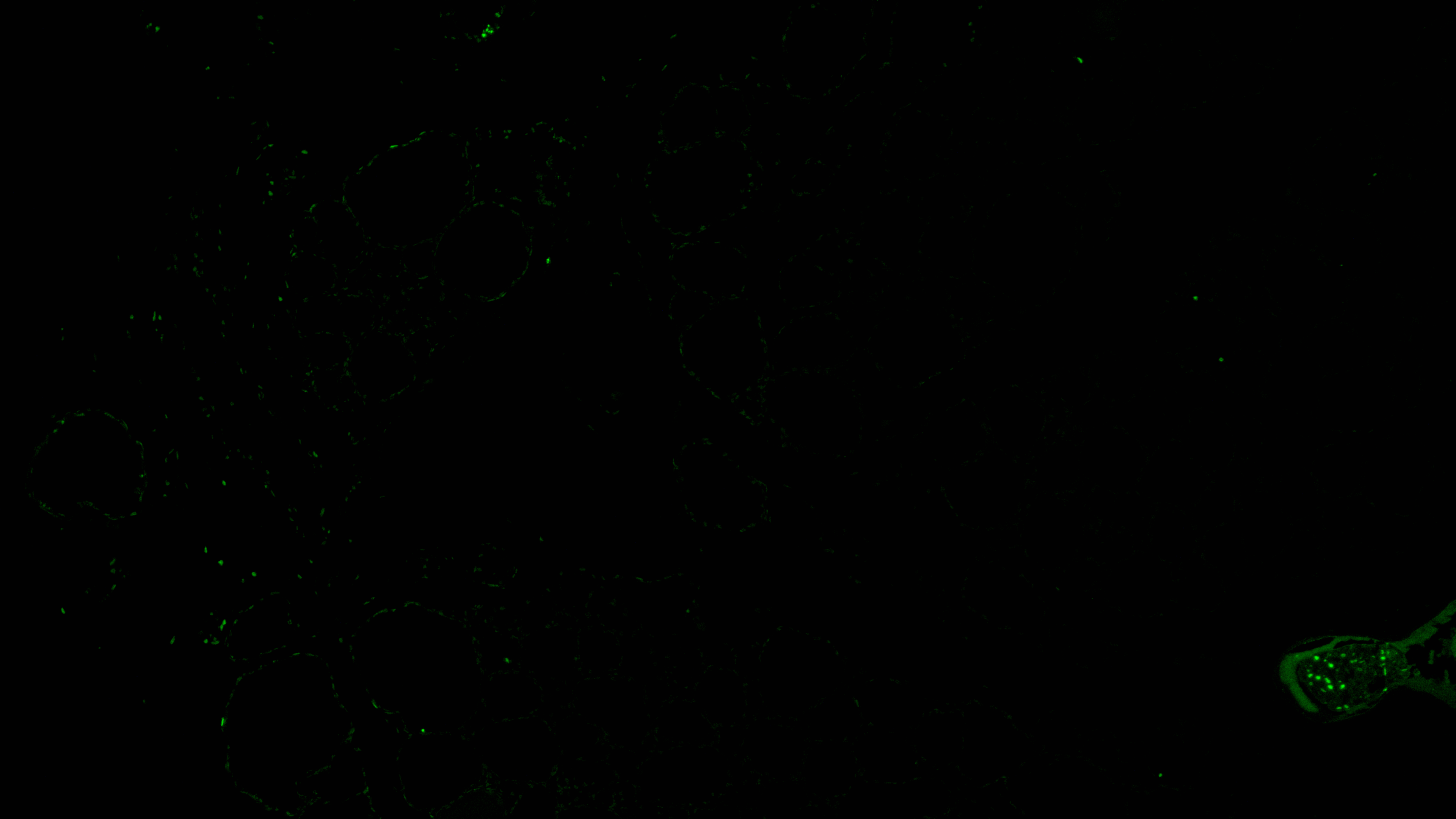

Supplement: Supplementary file 3 [file DataSheet3.zip › TUNEL images 1/A3 JZ TUNEL100-4.tif]

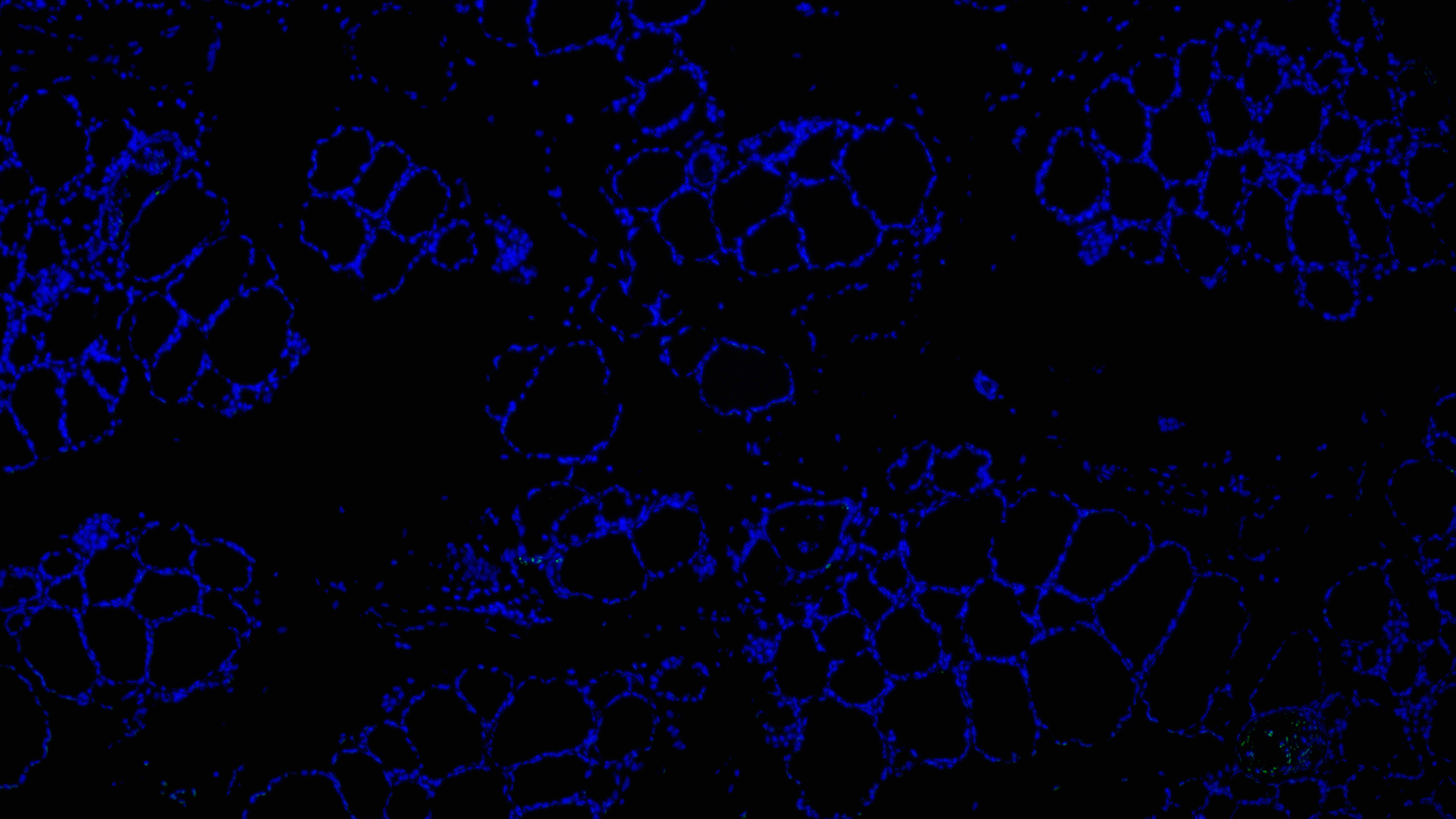

Supplement: Supplementary file 3 [file DataSheet3.zip › TUNEL images 1/C1 JZ TUNEL100-1+2.tif]

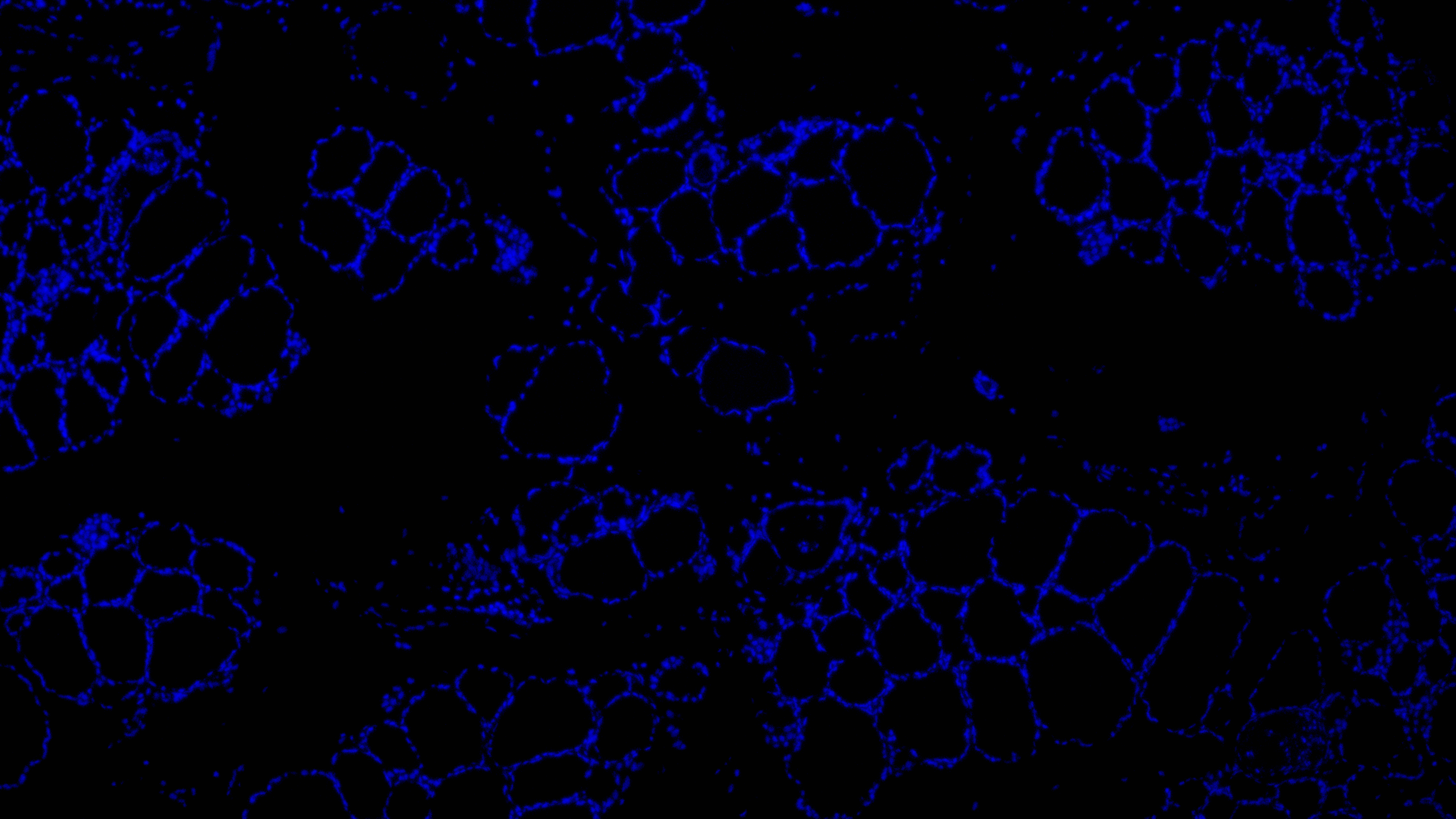

Supplement: Supplementary file 3 [file DataSheet3.zip › TUNEL images 1/C1 JZ TUNEL100-1.tif]

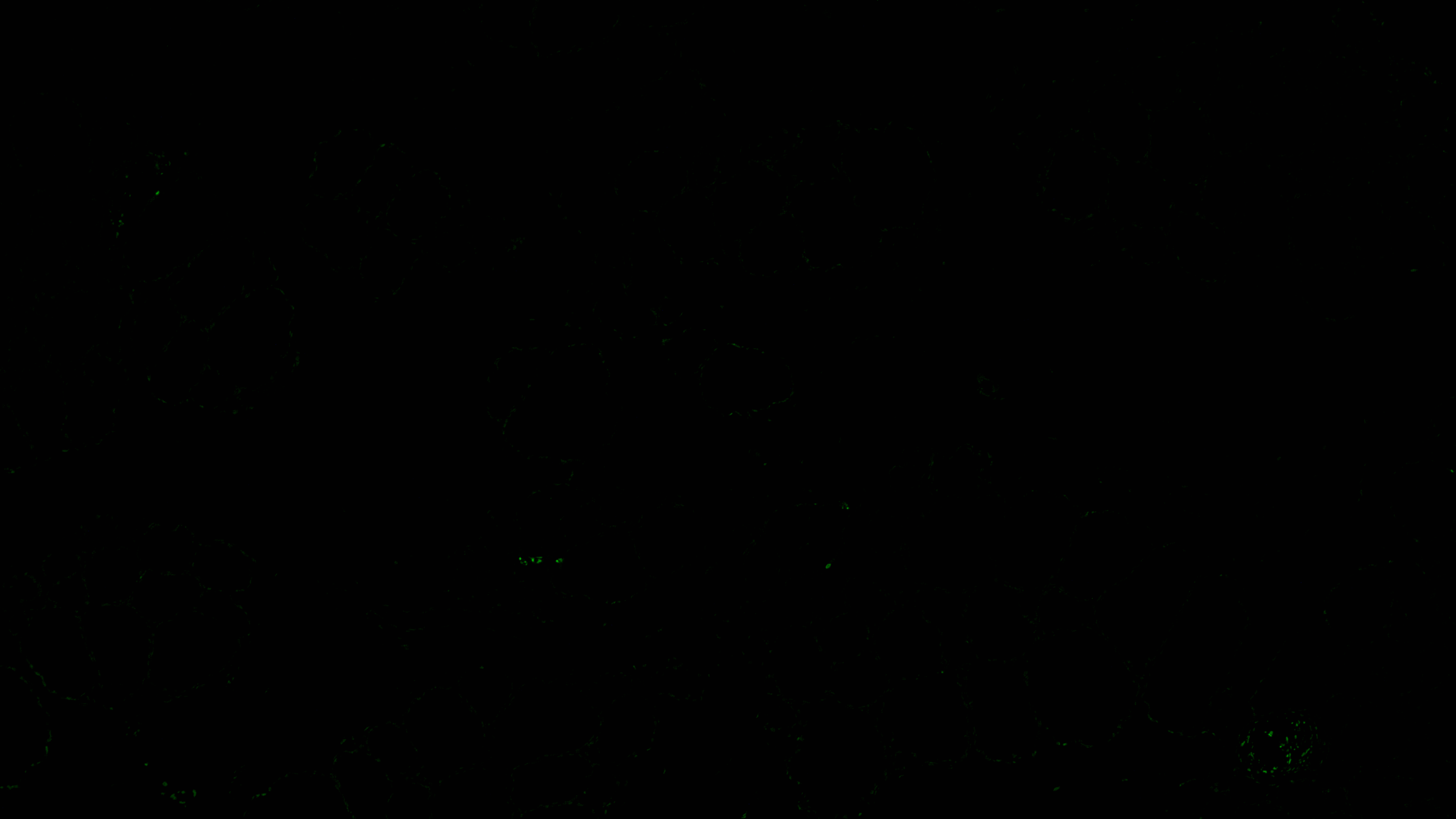

Supplement: Supplementary file 3 [file DataSheet3.zip › TUNEL images 1/C1 JZ TUNEL100-2.tif]

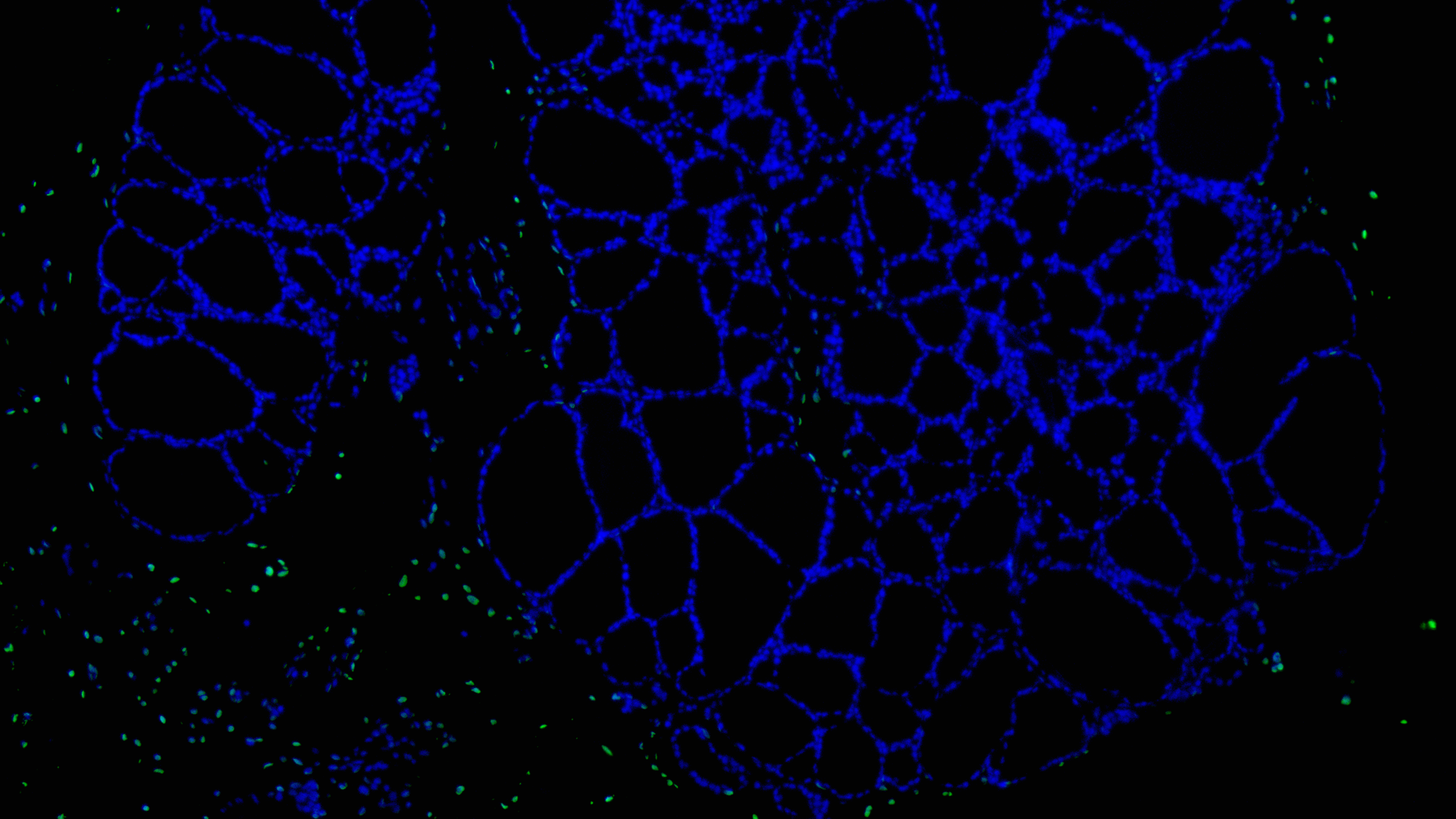

Supplement: Supplementary file 4 [file DataSheet4.zip › TUNEL images 2/M1 J-2 TUNEL100-1+2.tif]

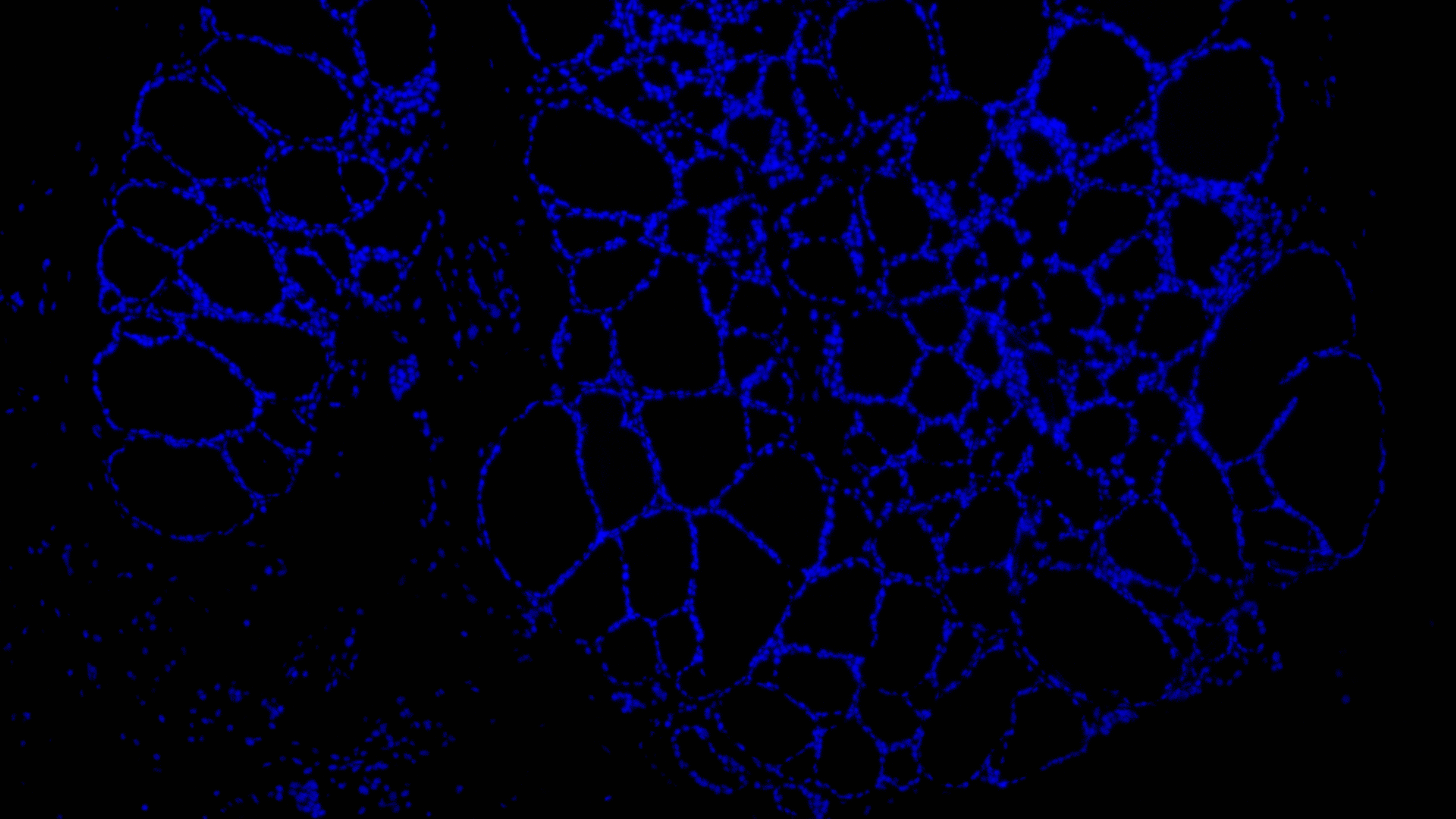

Supplement: Supplementary file 4 [file DataSheet4.zip › TUNEL images 2/M1 J-2 TUNEL100-1.tif]

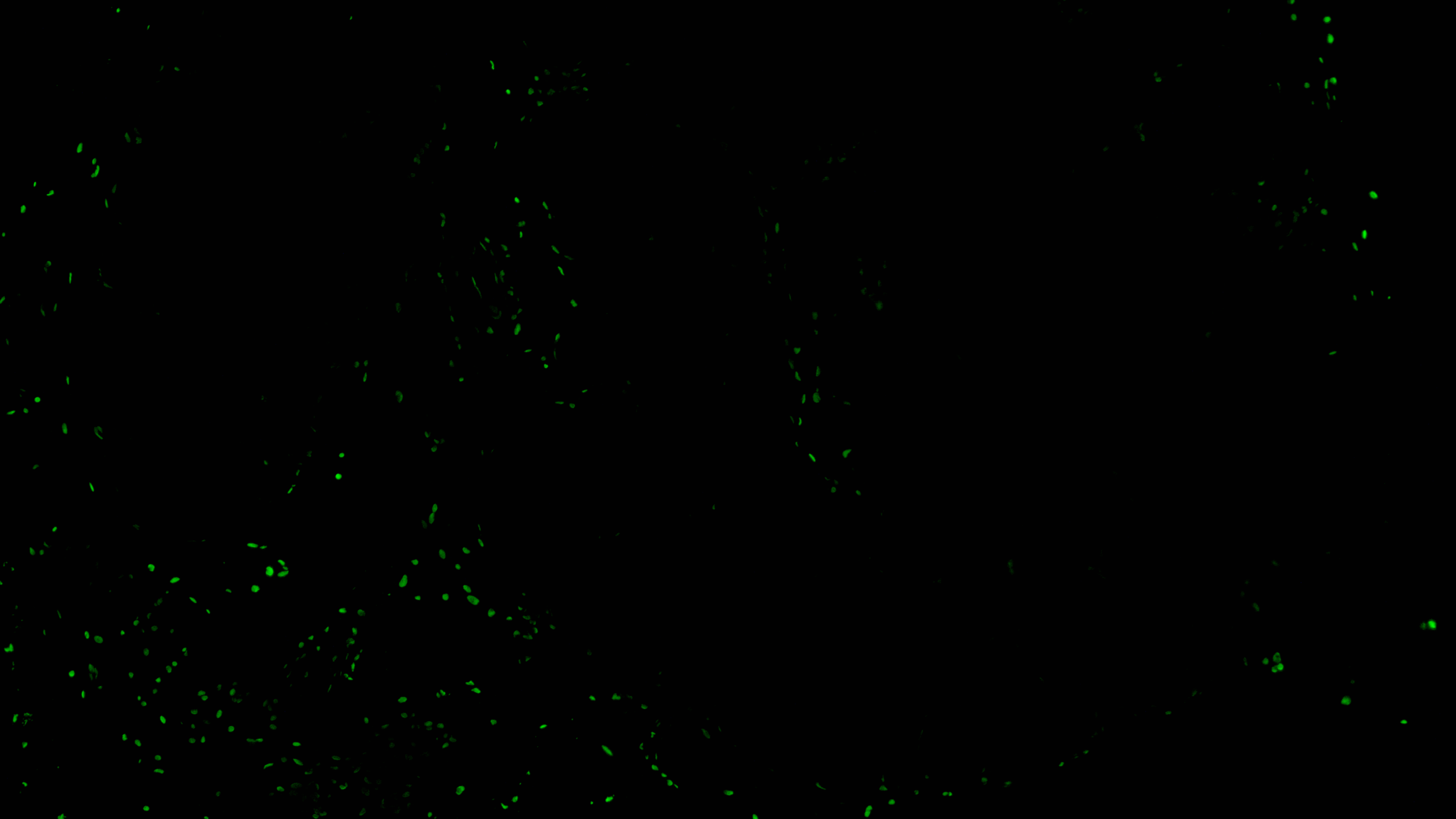

Supplement: Supplementary file 4 [file DataSheet4.zip › TUNEL images 2/M1 J-2 TUNEL100-2.tif]

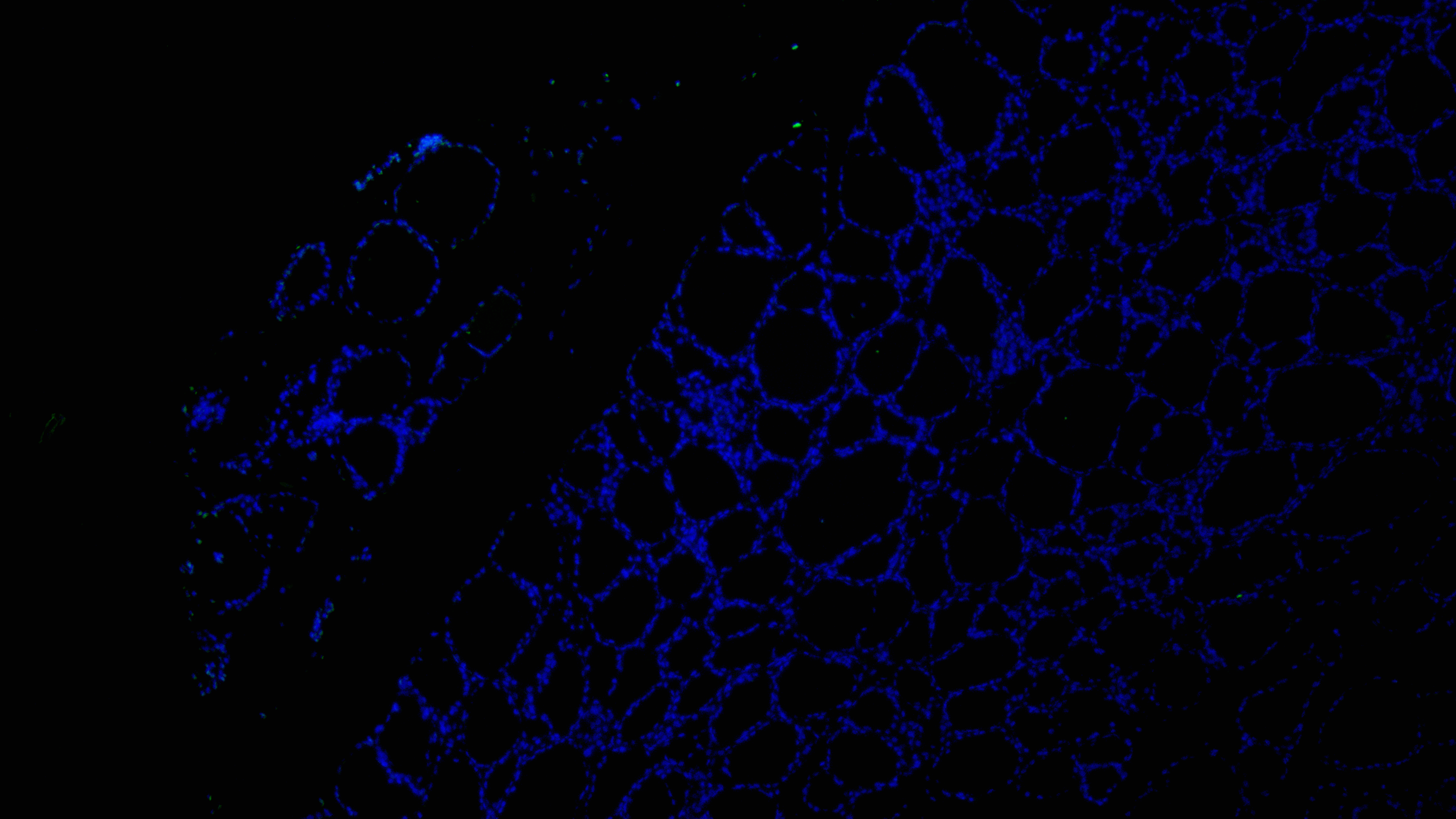

Supplement: Supplementary file 4 [file DataSheet4.zip › TUNEL images 2/X1 JZ TUNEL100-1+2.tif]

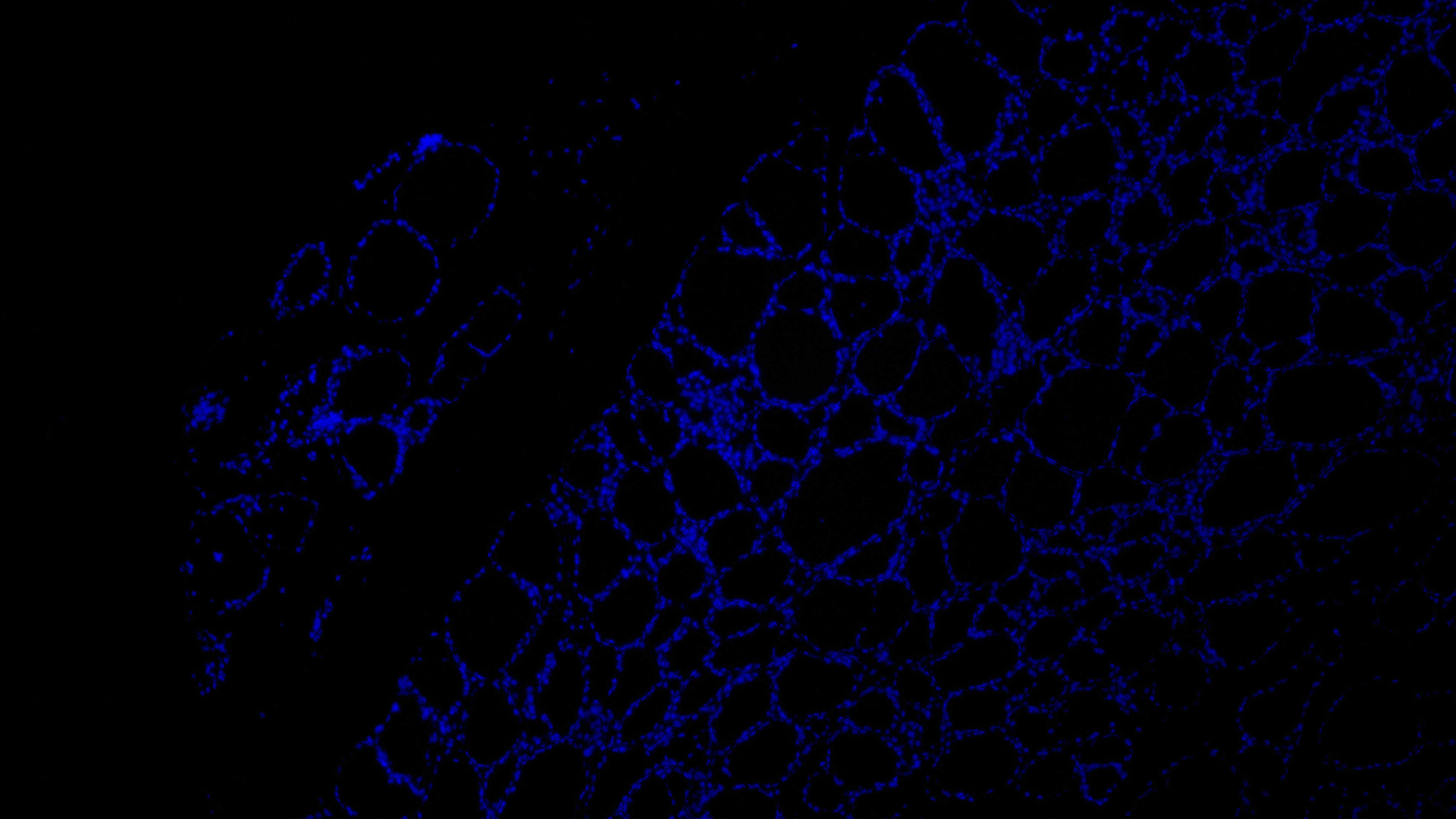

Supplement: Supplementary file 4 [file DataSheet4.zip › TUNEL images 2/X1 JZ TUNEL100-1.tif]

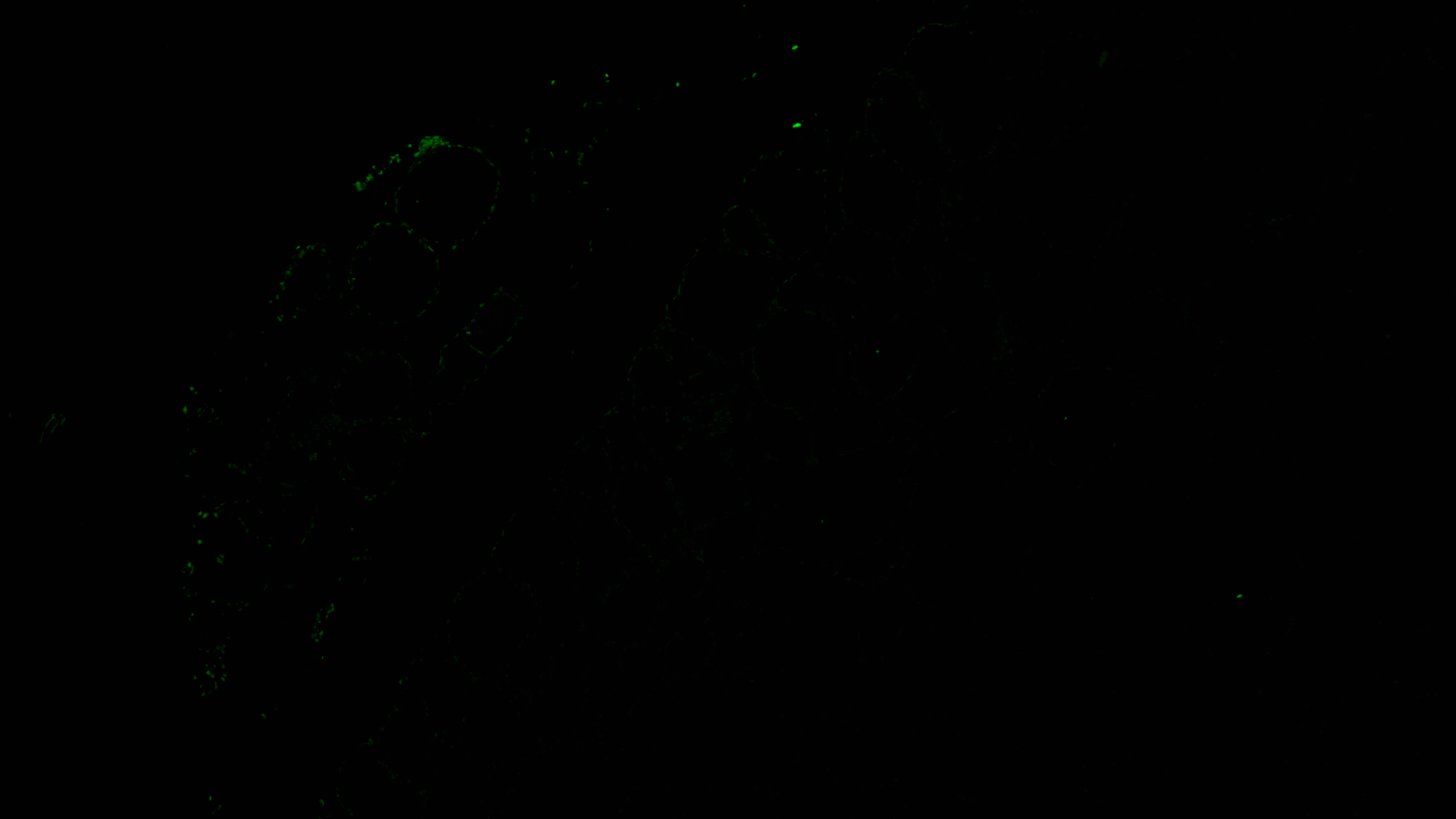

Supplement: Supplementary file 4 [file DataSheet4.zip › TUNEL images 2/X1 JZ TUNEL100-2.tif]

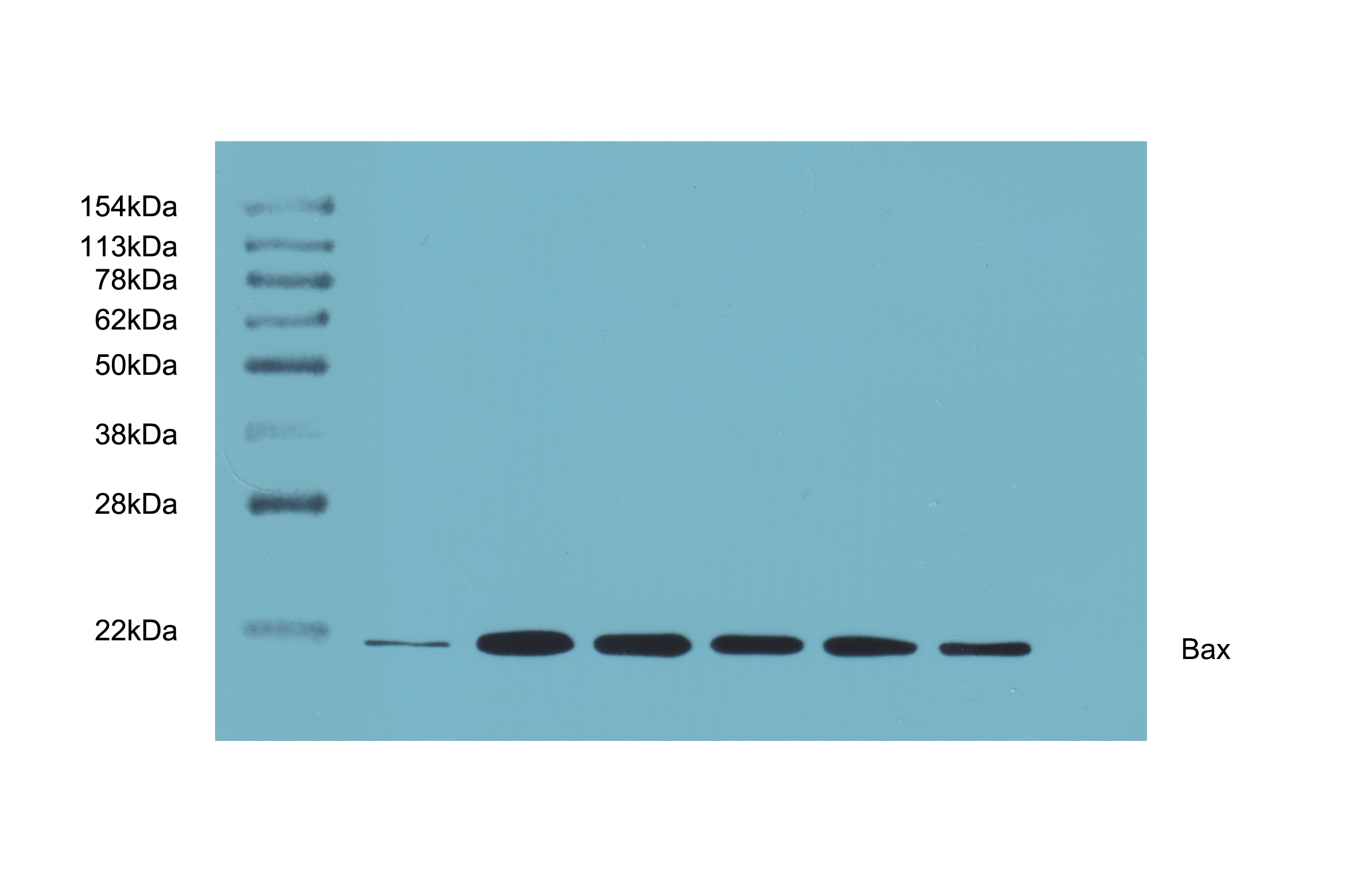

Supplement: Supplementary file 5 [file DataSheet5.zip › Western Blot_original gels 1/1/Western blot_Bax.tif]

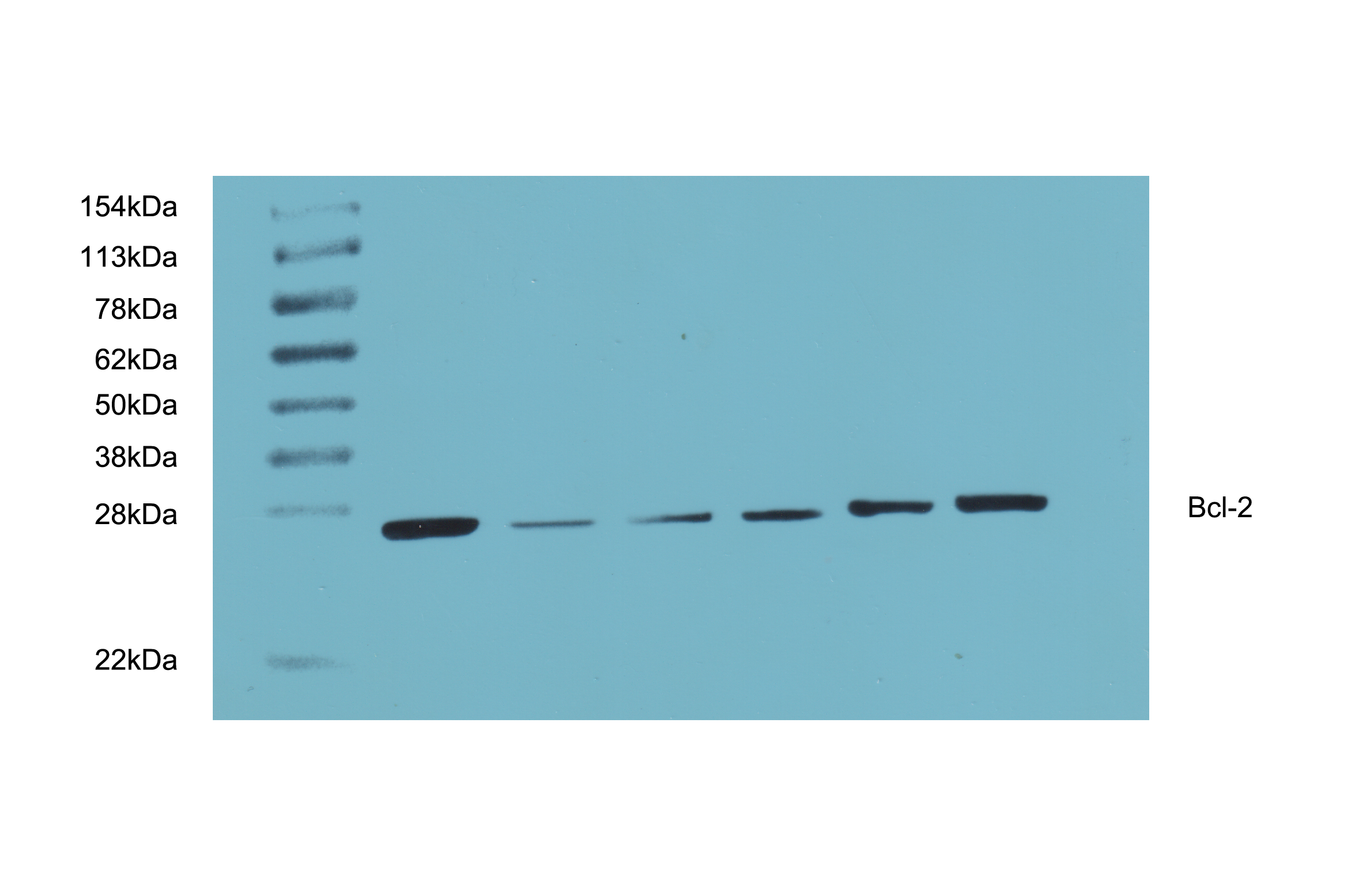

Supplement: Supplementary file 5 [file DataSheet5.zip › Western Blot_original gels 1/1/Western blot_Bcl-2.tif]

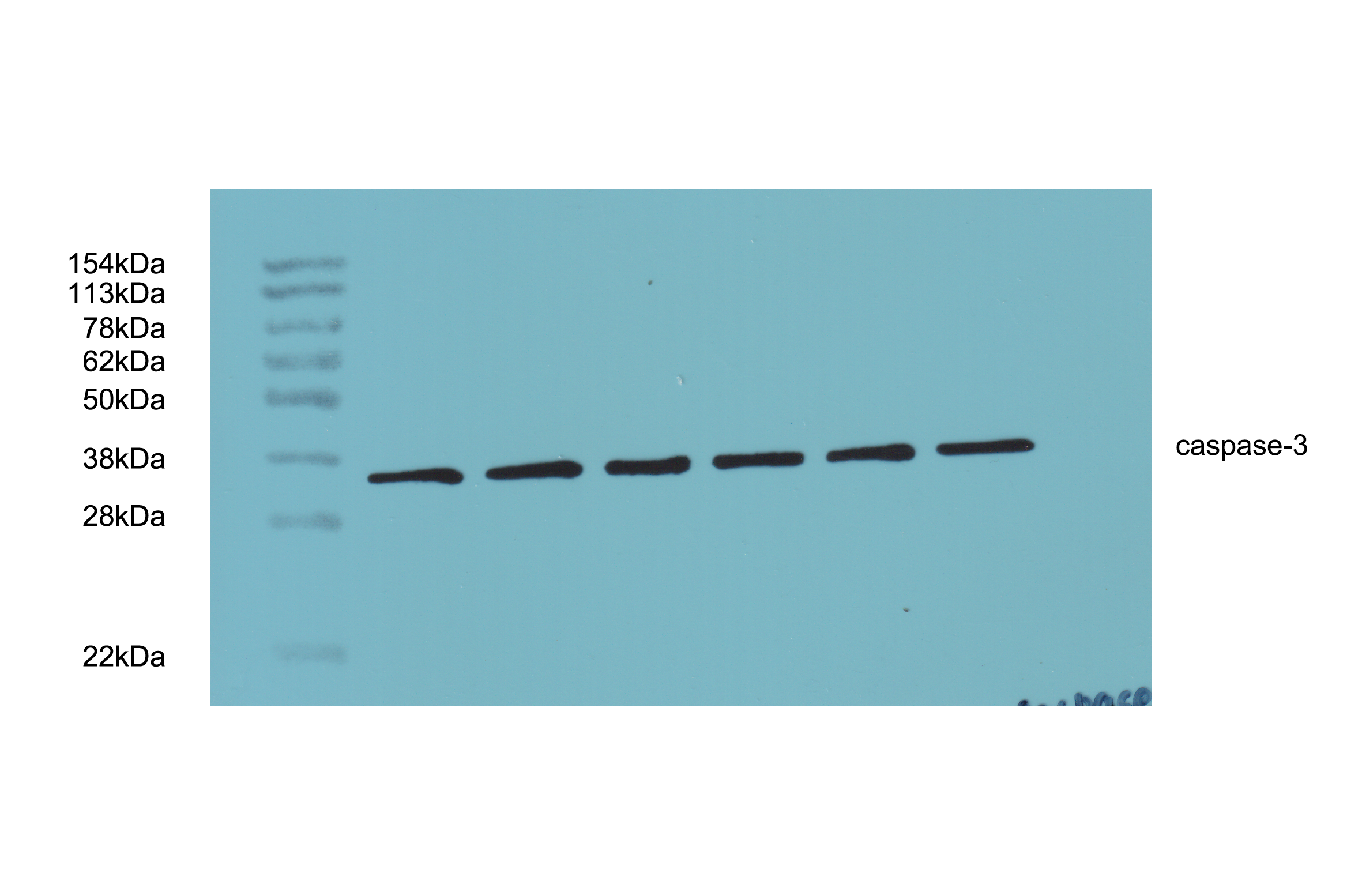

Supplement: Supplementary file 5 [file DataSheet5.zip › Western Blot_original gels 1/1/Western blot_caspase-3.tif]

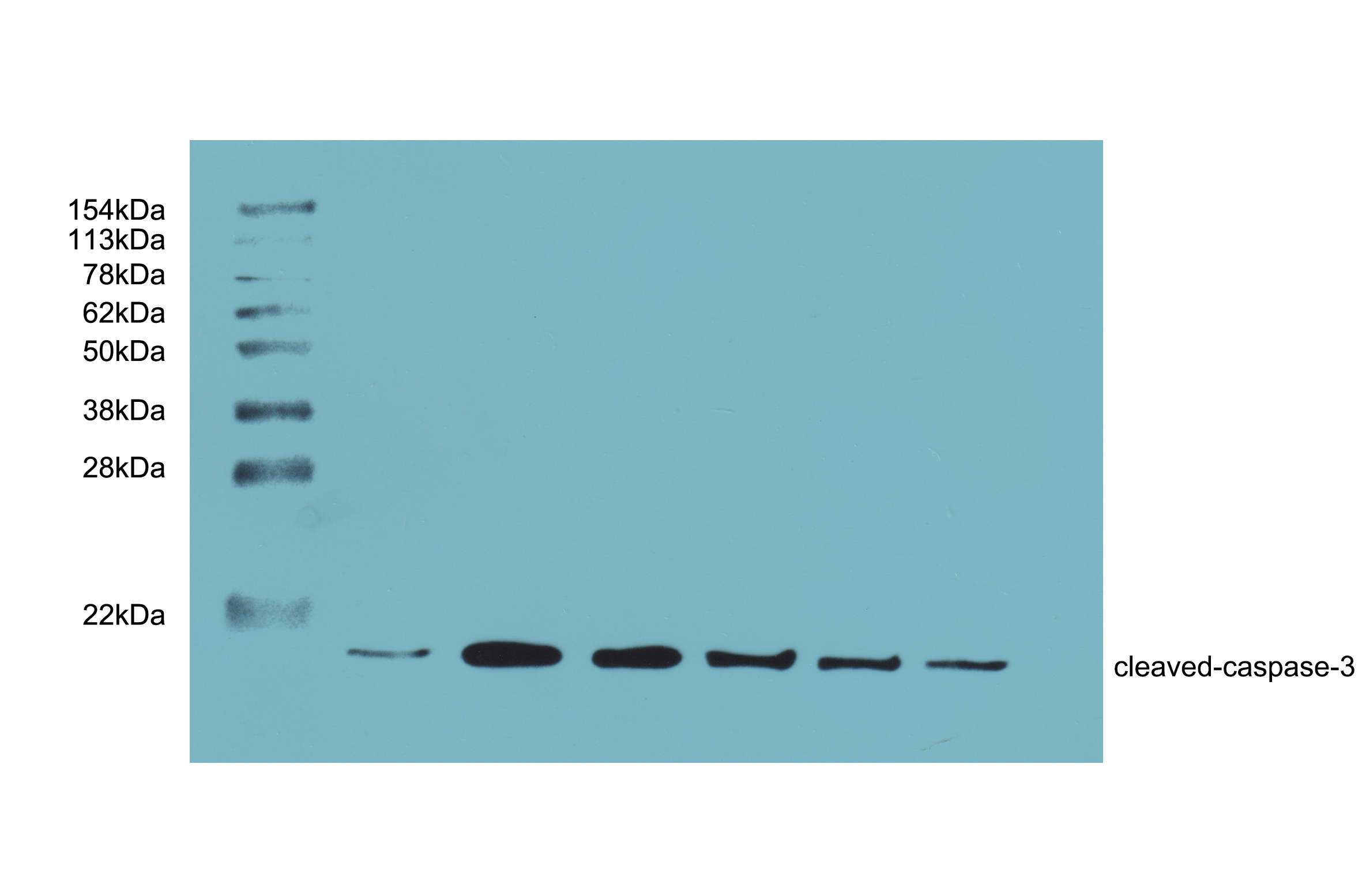

Supplement: Supplementary file 5 [file DataSheet5.zip › Western Blot_original gels 1/1/Western blot_cleaved-caspase-3.tif]

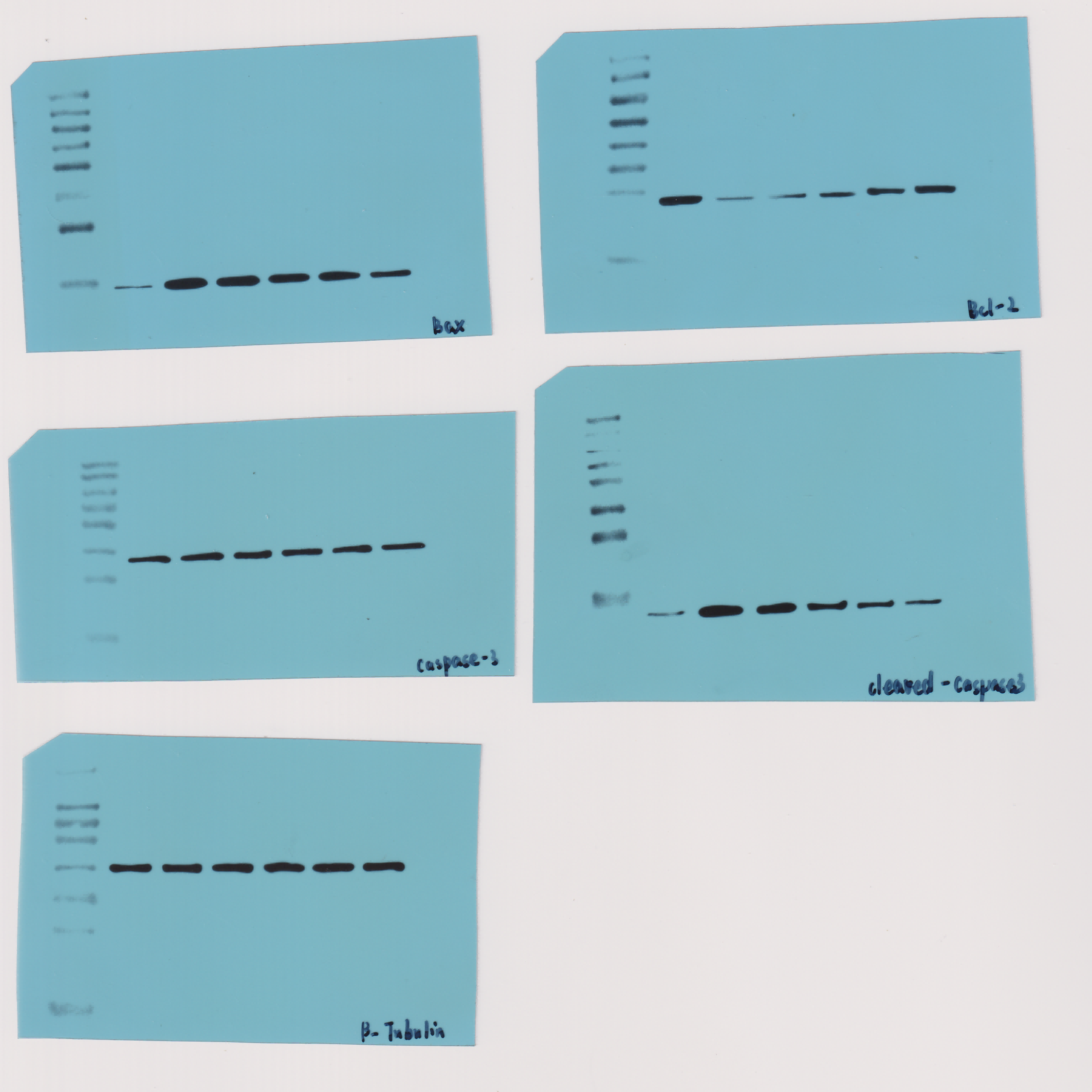

Supplement: Supplementary file 5 [file DataSheet5.zip › Western Blot_original gels 1/1/Western blot_total(1).png]

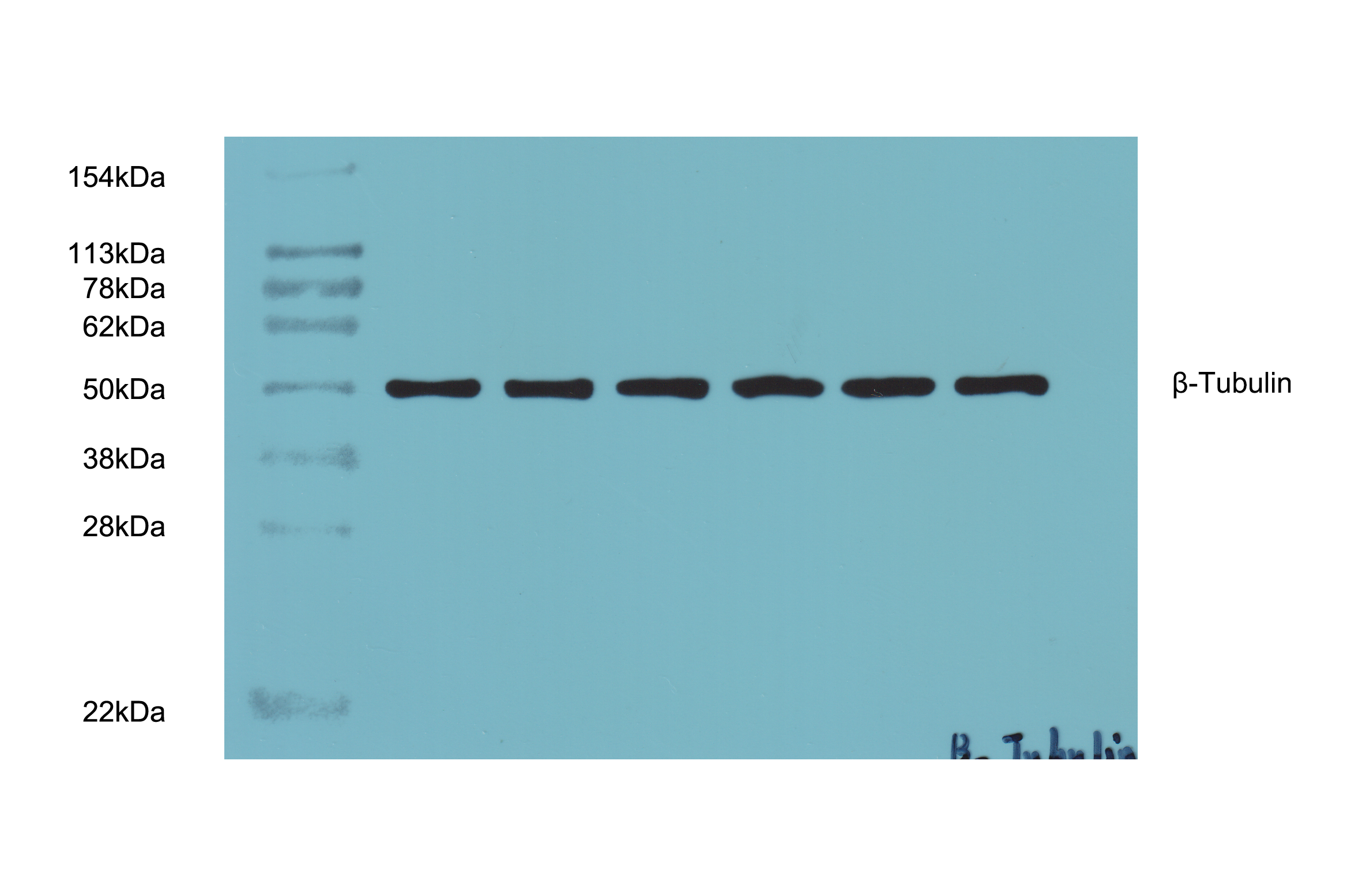

Supplement: Supplementary file 5 [file DataSheet5.zip › Western Blot_original gels 1/1/Western blot_β-Tubulin.tif]

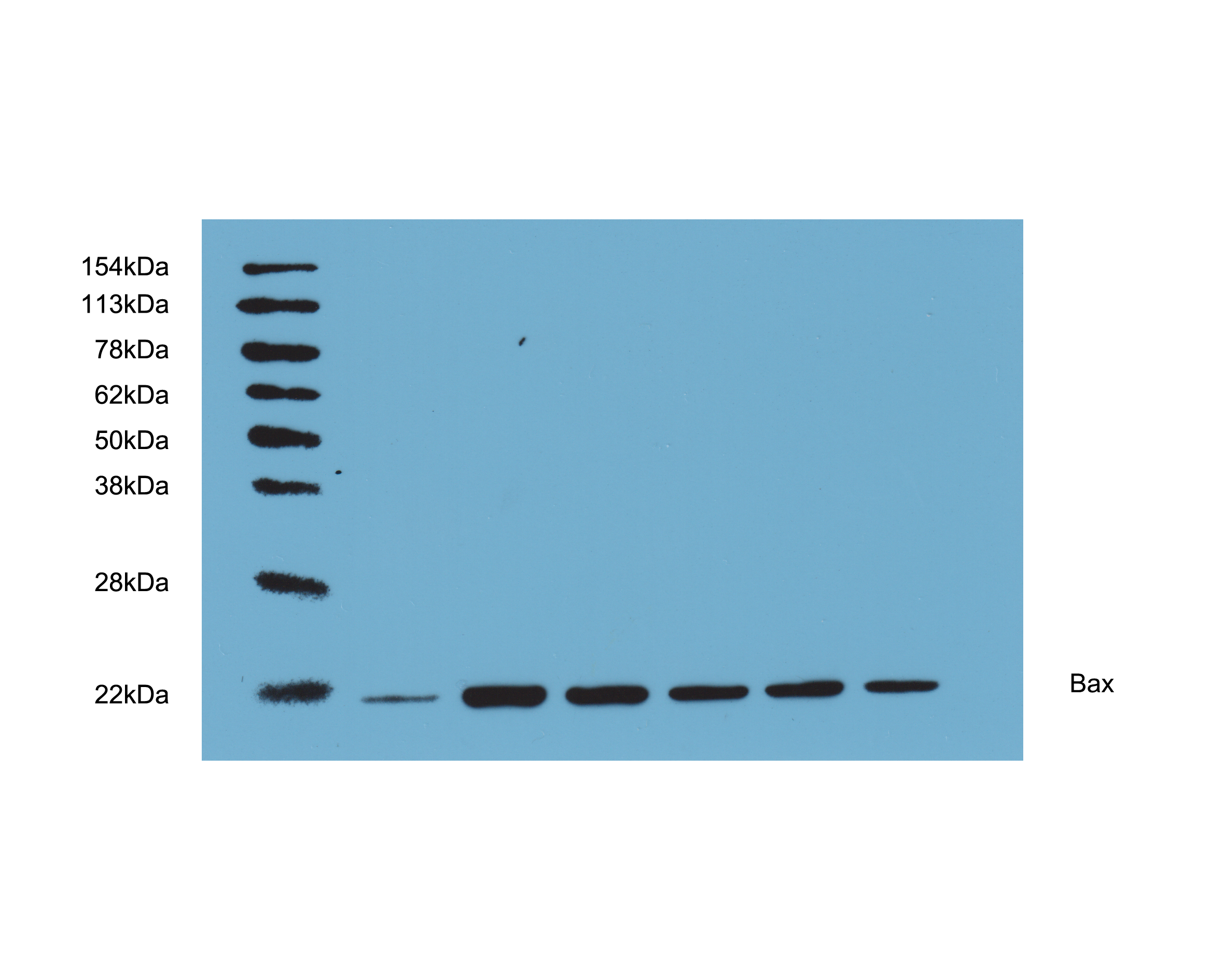

Supplement: Supplementary file 6 [file DataSheet6.zip › Western Blot_original gels 2/2/Western blot_Bax.tif]

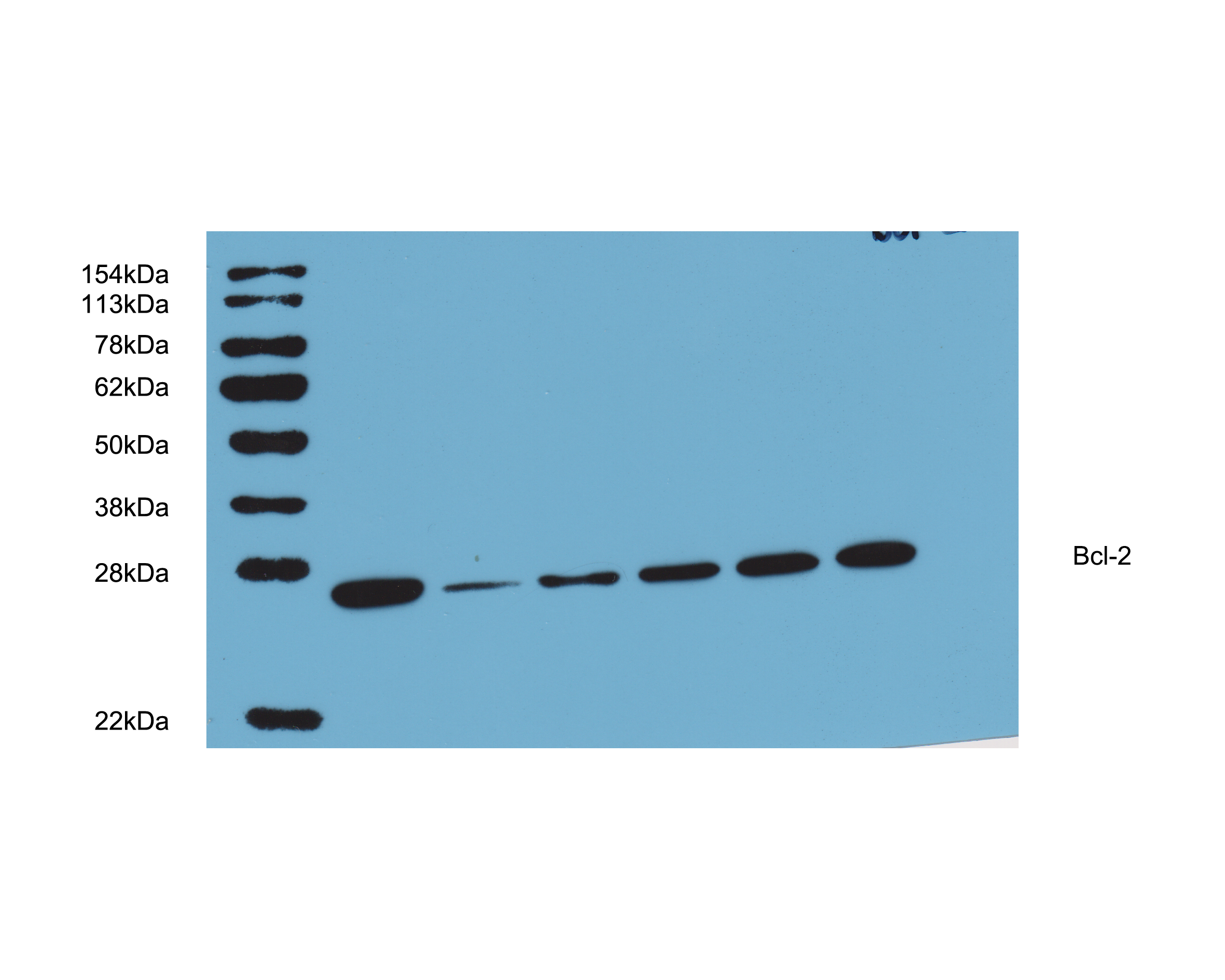

Supplement: Supplementary file 6 [file DataSheet6.zip › Western Blot_original gels 2/2/Western blot_Bcl-2.tif]

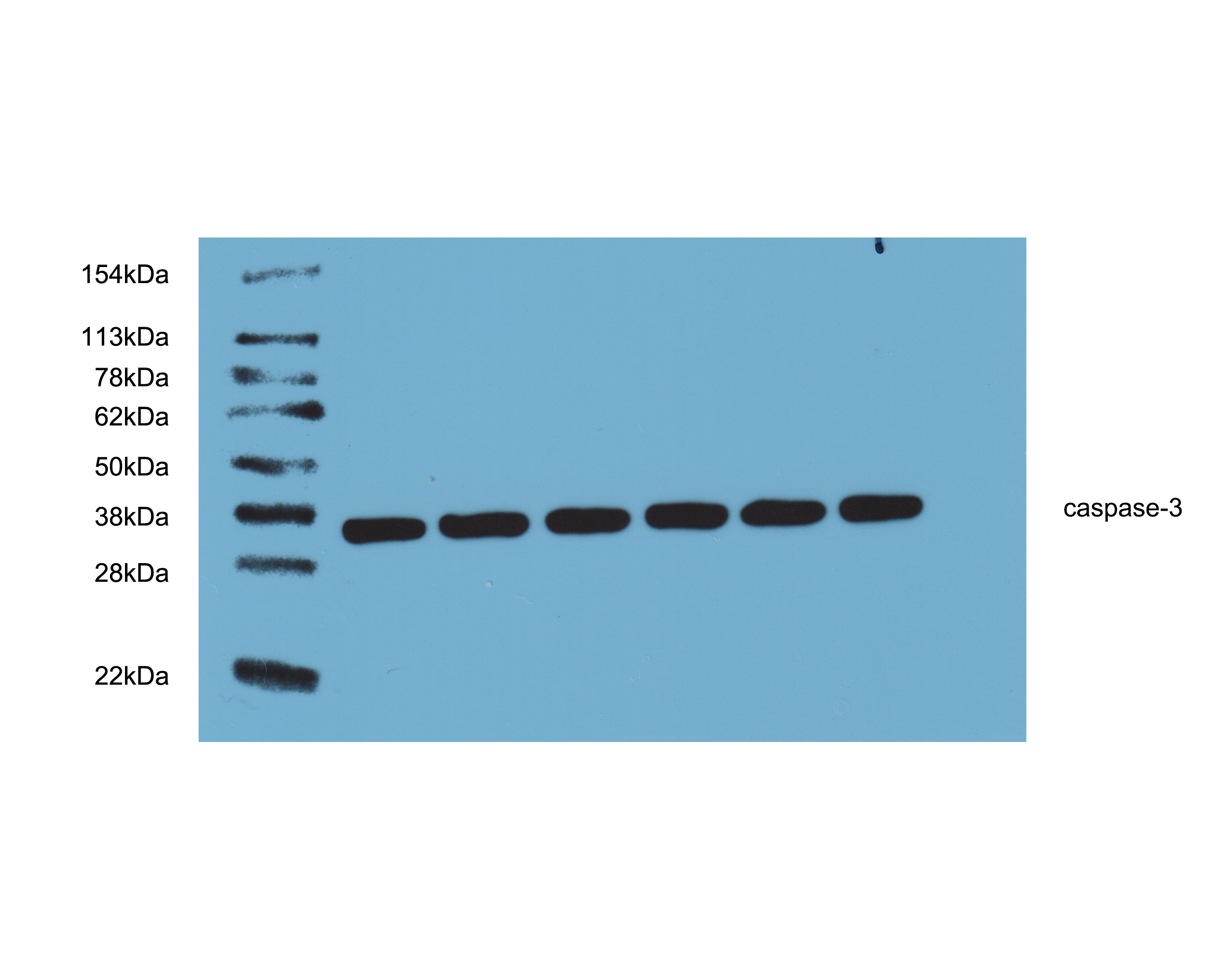

Supplement: Supplementary file 6 [file DataSheet6.zip › Western Blot_original gels 2/2/Western blot_caspase-3.tif]

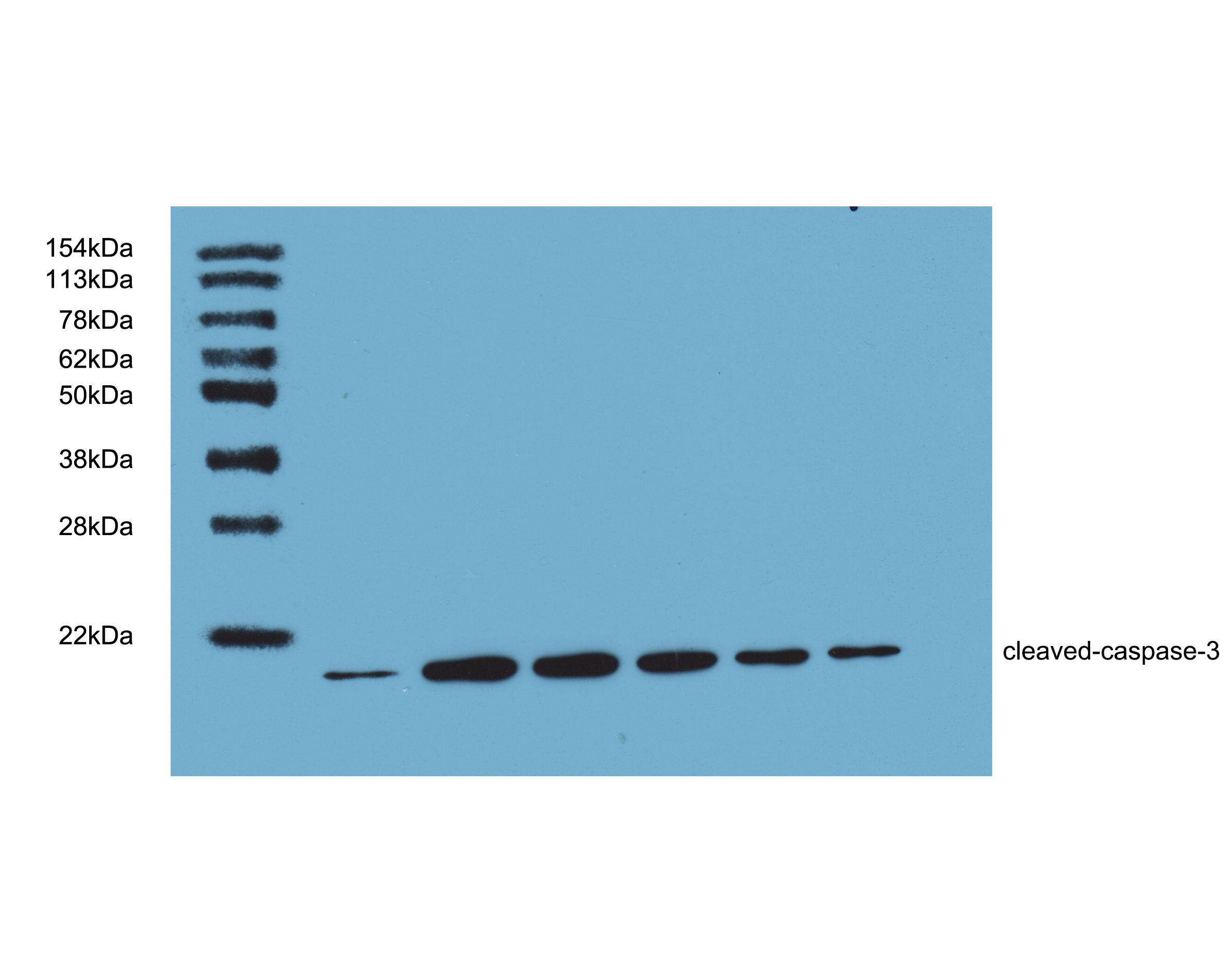

Supplement: Supplementary file 6 [file DataSheet6.zip › Western Blot_original gels 2/2/Western blot_cleaved-caspase-3.tif]

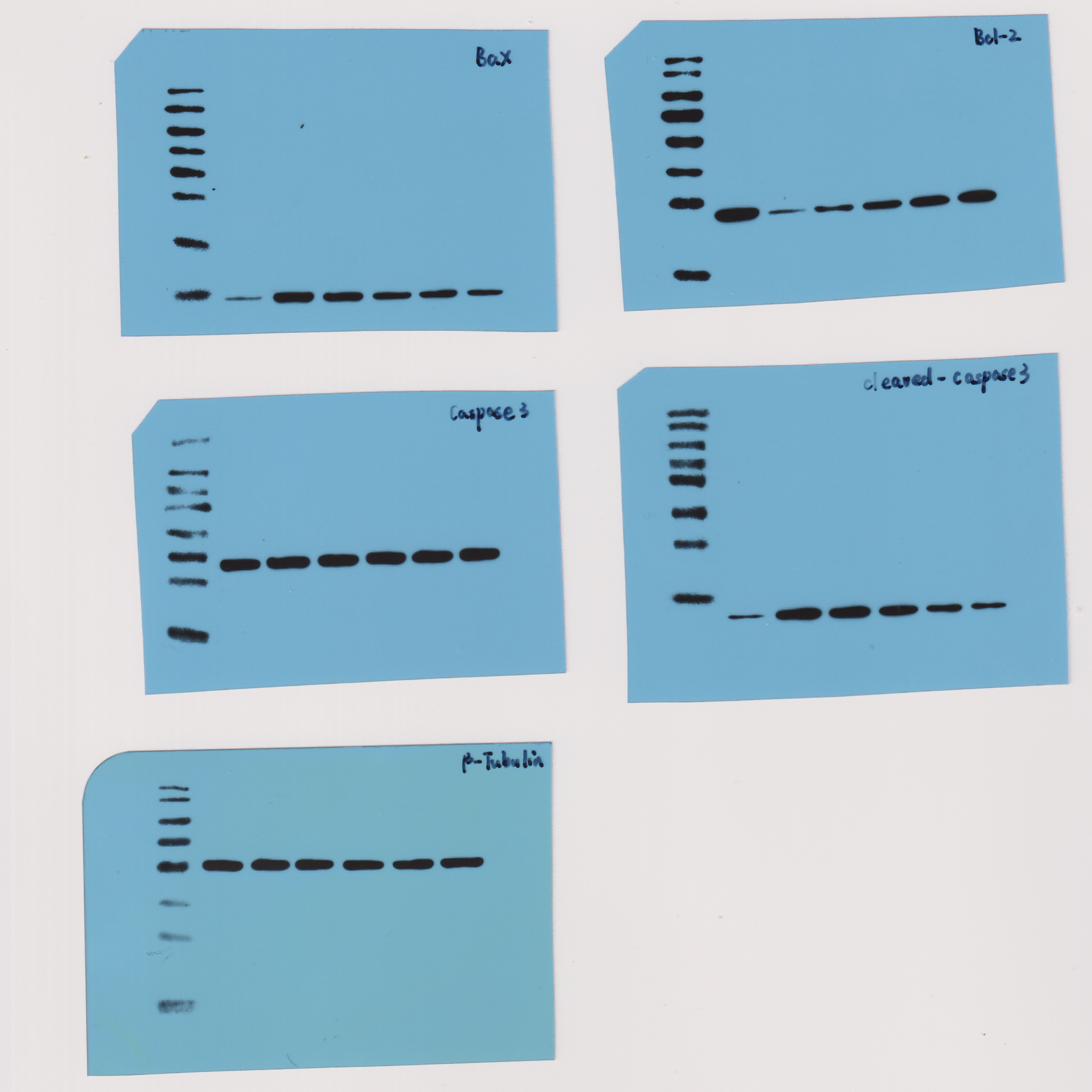

Supplement: Supplementary file 6 [file DataSheet6.zip › Western Blot_original gels 2/2/Western blot_total(1).png]

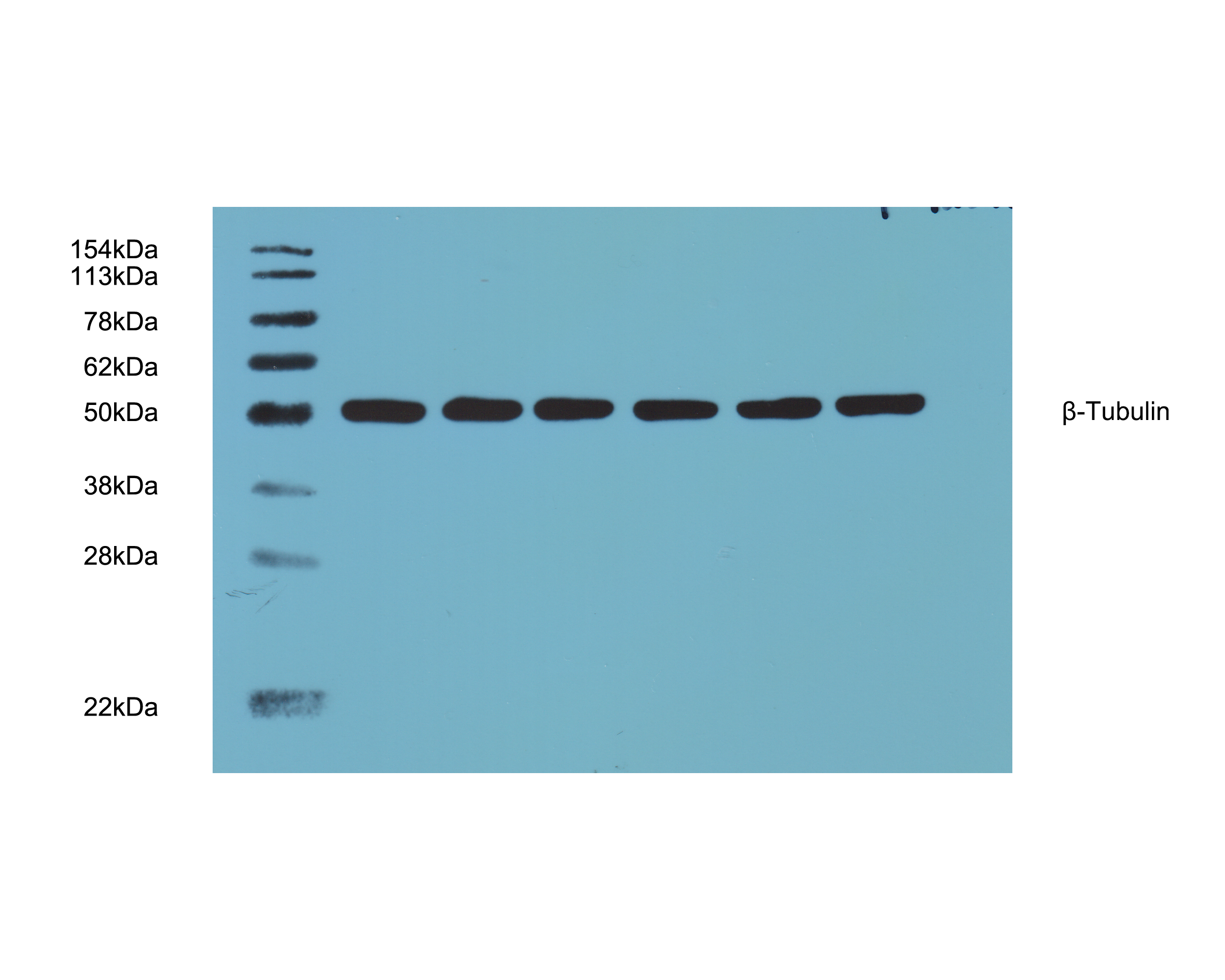

Supplement: Supplementary file 6 [file DataSheet6.zip › Western Blot_original gels 2/2/Western blot_β-Tubulin.tif]

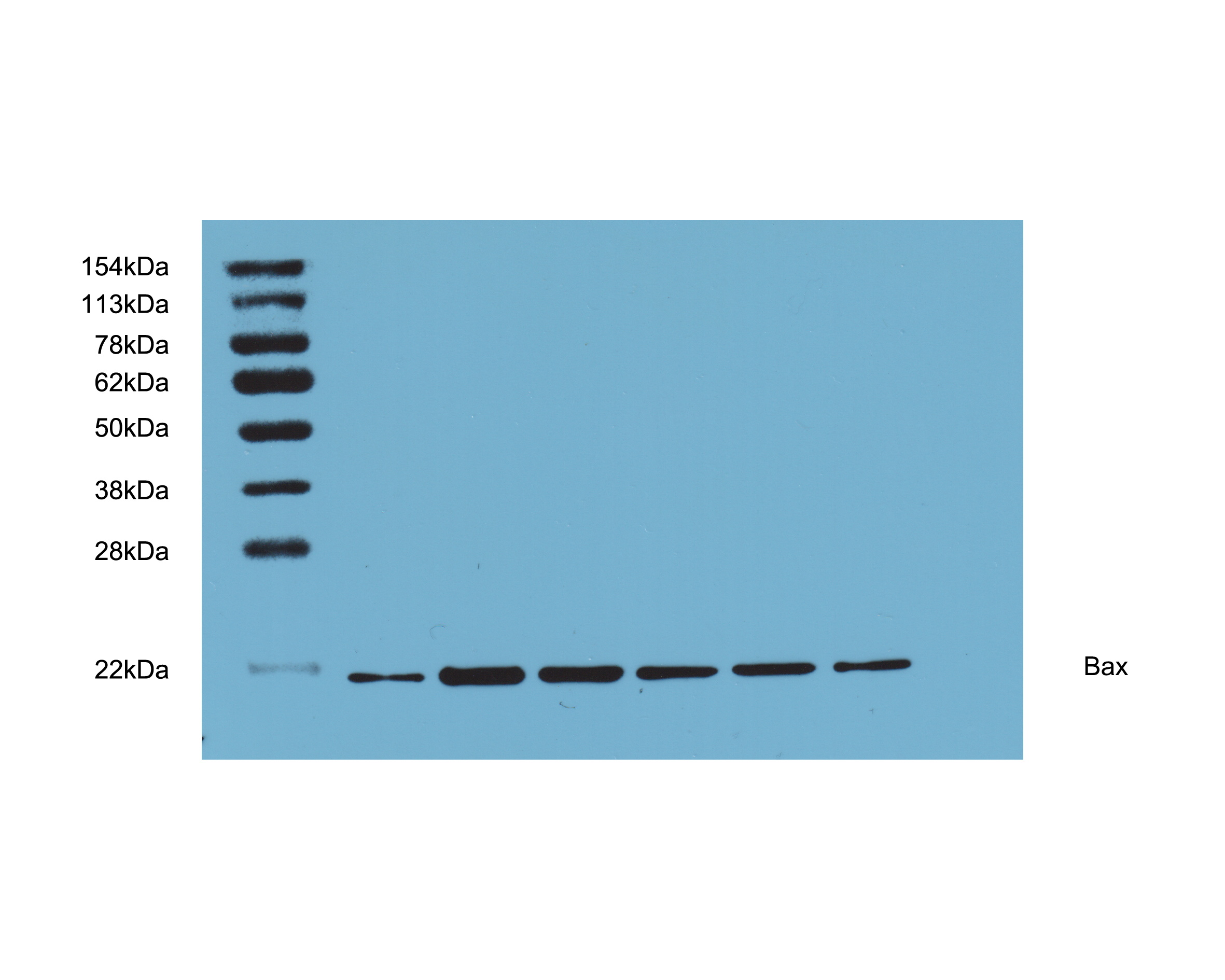

Supplement: Supplementary file 7 [file DataSheet7.zip › Western Blot_original gels 3/3/Western blot_Bax.tif]

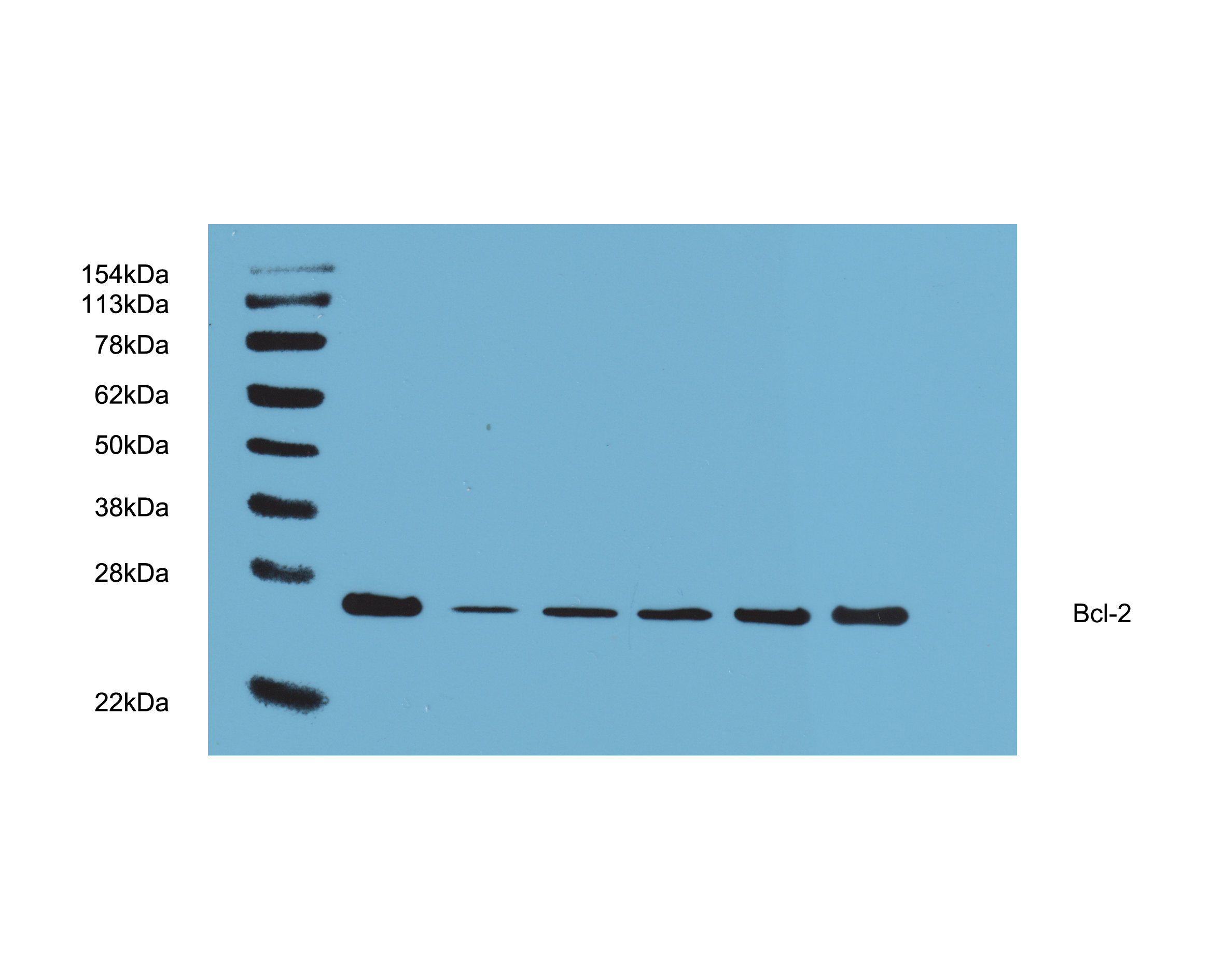

Supplement: Supplementary file 7 [file DataSheet7.zip › Western Blot_original gels 3/3/Western blot_Bcl-2.tif]

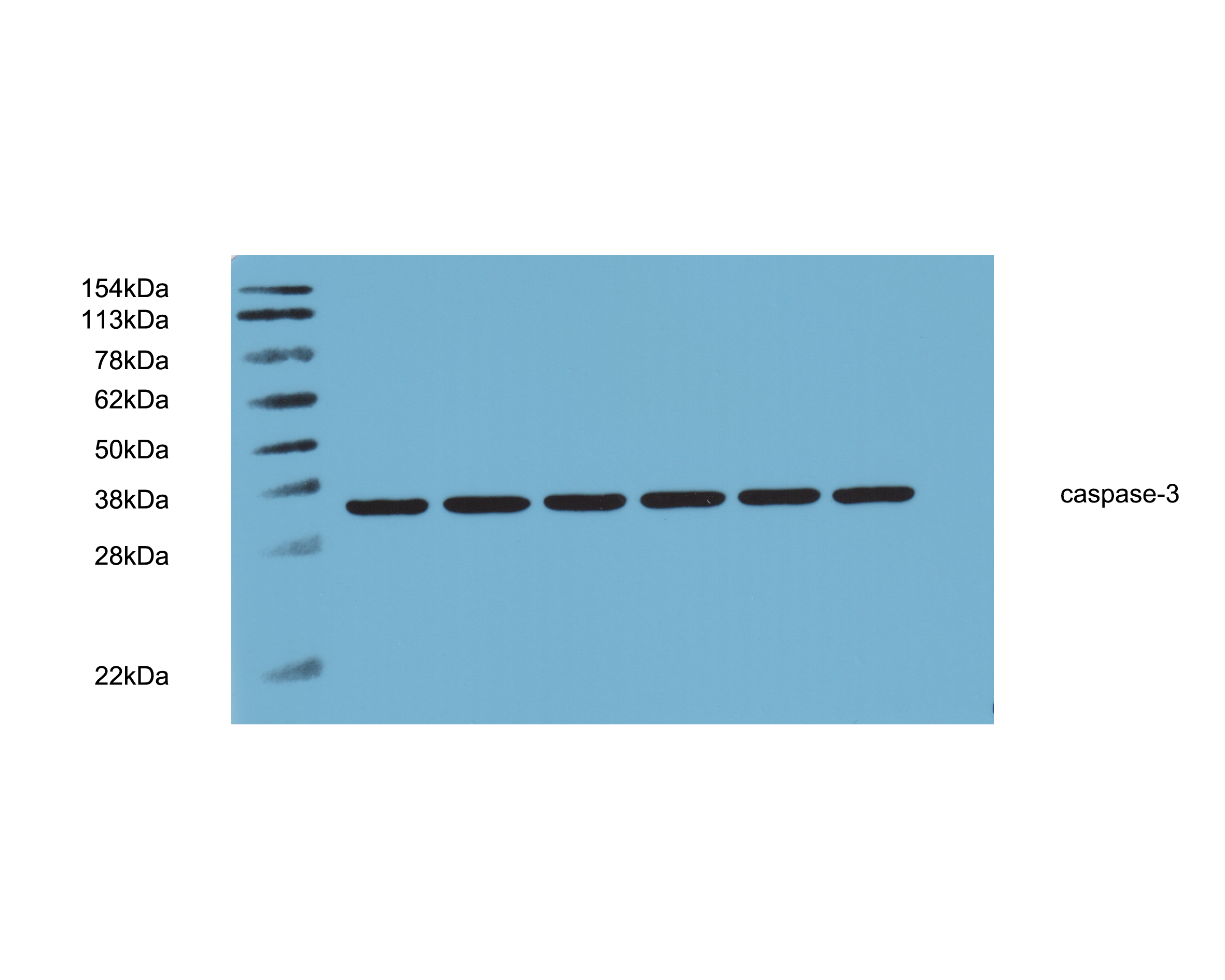

Supplement: Supplementary file 7 [file DataSheet7.zip › Western Blot_original gels 3/3/Western blot_caspase-3.tif]

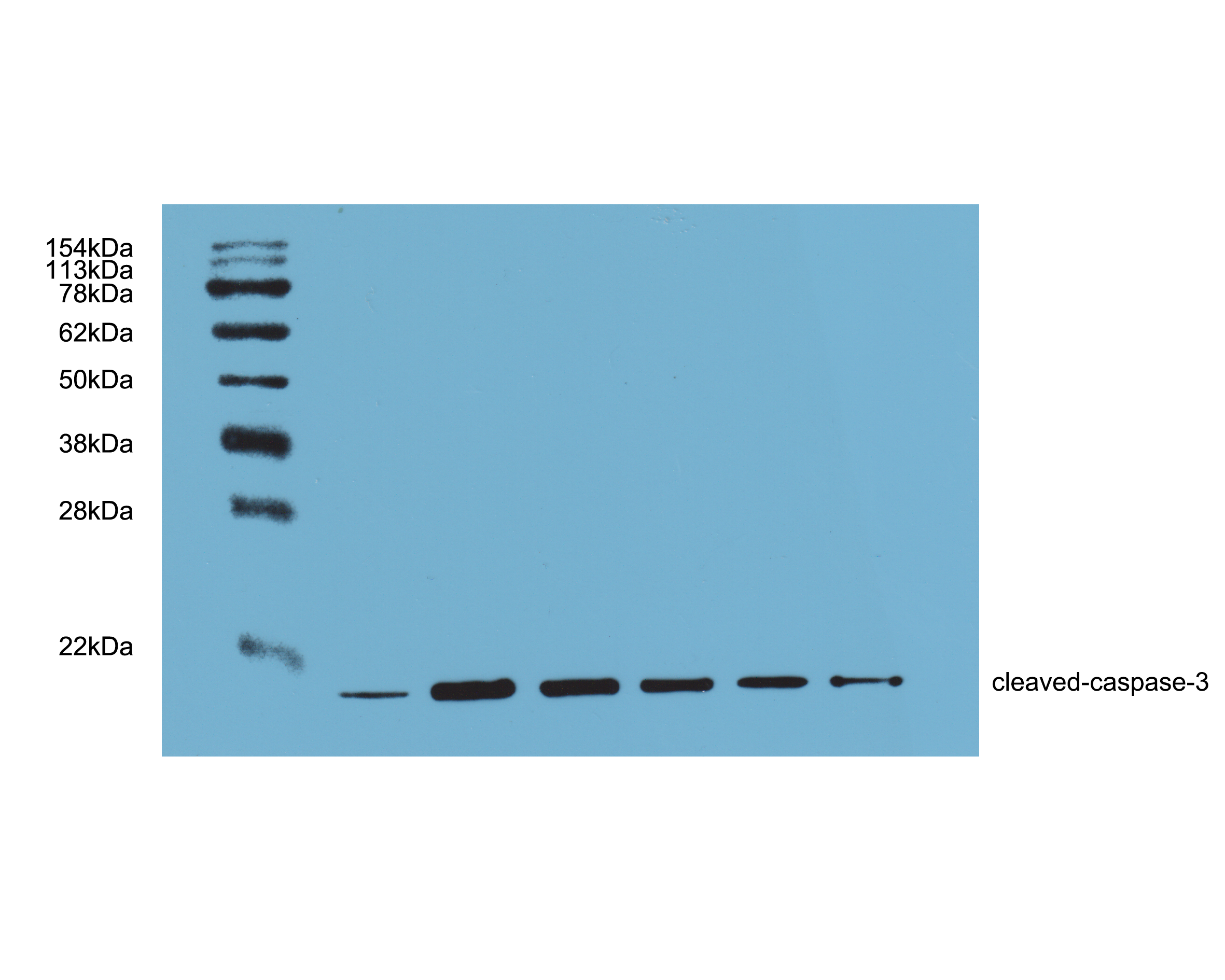

Supplement: Supplementary file 7 [file DataSheet7.zip › Western Blot_original gels 3/3/Western blot_cleaved-caspase-3.tif]

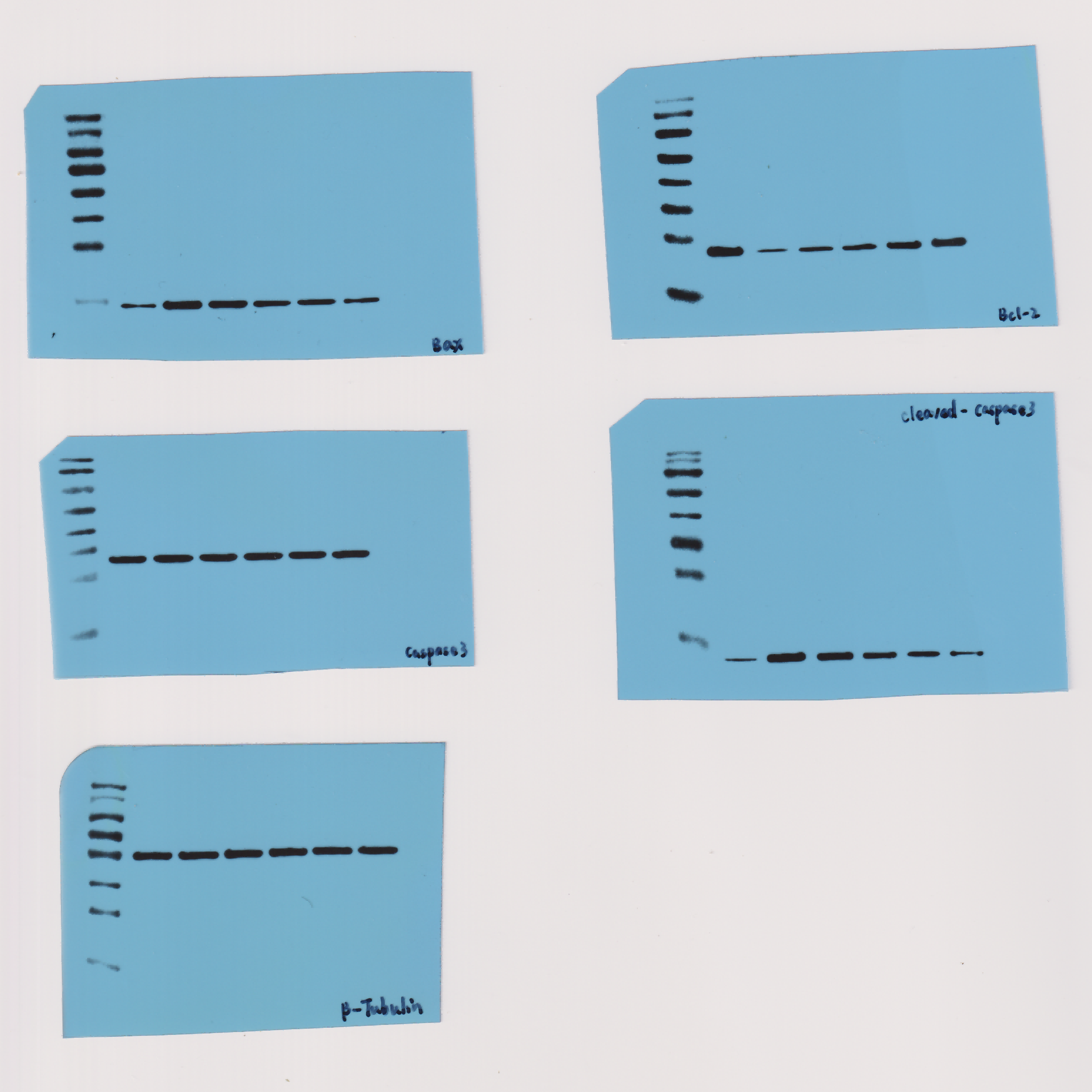

Supplement: Supplementary file 7 [file DataSheet7.zip › Western Blot_original gels 3/3/Western blot_total(1).png]

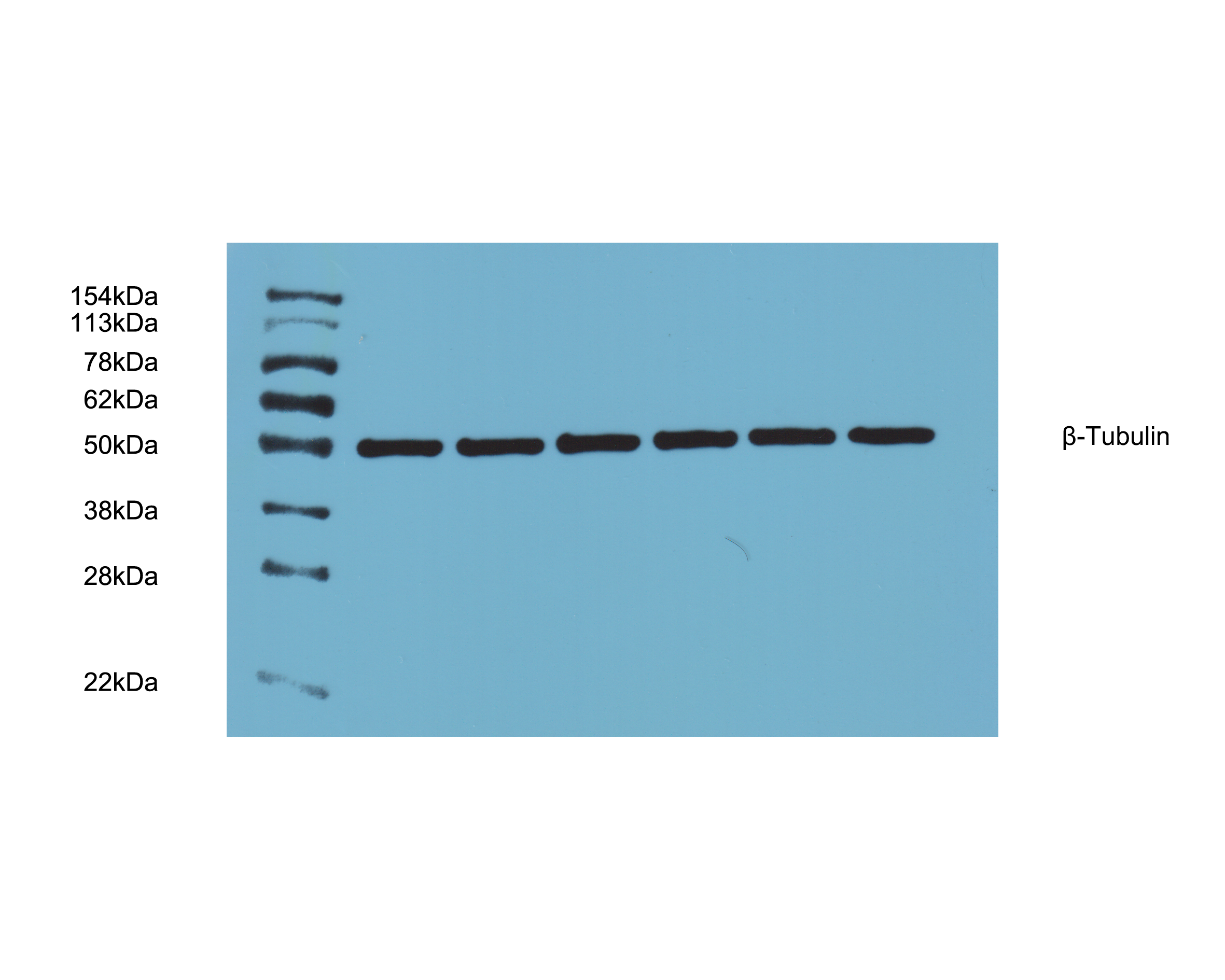

Supplement: Supplementary file 7 [file DataSheet7.zip › Western Blot_original gels 3/3/Western blot_β-Tubulin.tif]

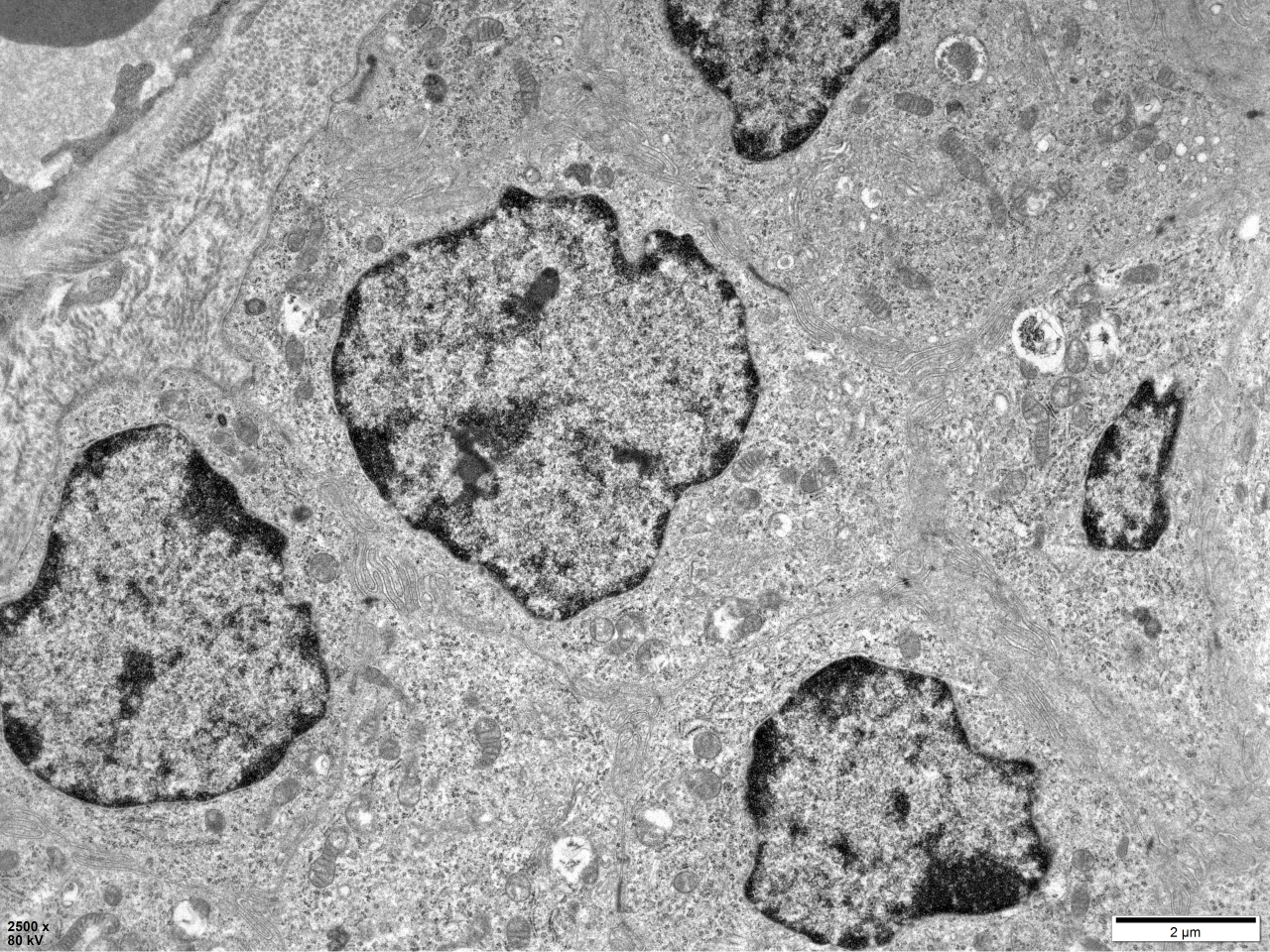

Supplement: Supplementary file 12 [file DataSheet12.zip › Electron Microscopy images/A20243138_0001(1).png]

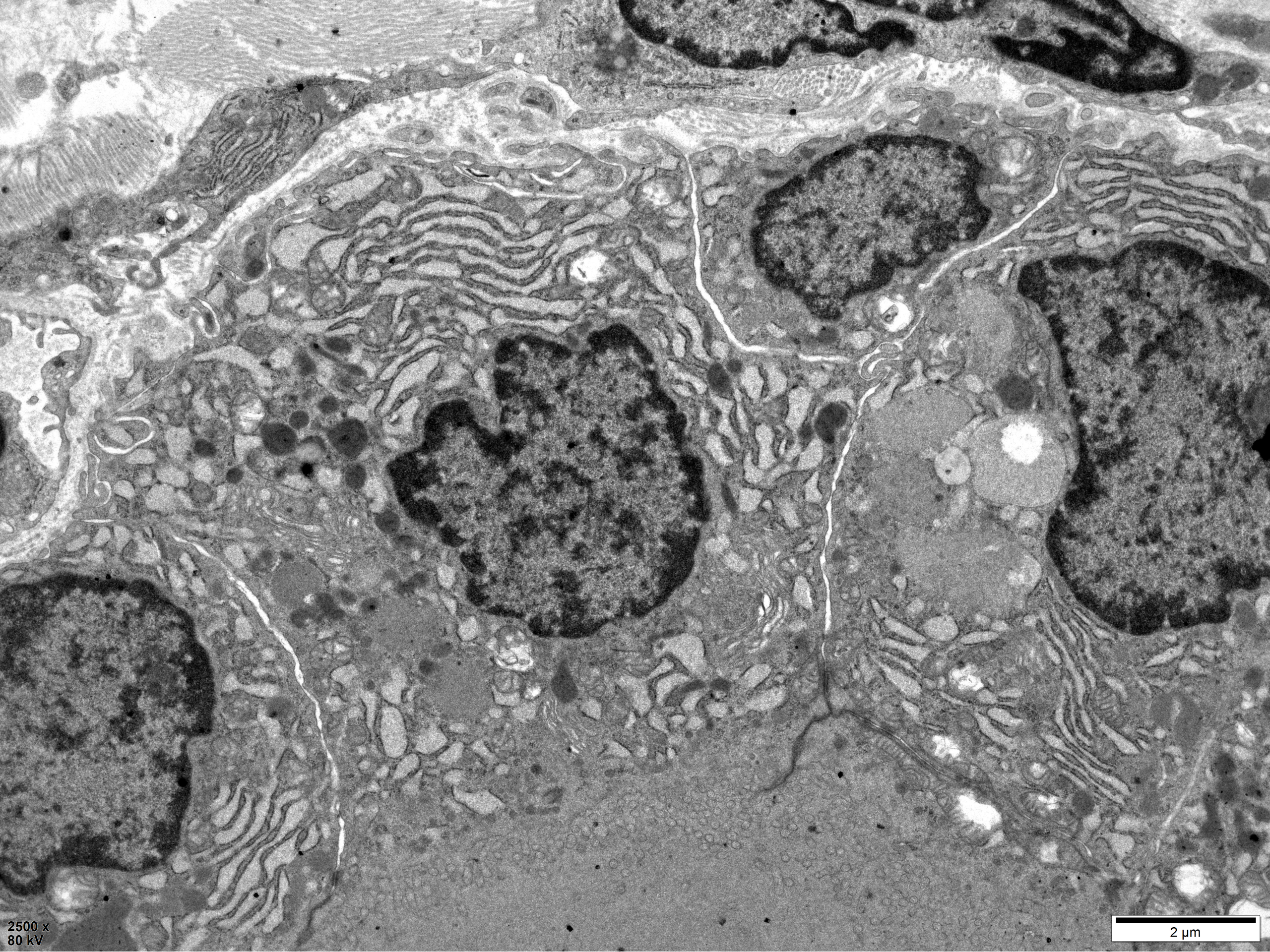

Supplement: Supplementary file 12 [file DataSheet12.zip › Electron Microscopy images/C20241611_0004(1).png]

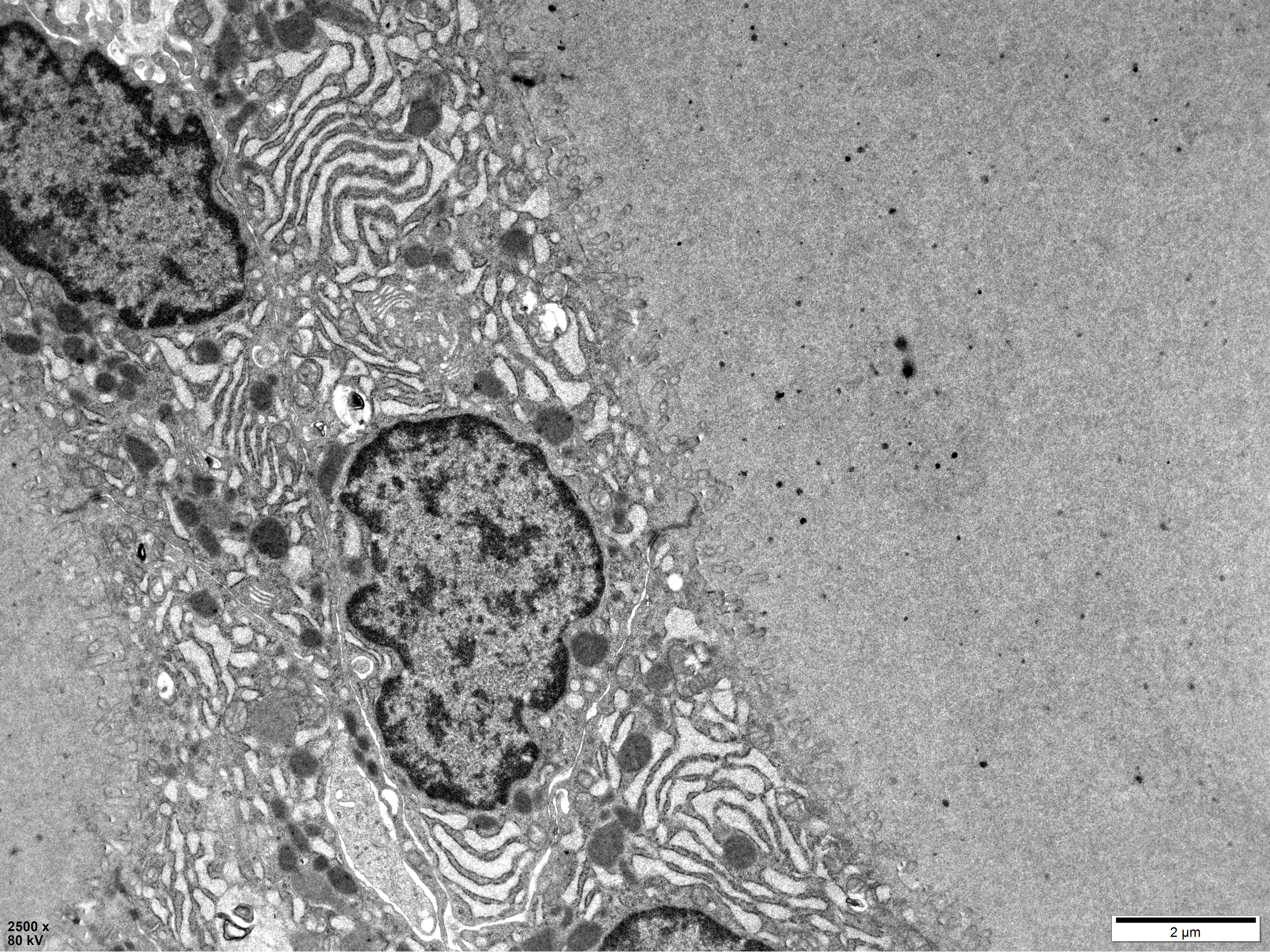

Supplement: Supplementary file 12 [file DataSheet12.zip › Electron Microscopy images/M20241612_0004(1).png]

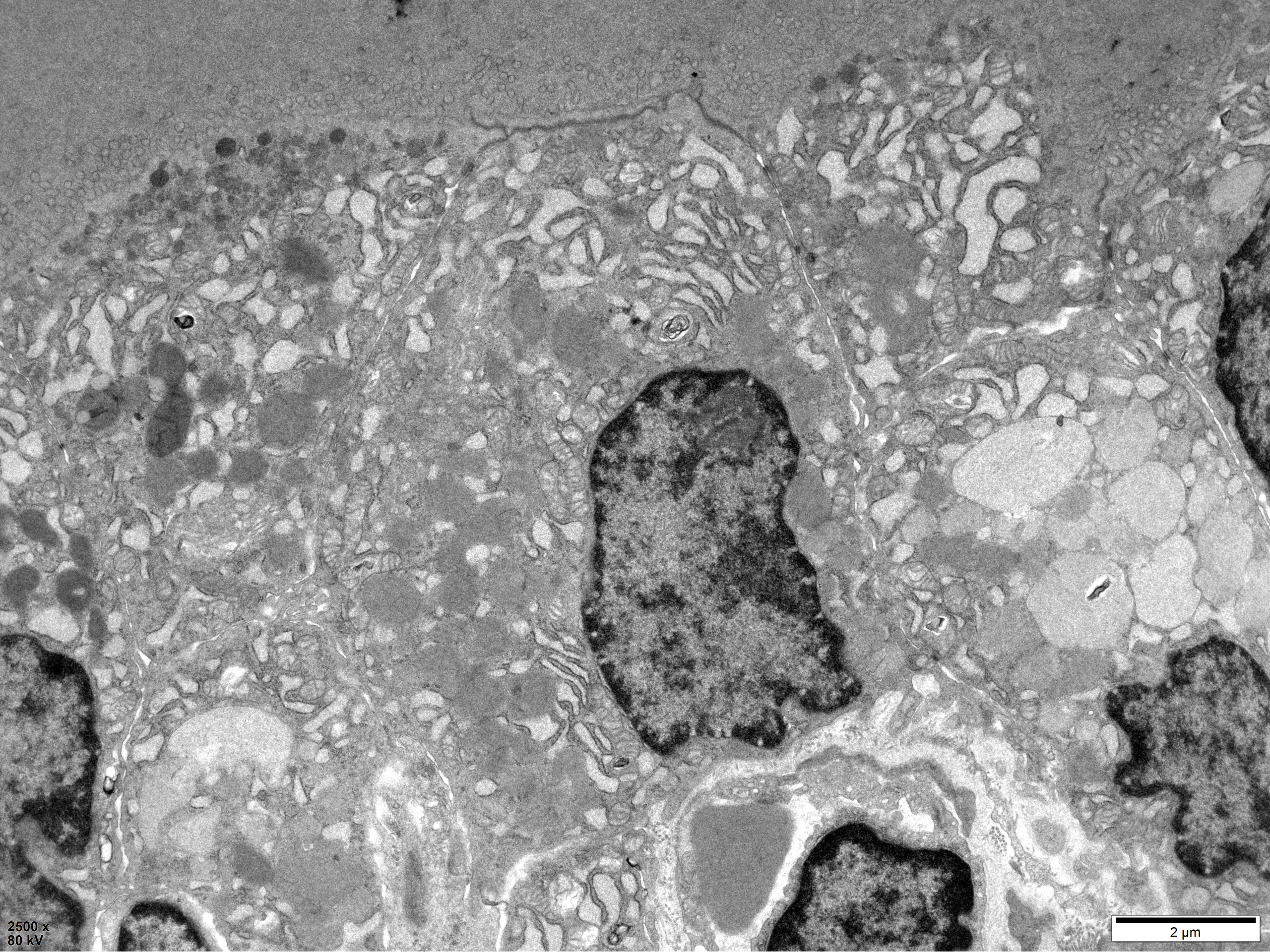

Supplement: Supplementary file 12 [file DataSheet12.zip › Electron Microscopy images/S20243144- (1)(1).png]

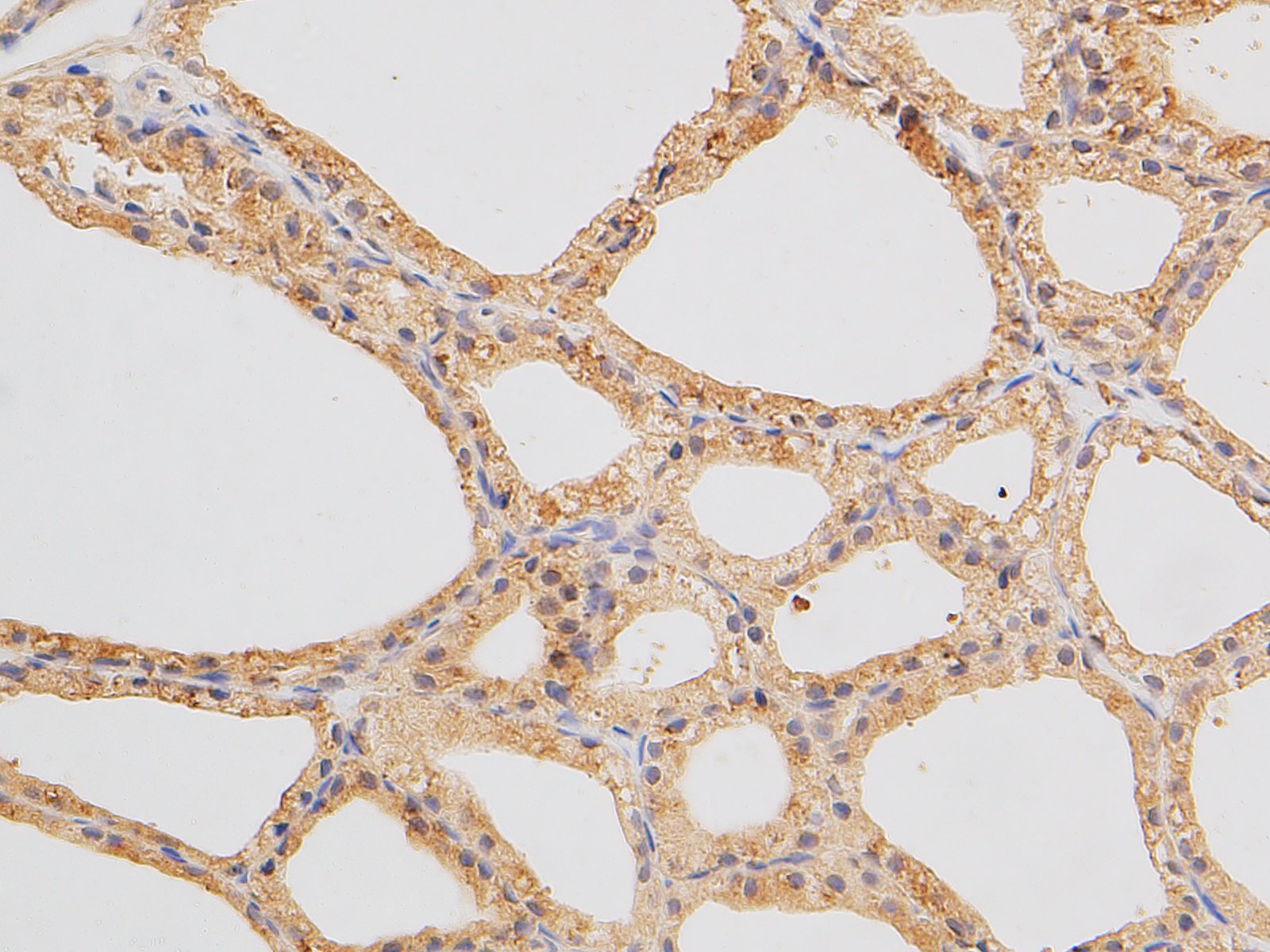

Supplement: Supplementary file 13 [file DataSheet13.zip › immunocytochemistry images/A1-400 (1).jpg]

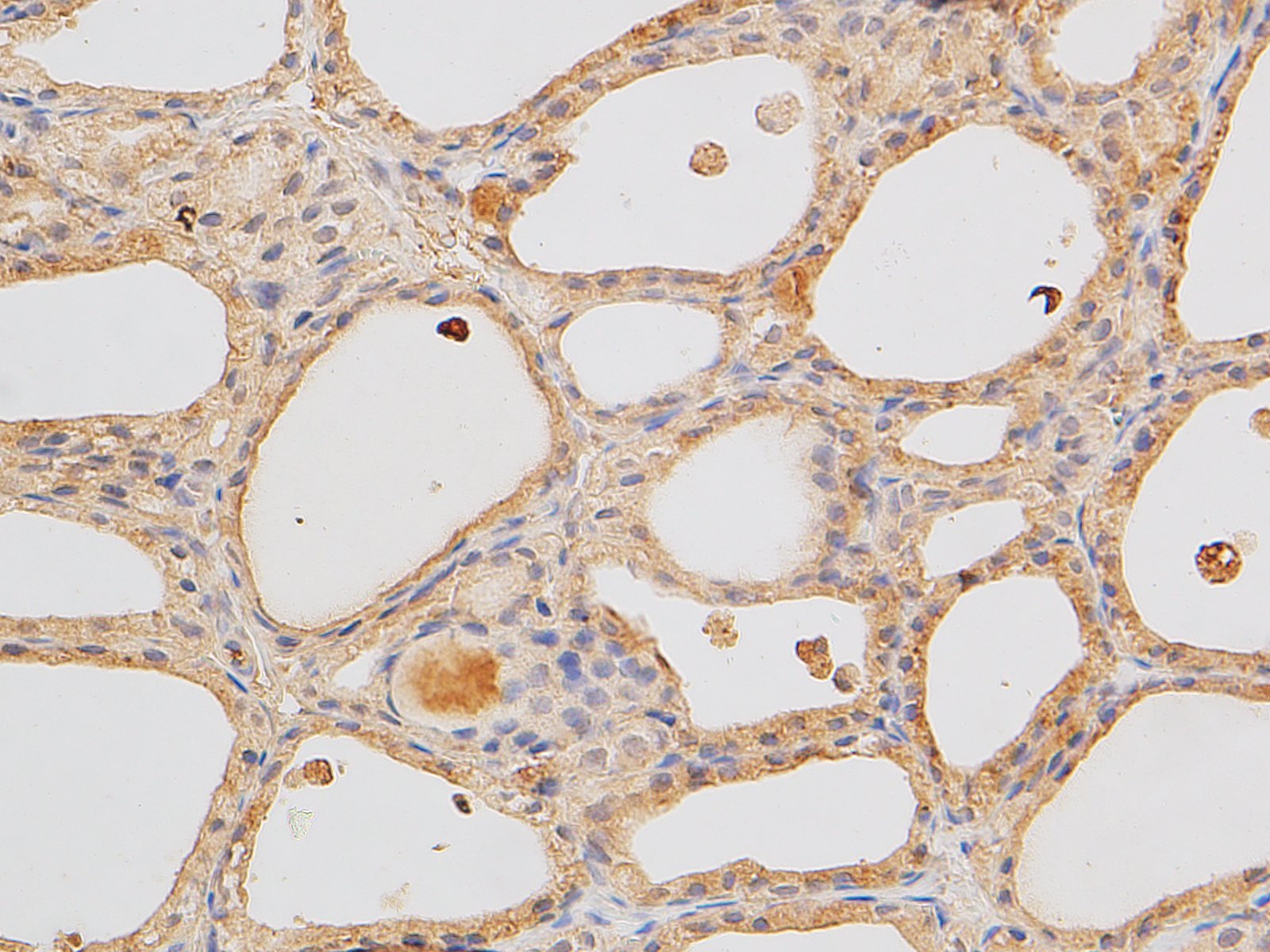

Supplement: Supplementary file 13 [file DataSheet13.zip › immunocytochemistry images/A1-400 (2).jpg]

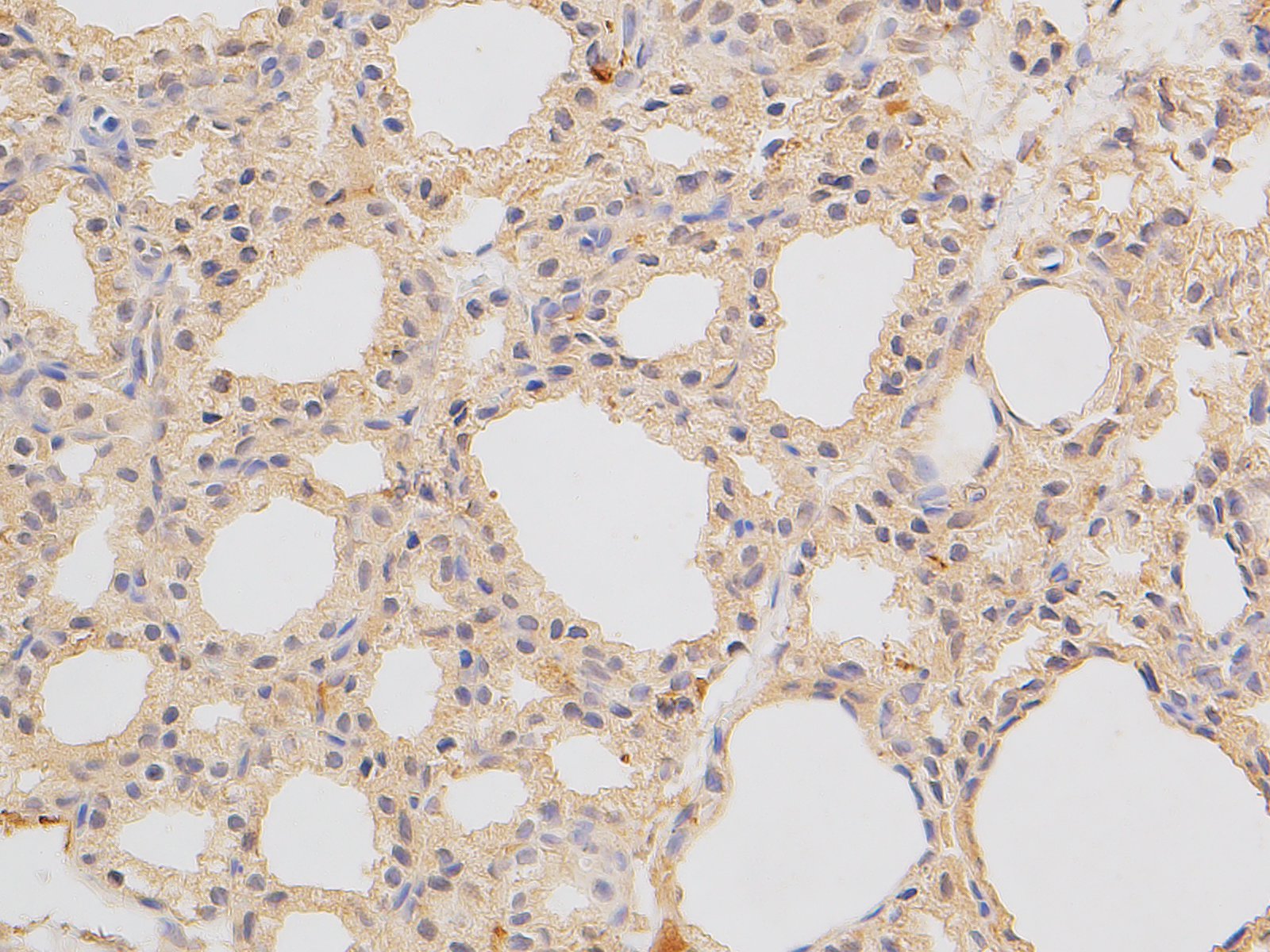

Supplement: Supplementary file 13 [file DataSheet13.zip › immunocytochemistry images/A2-400 (1).jpg]

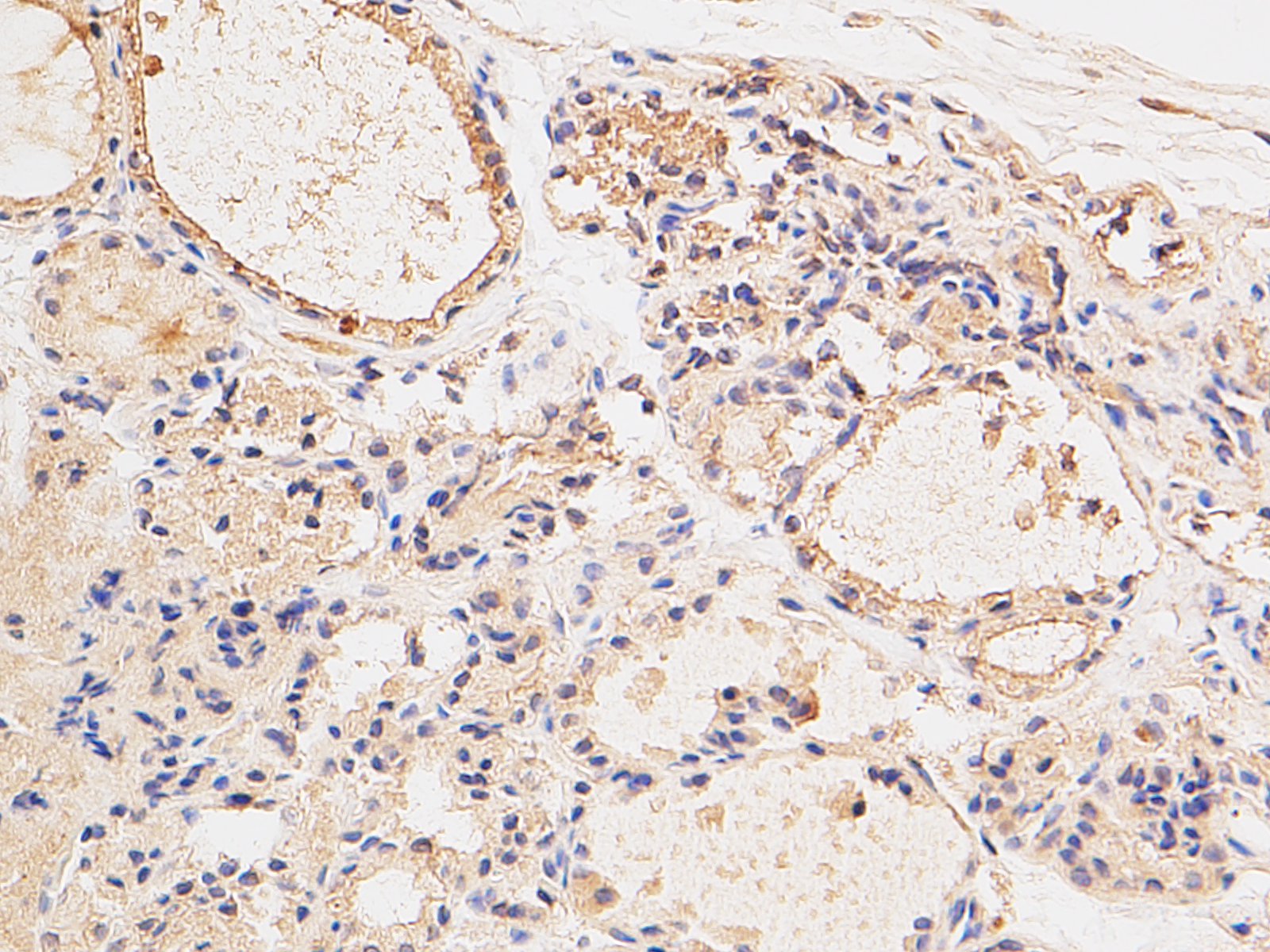

Supplement: Supplementary file 13 [file DataSheet13.zip › immunocytochemistry images/A2-400 (2).jpg]

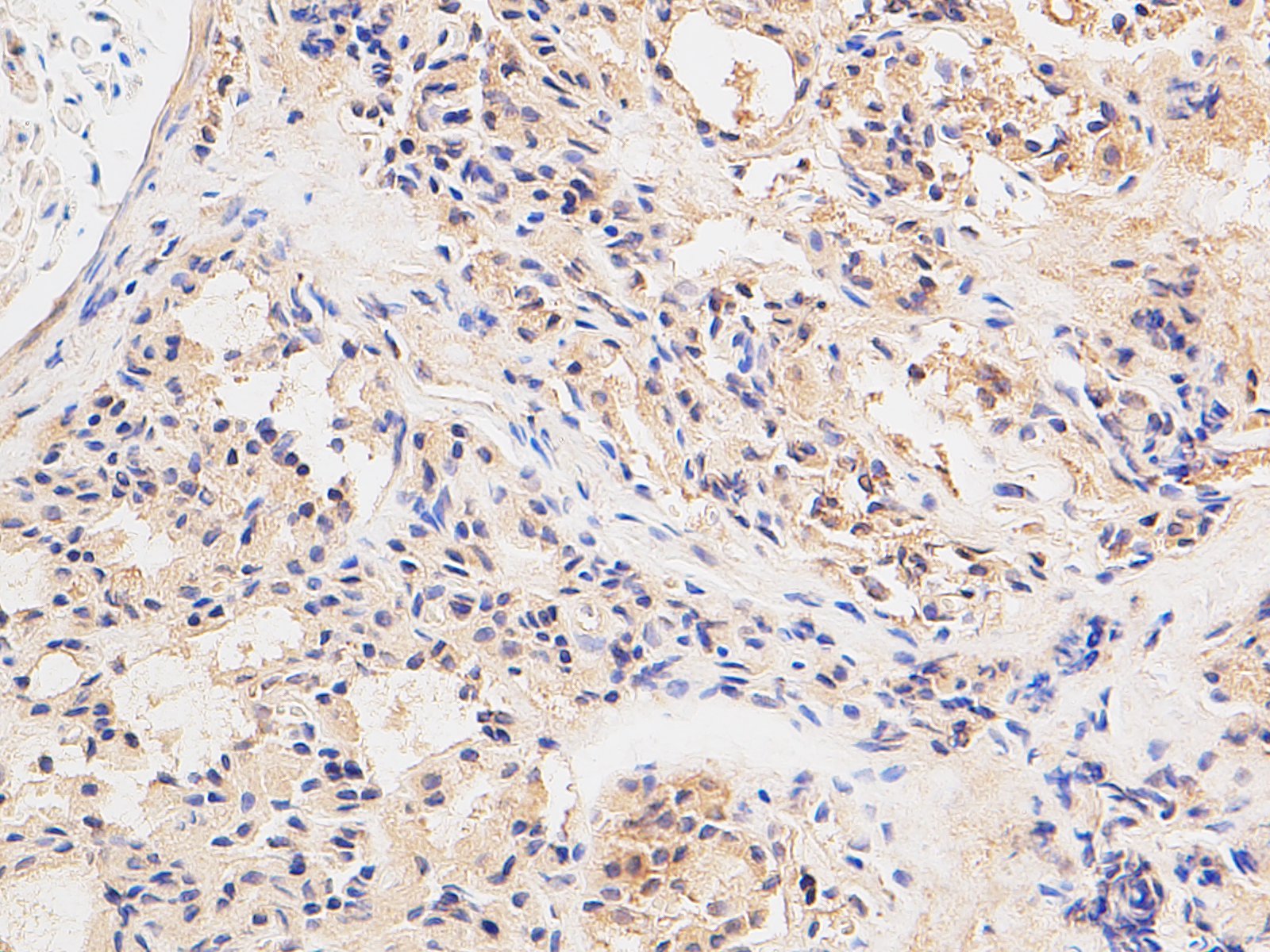

Supplement: Supplementary file 13 [file DataSheet13.zip › immunocytochemistry images/A3 400 (2).jpg]

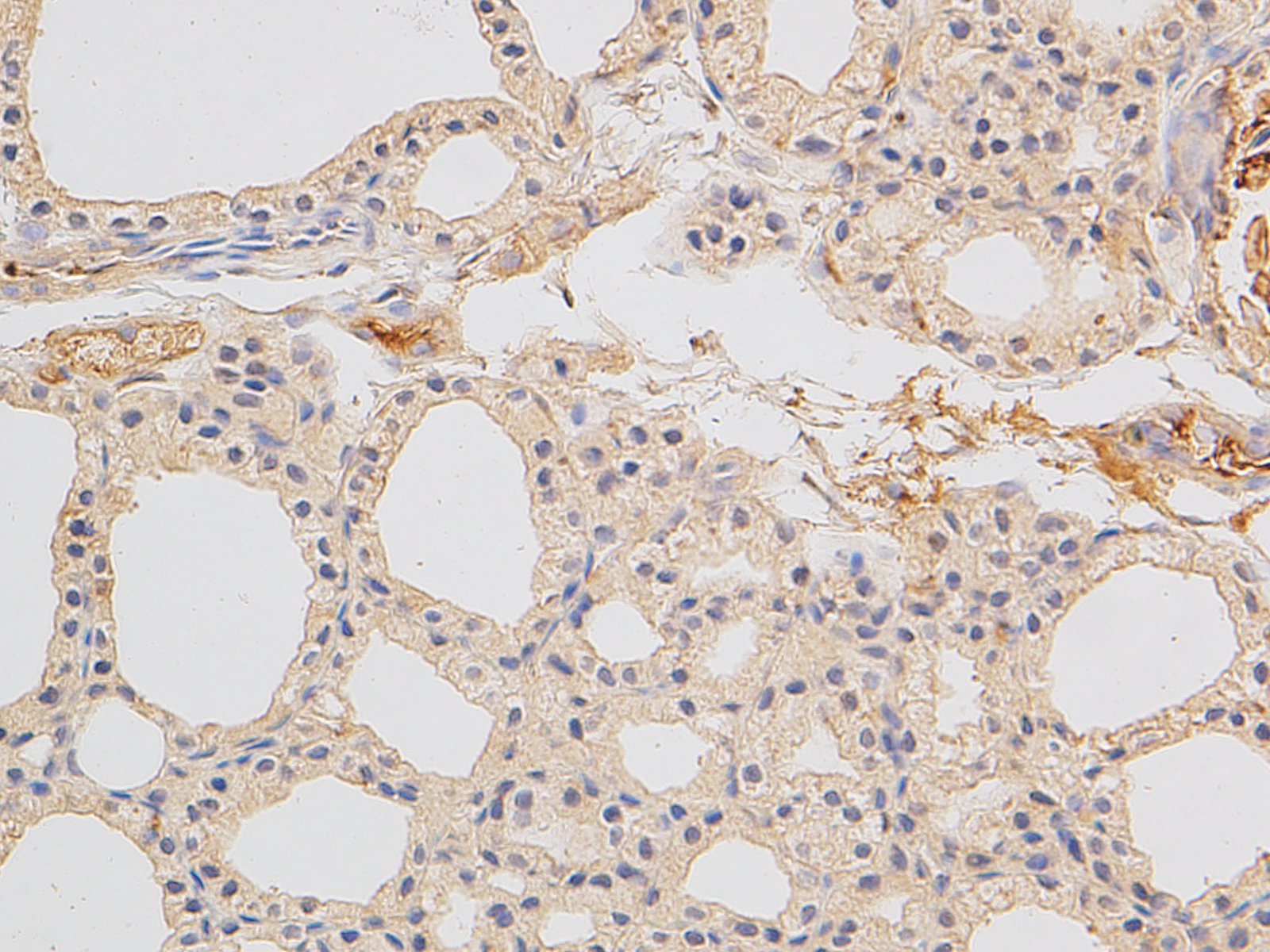

Supplement: Supplementary file 13 [file DataSheet13.zip › immunocytochemistry images/A3-400 (2).jpg]

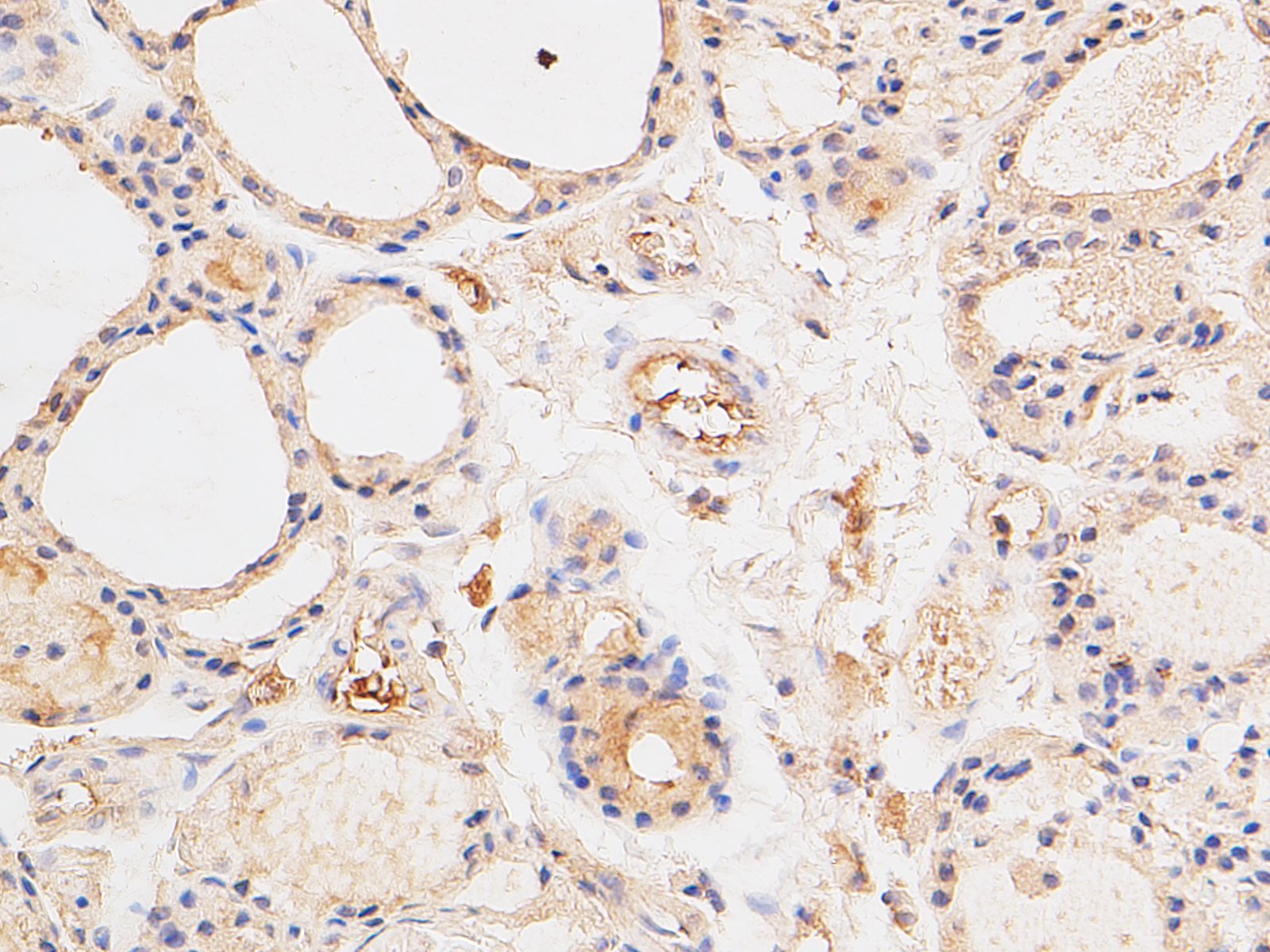

Supplement: Supplementary file 13 [file DataSheet13.zip › immunocytochemistry images/C1- 400 (2).jpg]

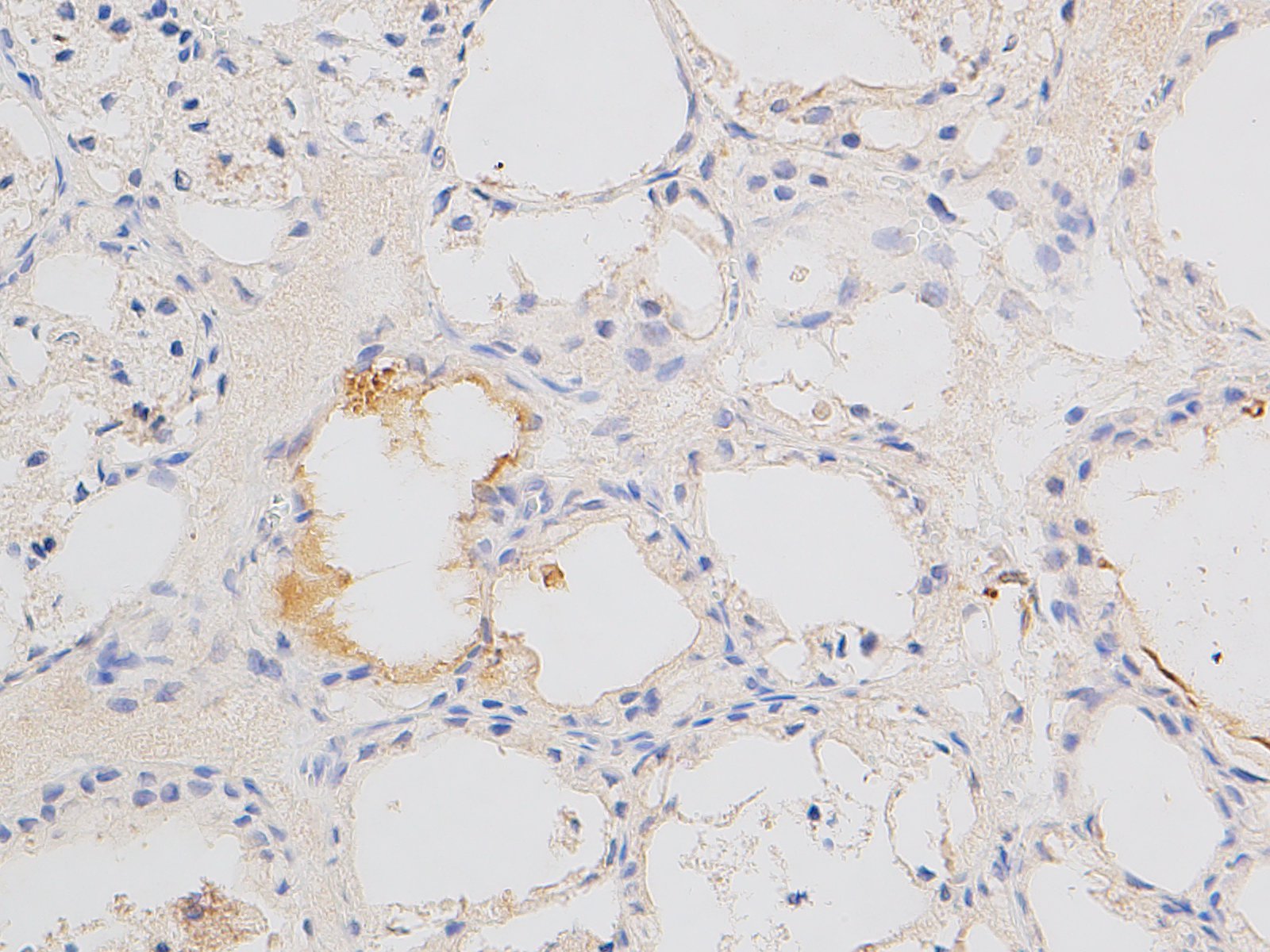

Supplement: Supplementary file 13 [file DataSheet13.zip › immunocytochemistry images/C1-400 (1).jpg]

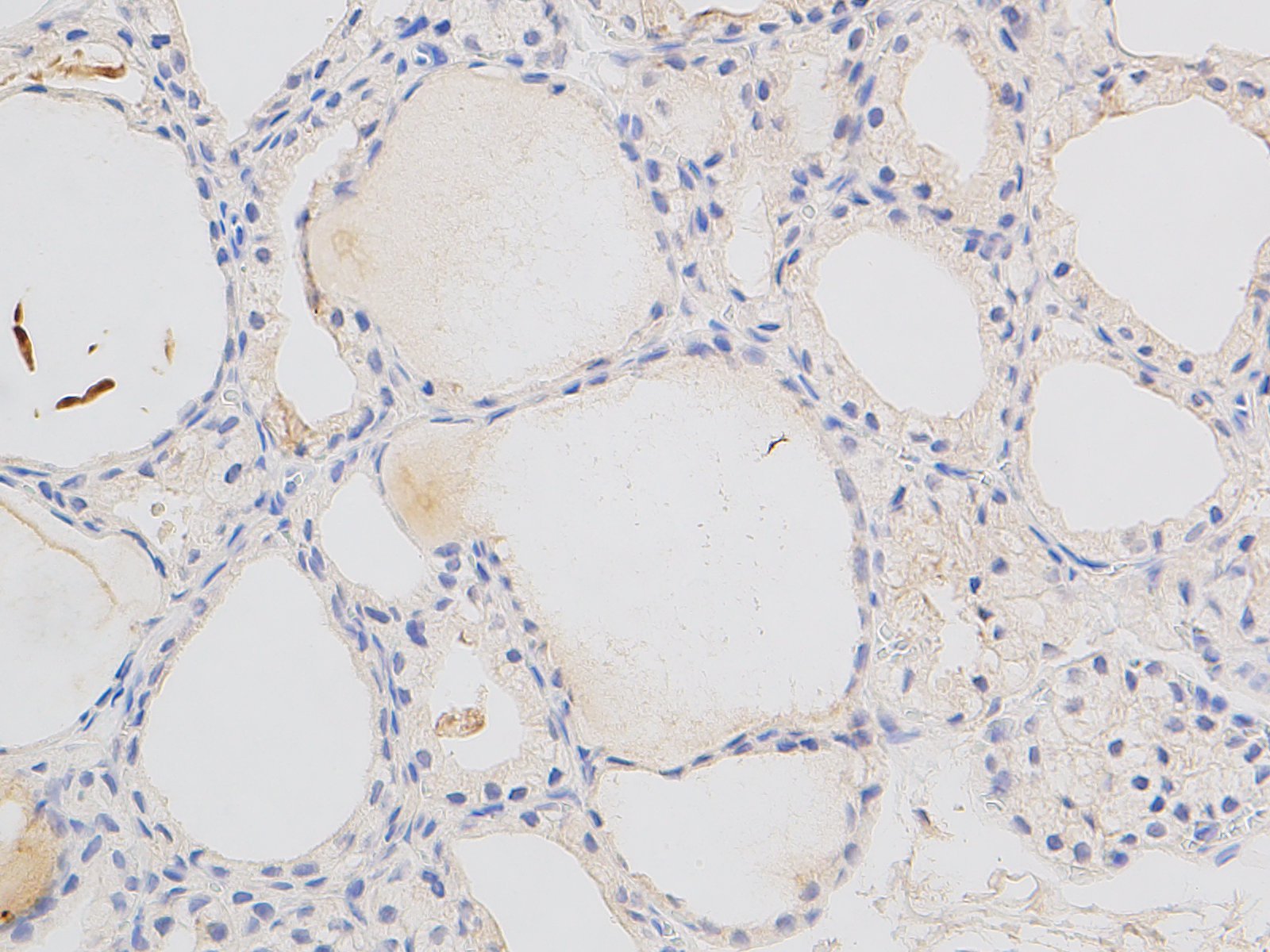

Supplement: Supplementary file 13 [file DataSheet13.zip › immunocytochemistry images/C2-400 (1).jpg]

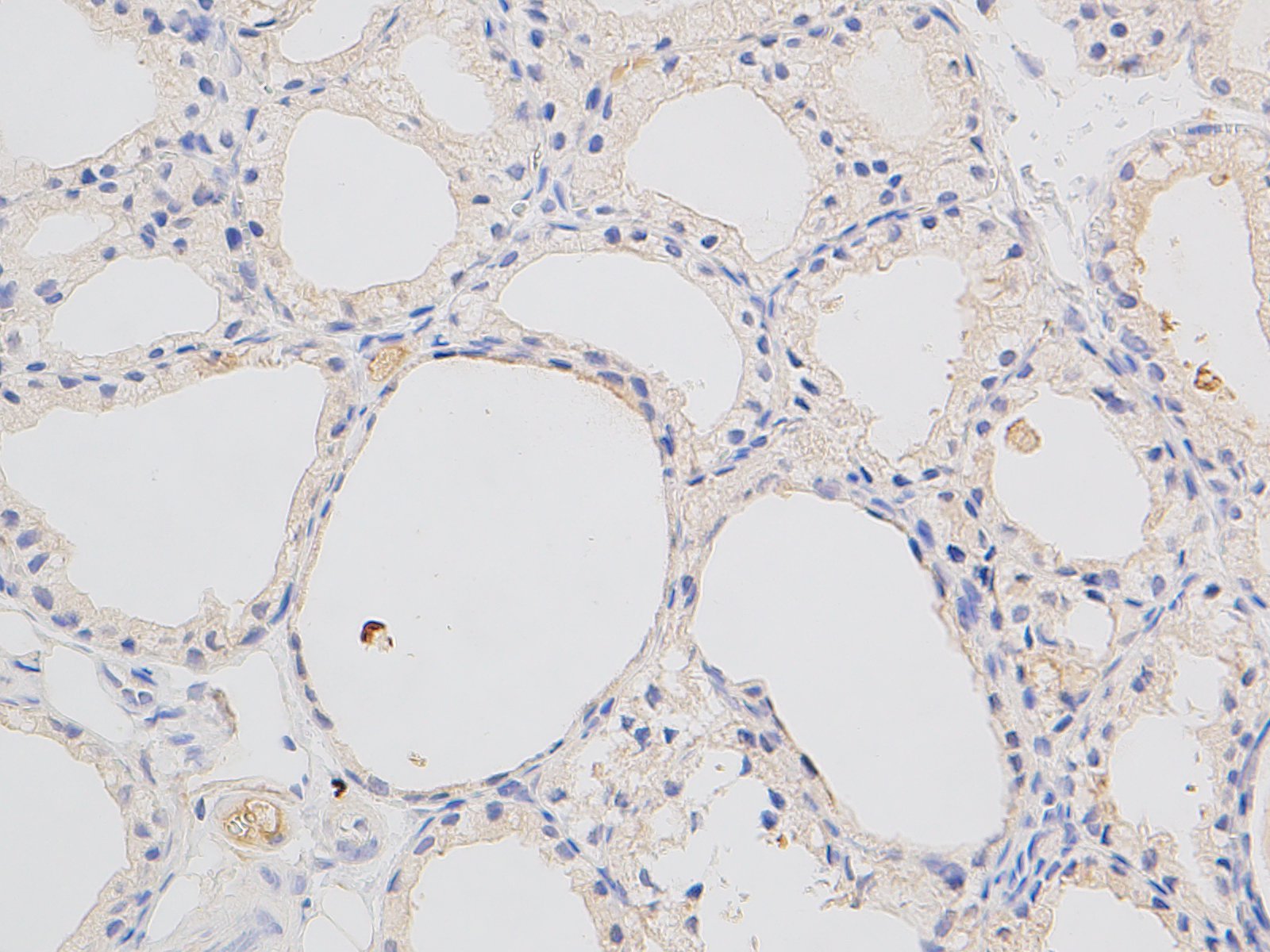

Supplement: Supplementary file 13 [file DataSheet13.zip › immunocytochemistry images/C2-400 (2).jpg]

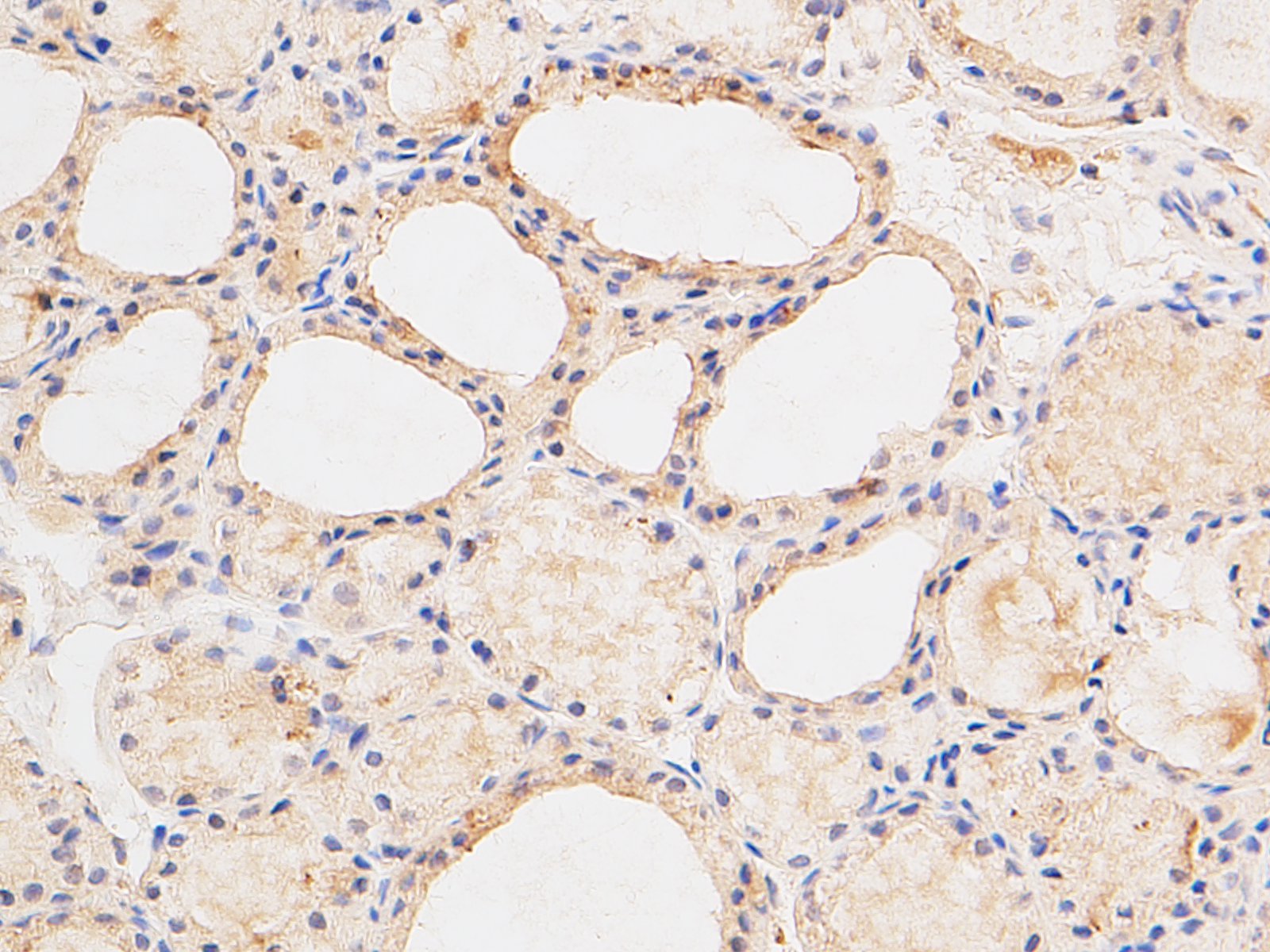

Supplement: Supplementary file 13 [file DataSheet13.zip › immunocytochemistry images/C3-400 (1).jpg]

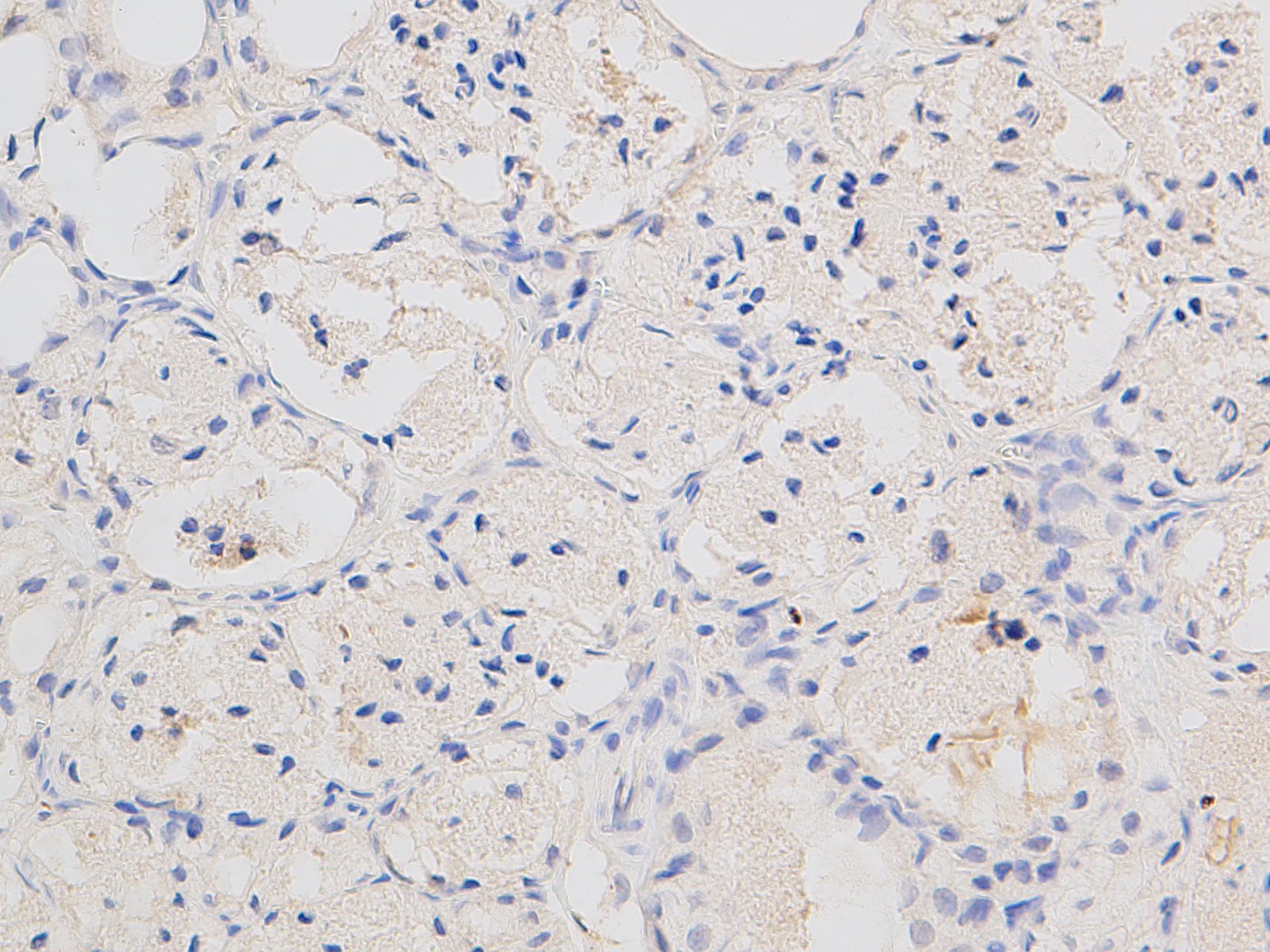

Supplement: Supplementary file 13 [file DataSheet13.zip › immunocytochemistry images/C3-400 (2).jpg]

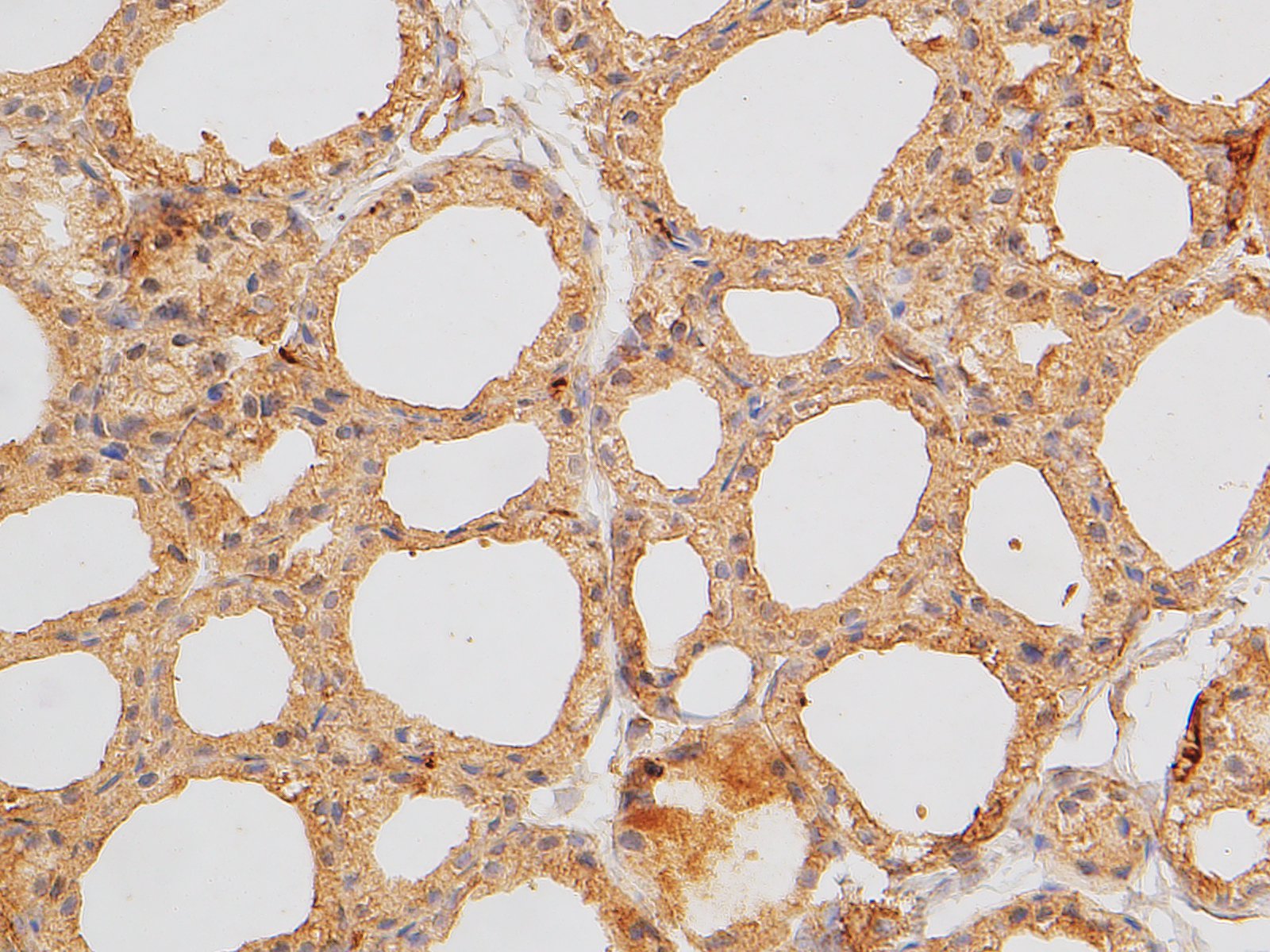

Supplement: Supplementary file 13 [file DataSheet13.zip › immunocytochemistry images/M1-400 (1).jpg]

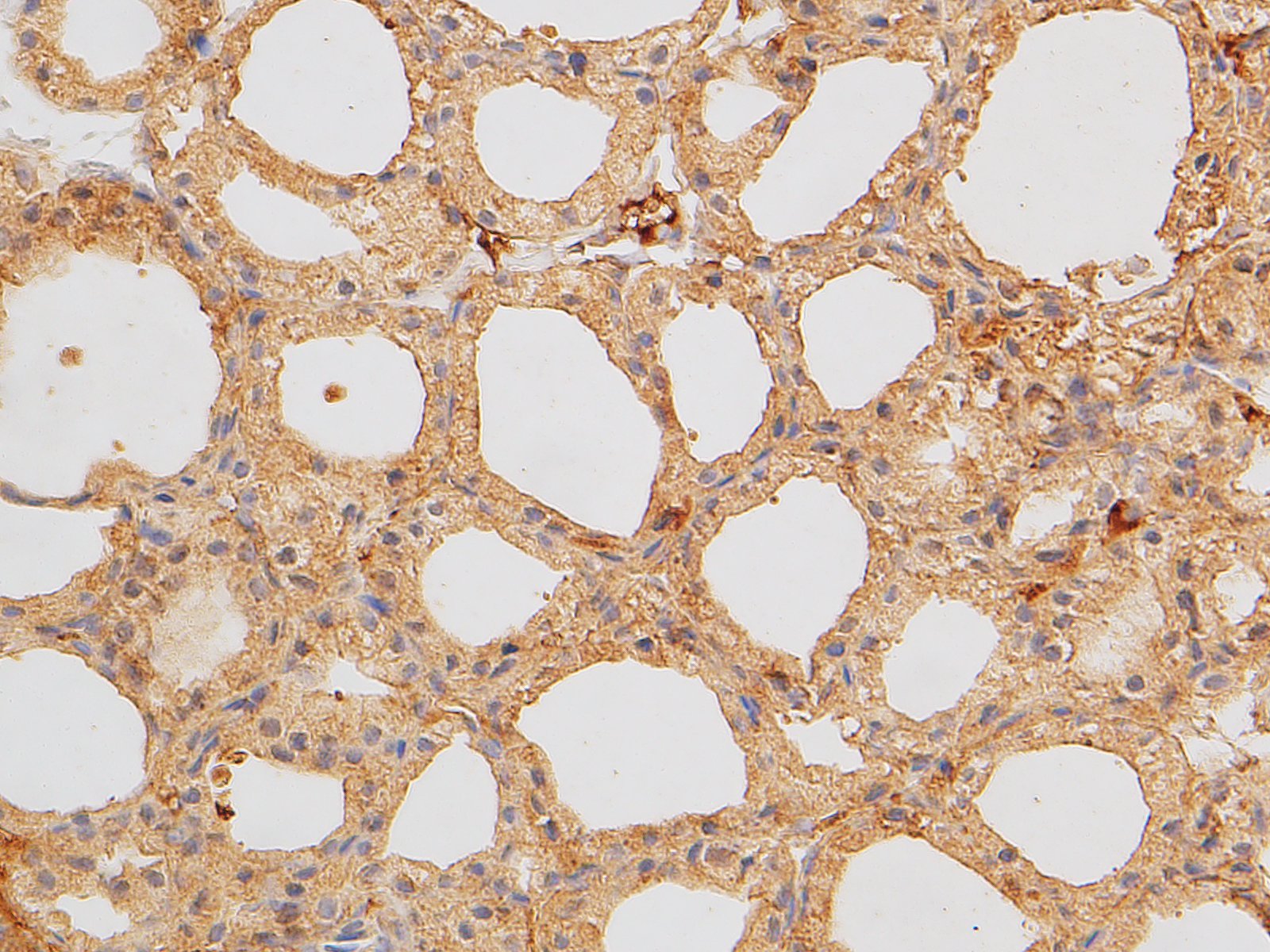

Supplement: Supplementary file 13 [file DataSheet13.zip › immunocytochemistry images/M1-400 (2).jpg]

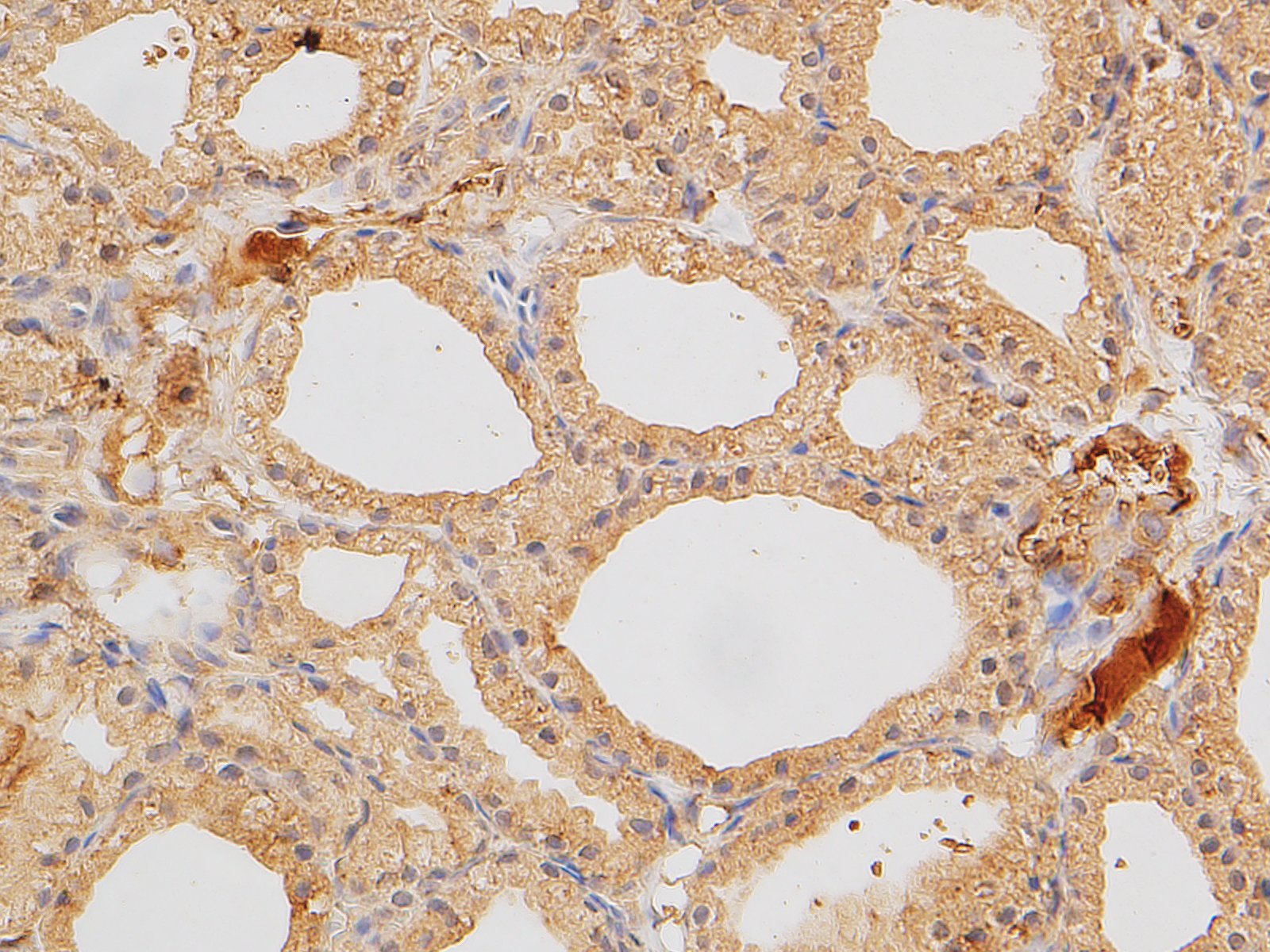

Supplement: Supplementary file 13 [file DataSheet13.zip › immunocytochemistry images/M2-400 (1).jpg]

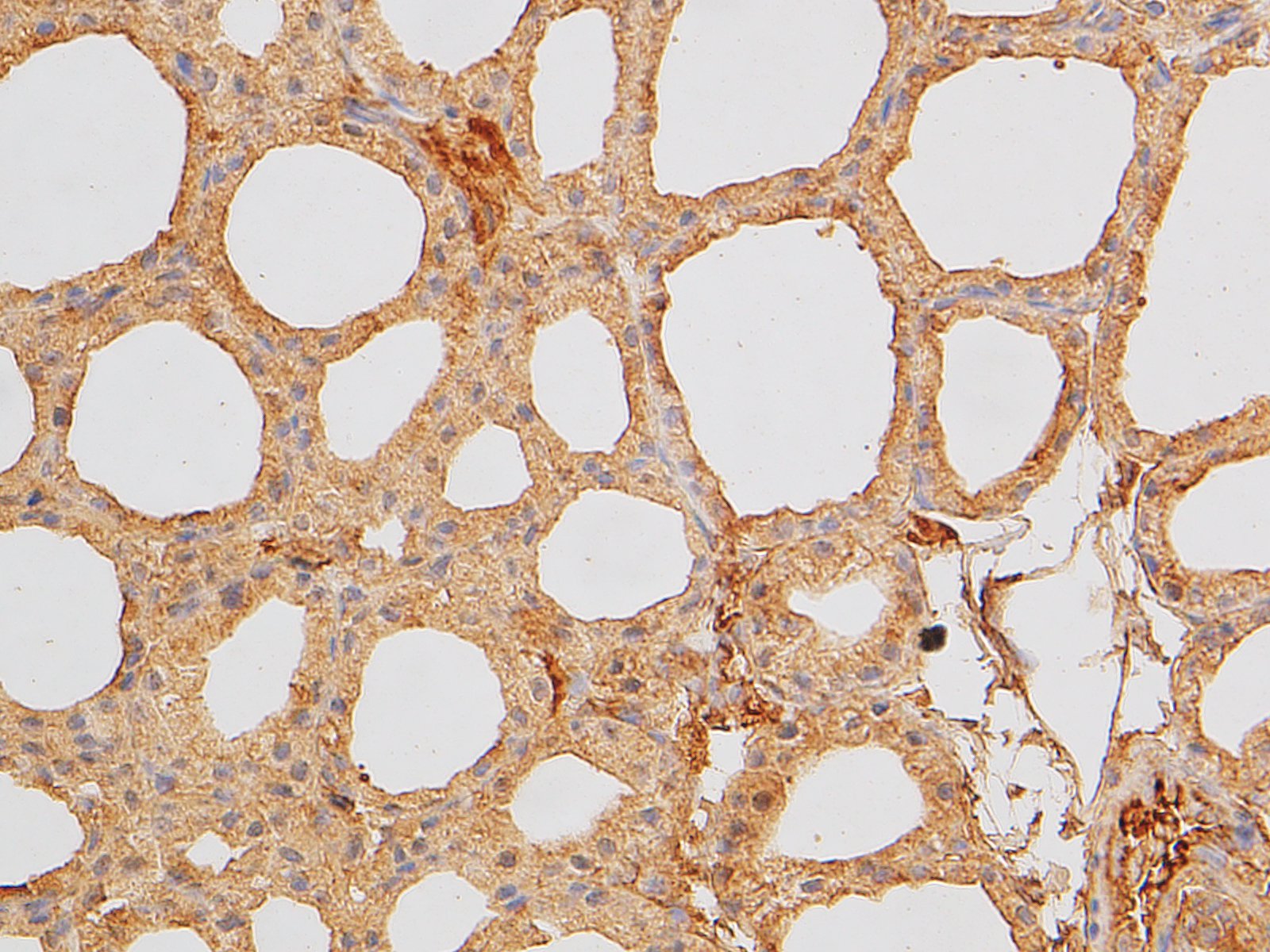

Supplement: Supplementary file 13 [file DataSheet13.zip › immunocytochemistry images/M2-400 (2).jpg]

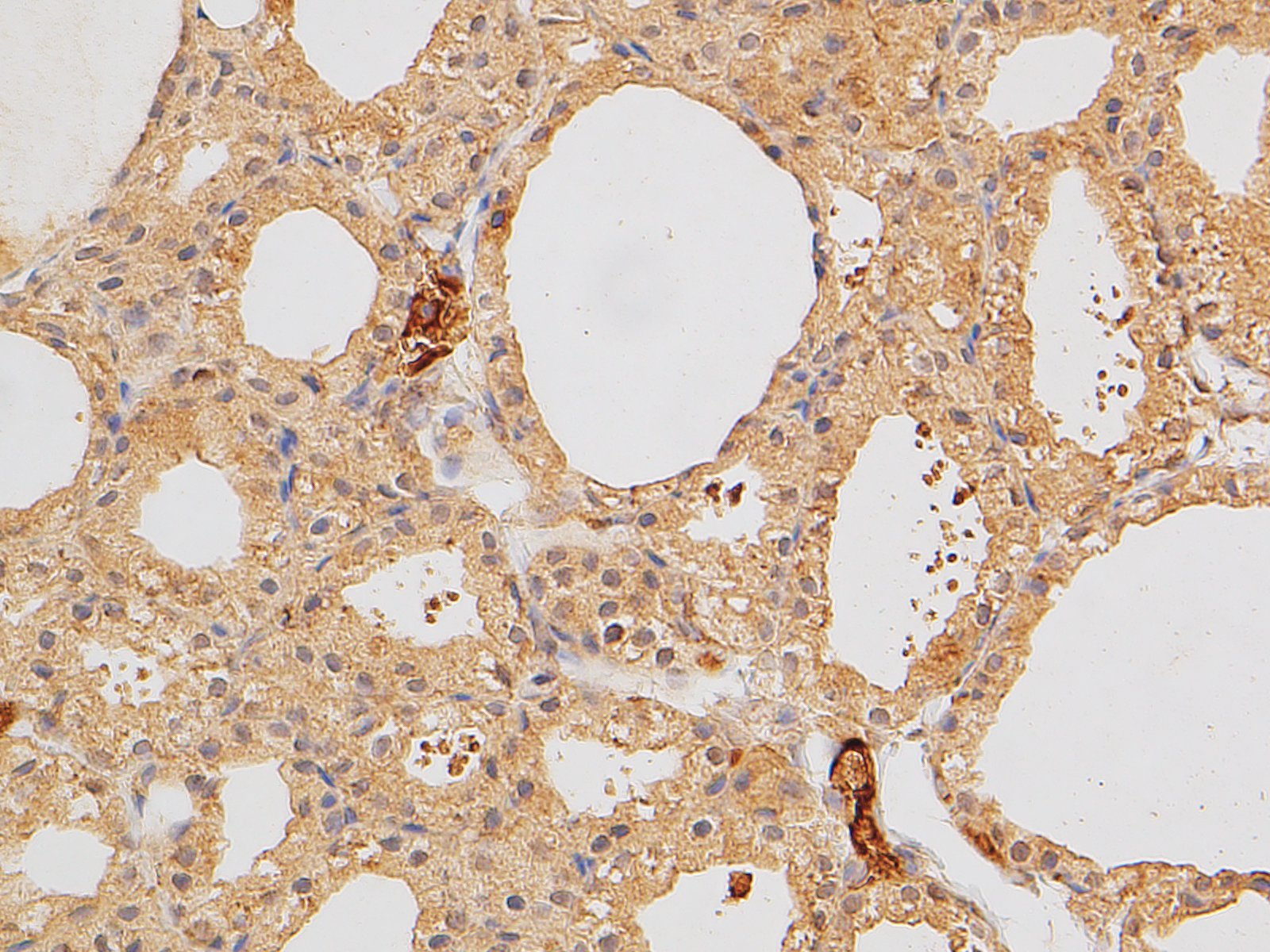

Supplement: Supplementary file 13 [file DataSheet13.zip › immunocytochemistry images/M3-400 (1).jpg]

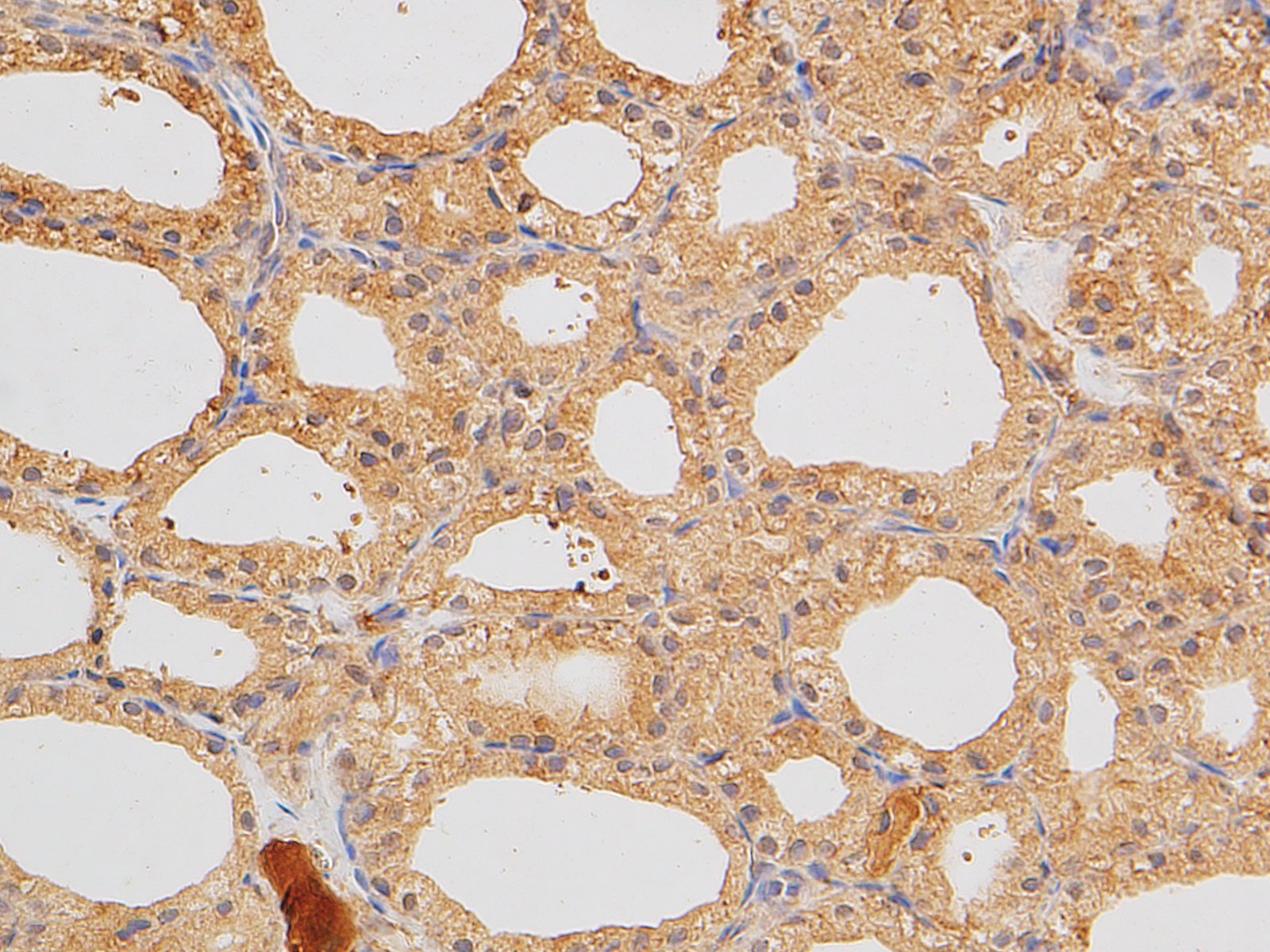

Supplement: Supplementary file 13 [file DataSheet13.zip › immunocytochemistry images/M3-400 (2).jpg]

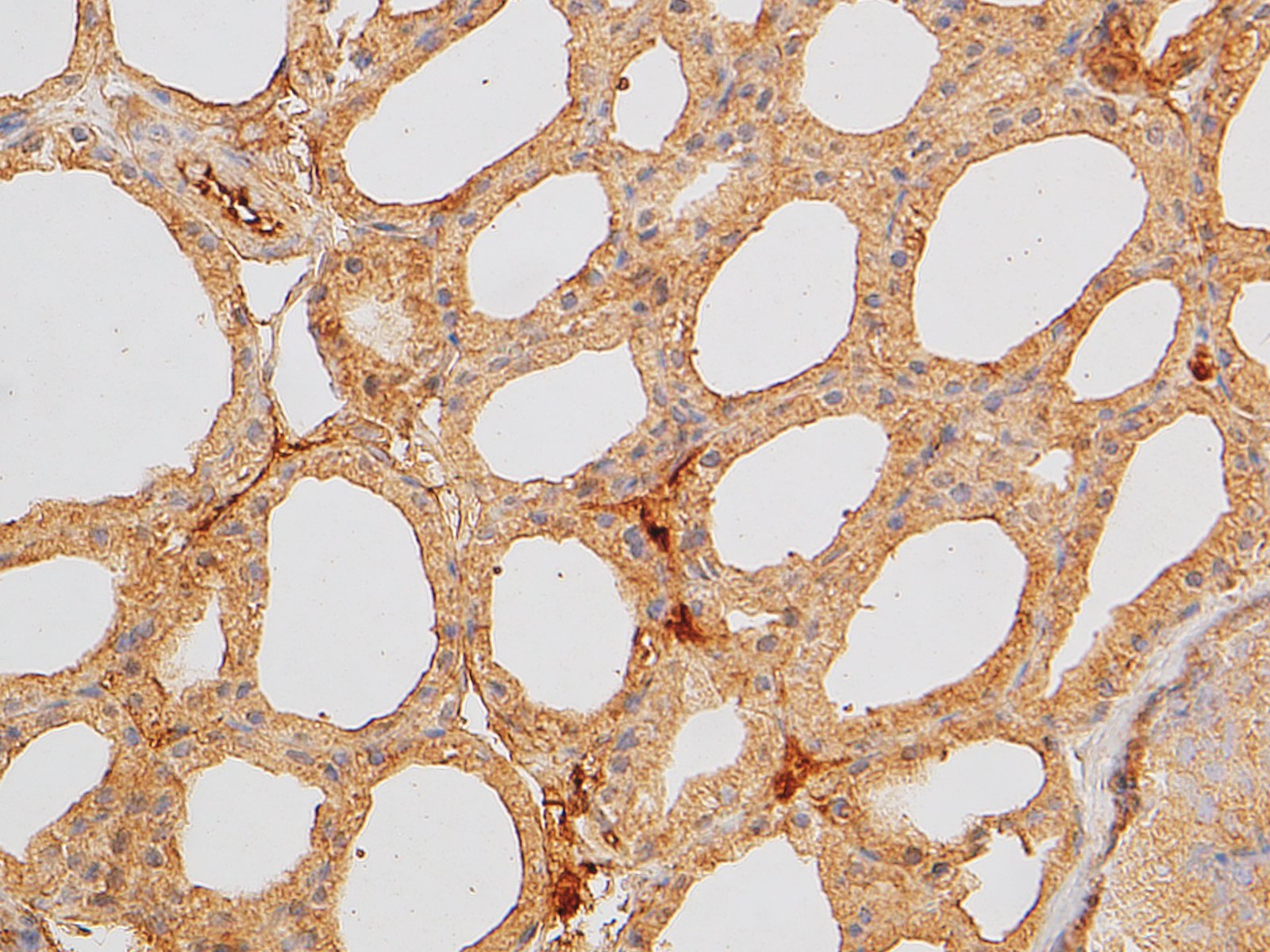

Supplement: Supplementary file 13 [file DataSheet13.zip › immunocytochemistry images/S1-400 (1).jpg]

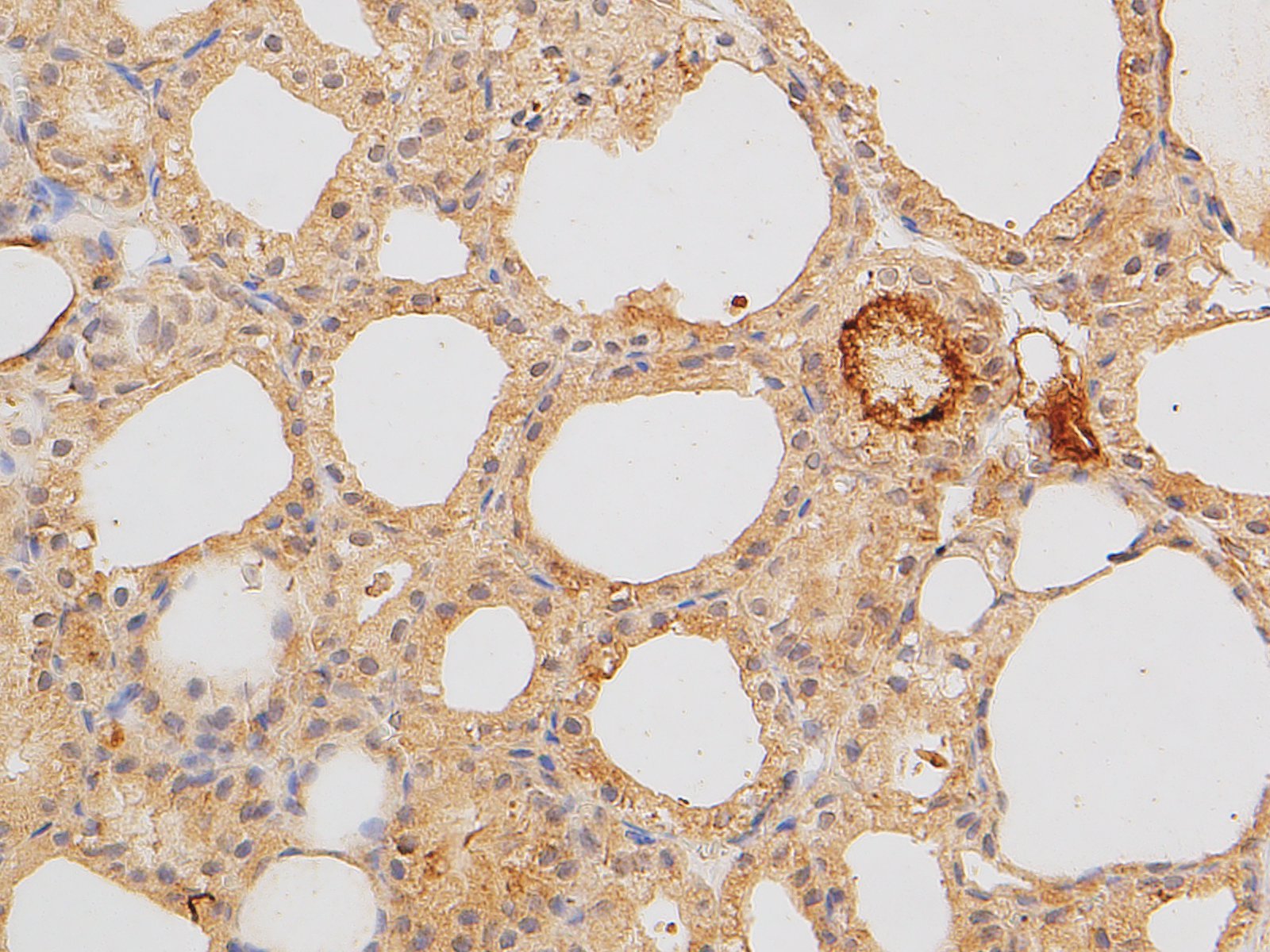

Supplement: Supplementary file 13 [file DataSheet13.zip › immunocytochemistry images/S1-400 (2).jpg]

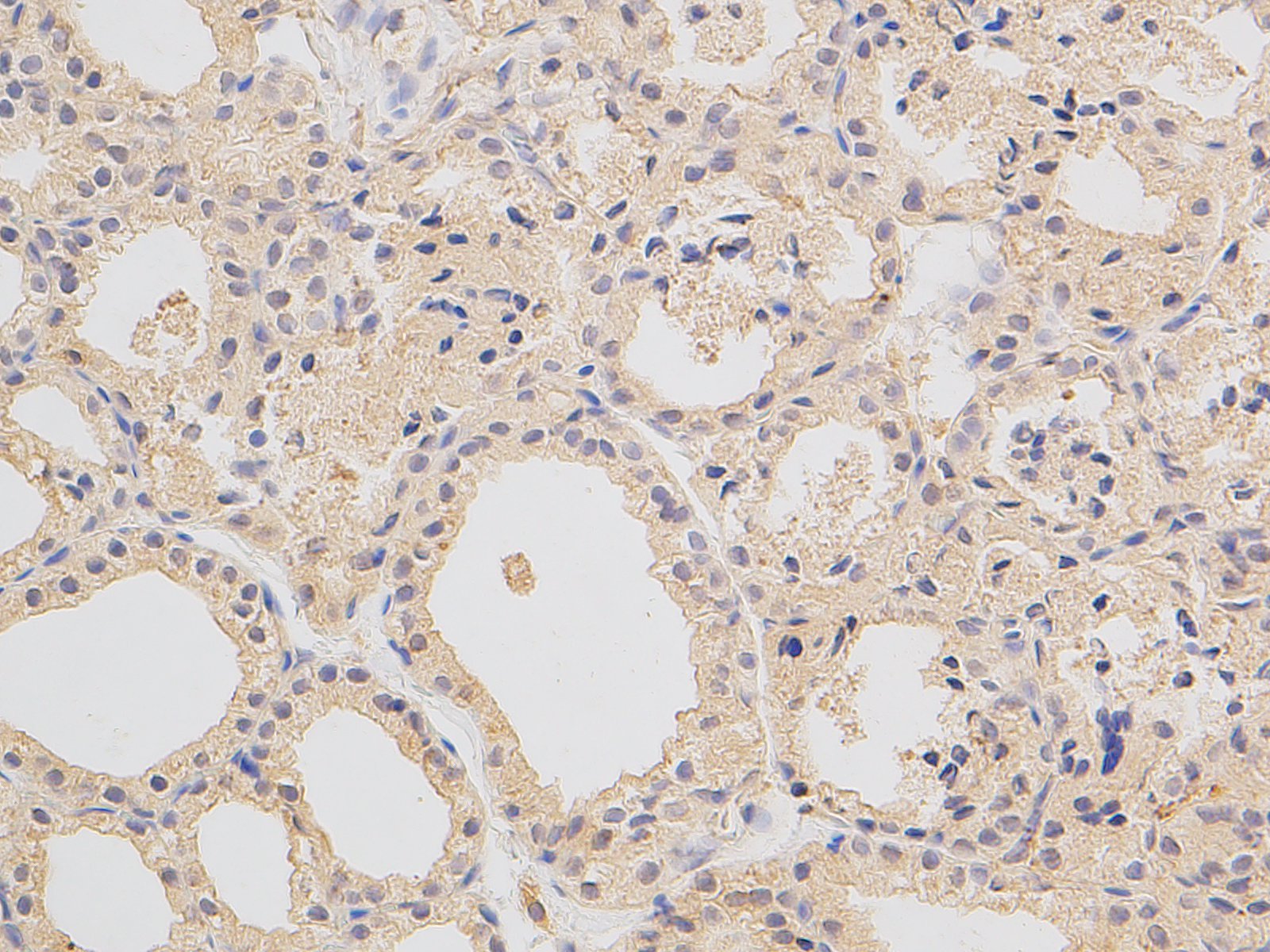

Supplement: Supplementary file 13 [file DataSheet13.zip › immunocytochemistry images/S2- 400 (1).jpg]

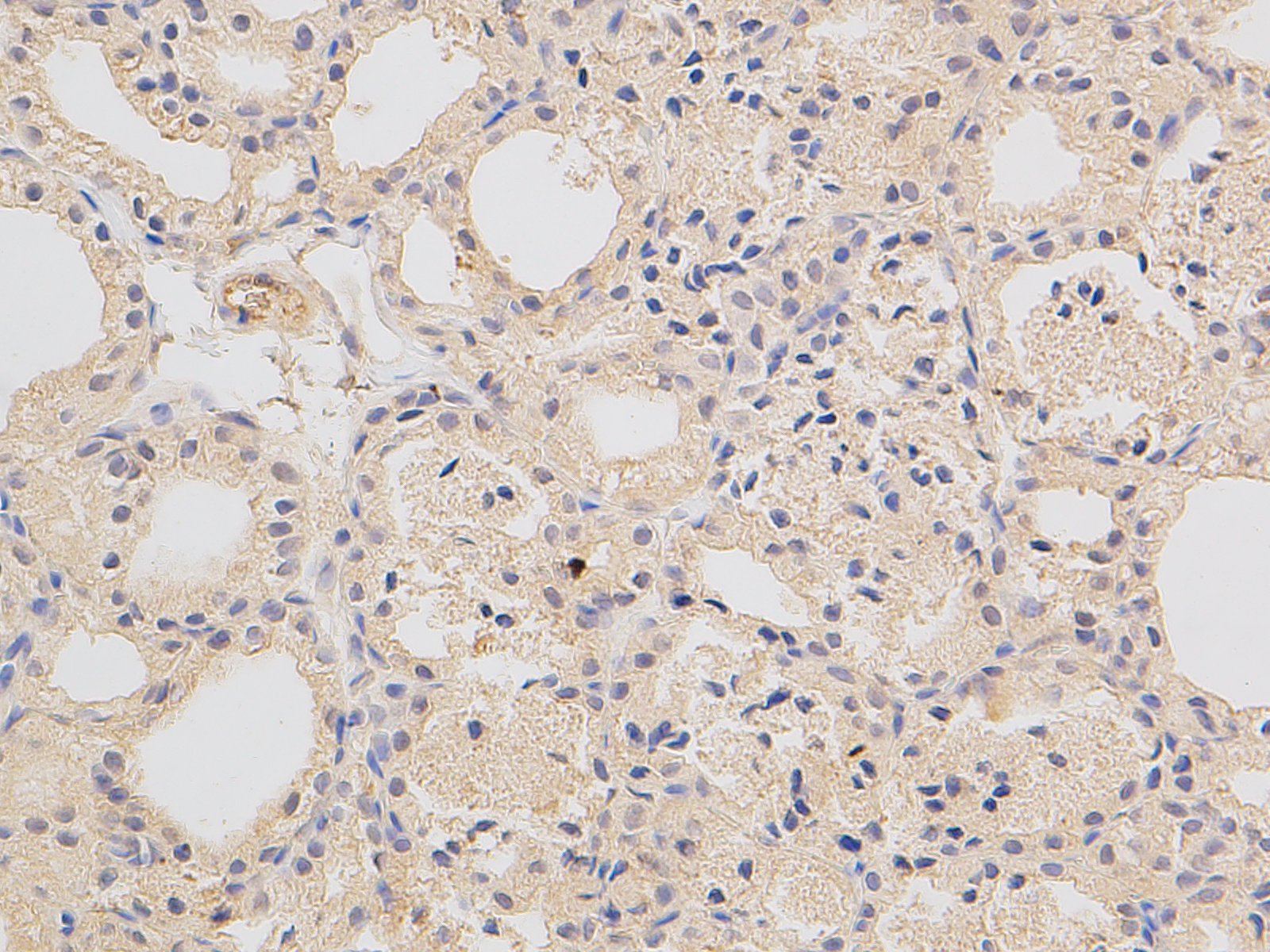

Supplement: Supplementary file 13 [file DataSheet13.zip › immunocytochemistry images/S2-400 (2).jpg]

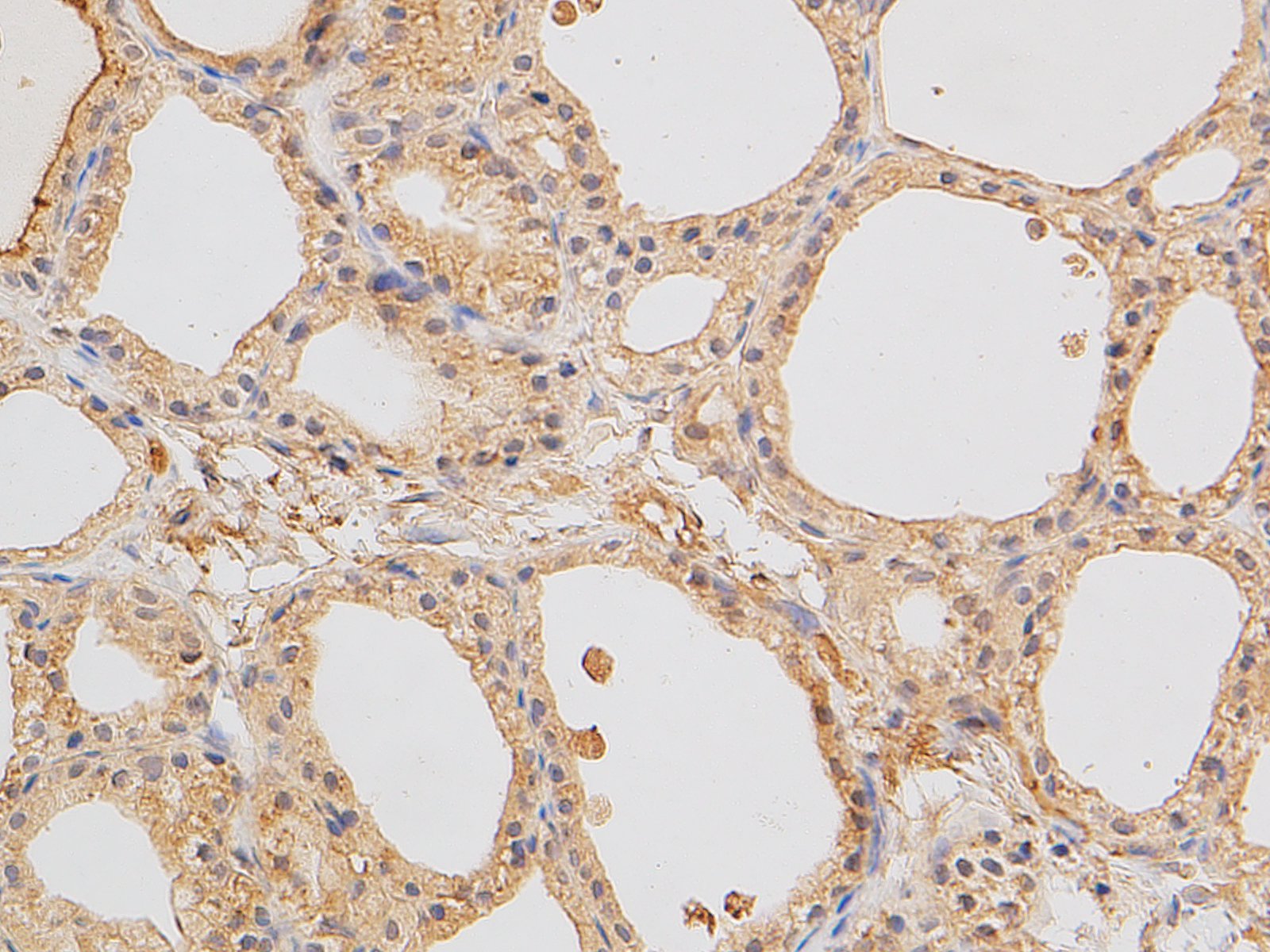

Supplement: Supplementary file 13 [file DataSheet13.zip › immunocytochemistry images/S3-400 (1).jpg]

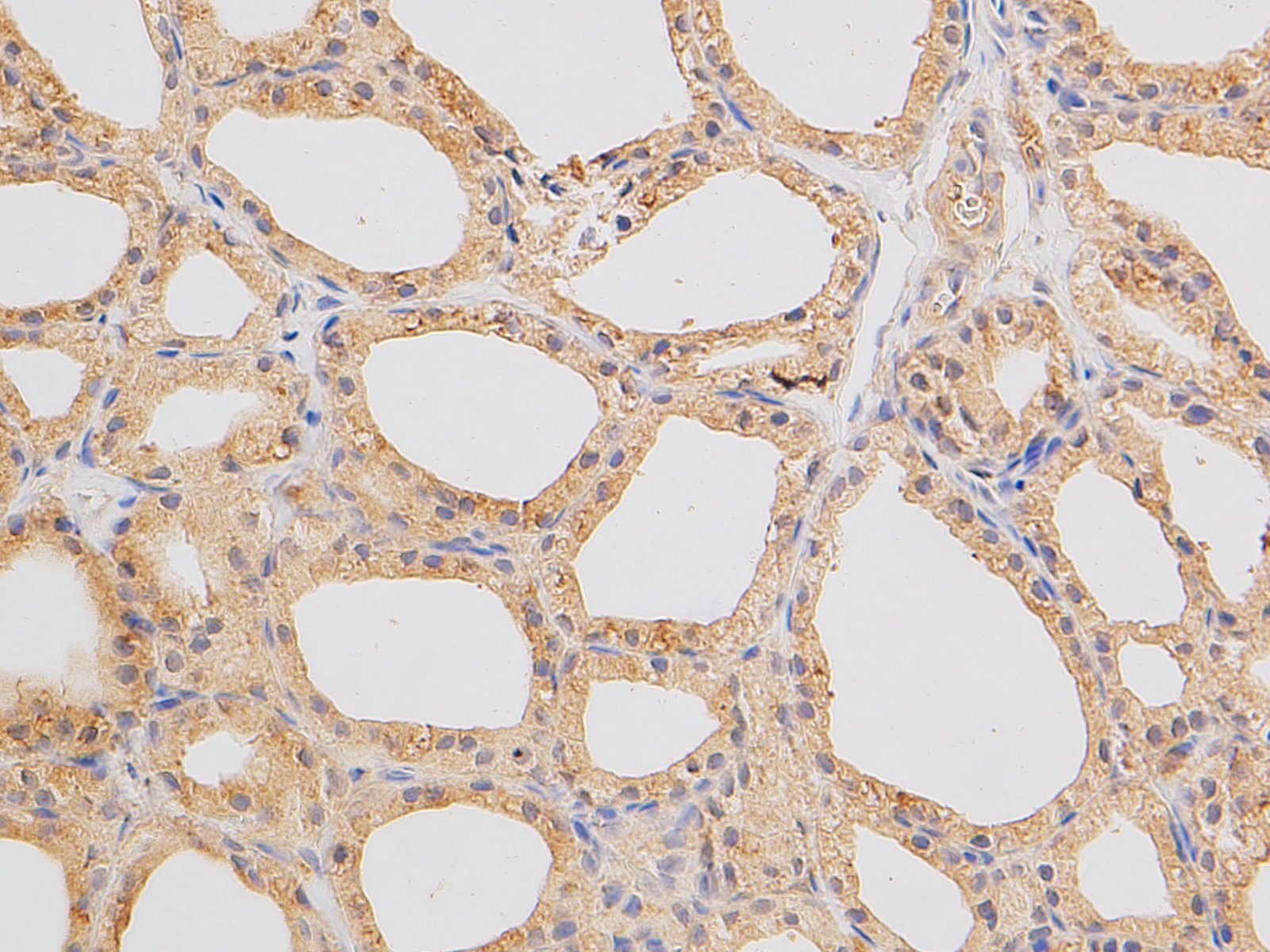

Supplement: Supplementary file 13 [file DataSheet13.zip › immunocytochemistry images/S3-400 (2).jpg]

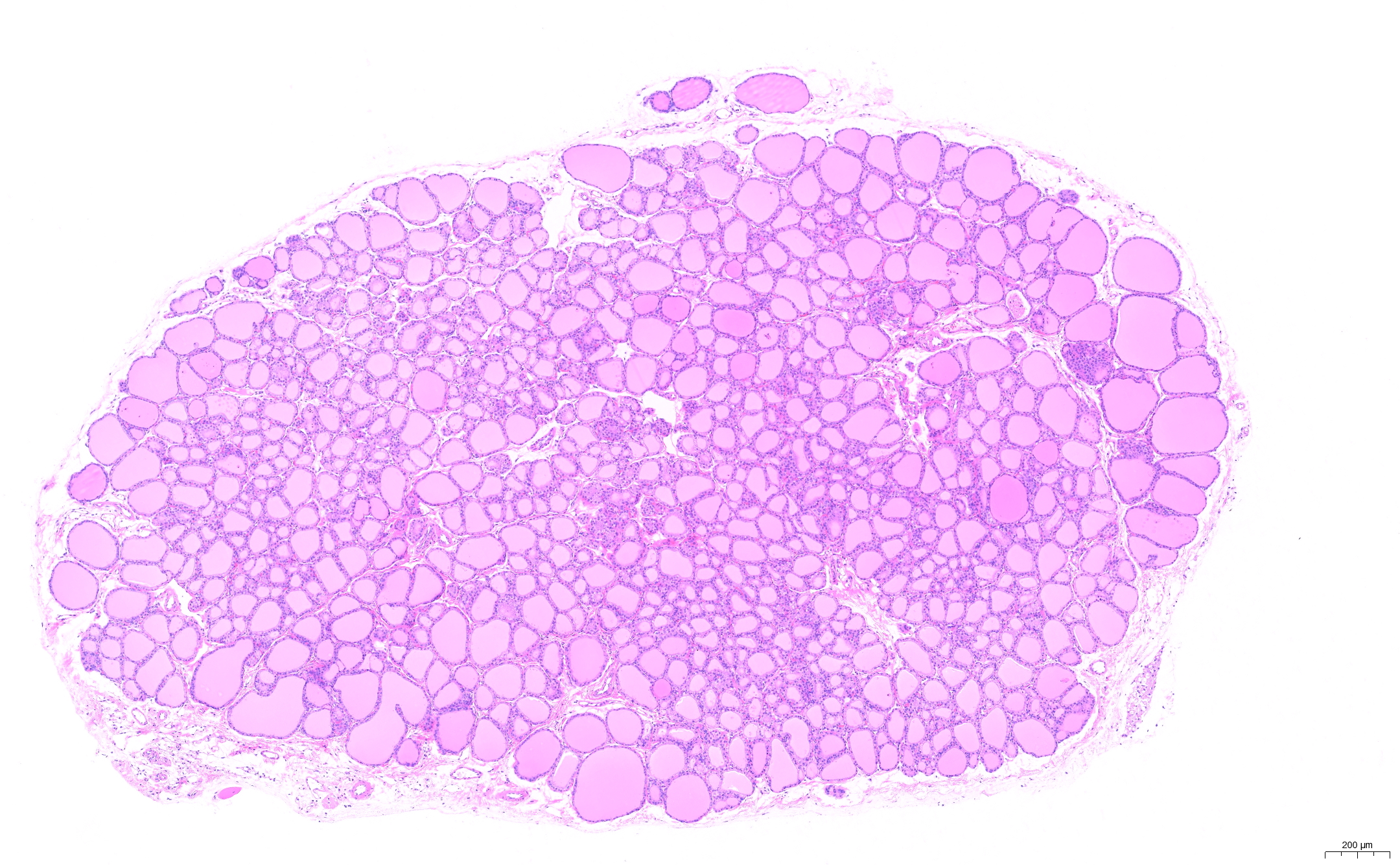

Supplement: Supplementary file 14 [file DataSheet14.zip › Hematoxylin-eosin staining images/HE(Acu).svs_5.0x.jpg]

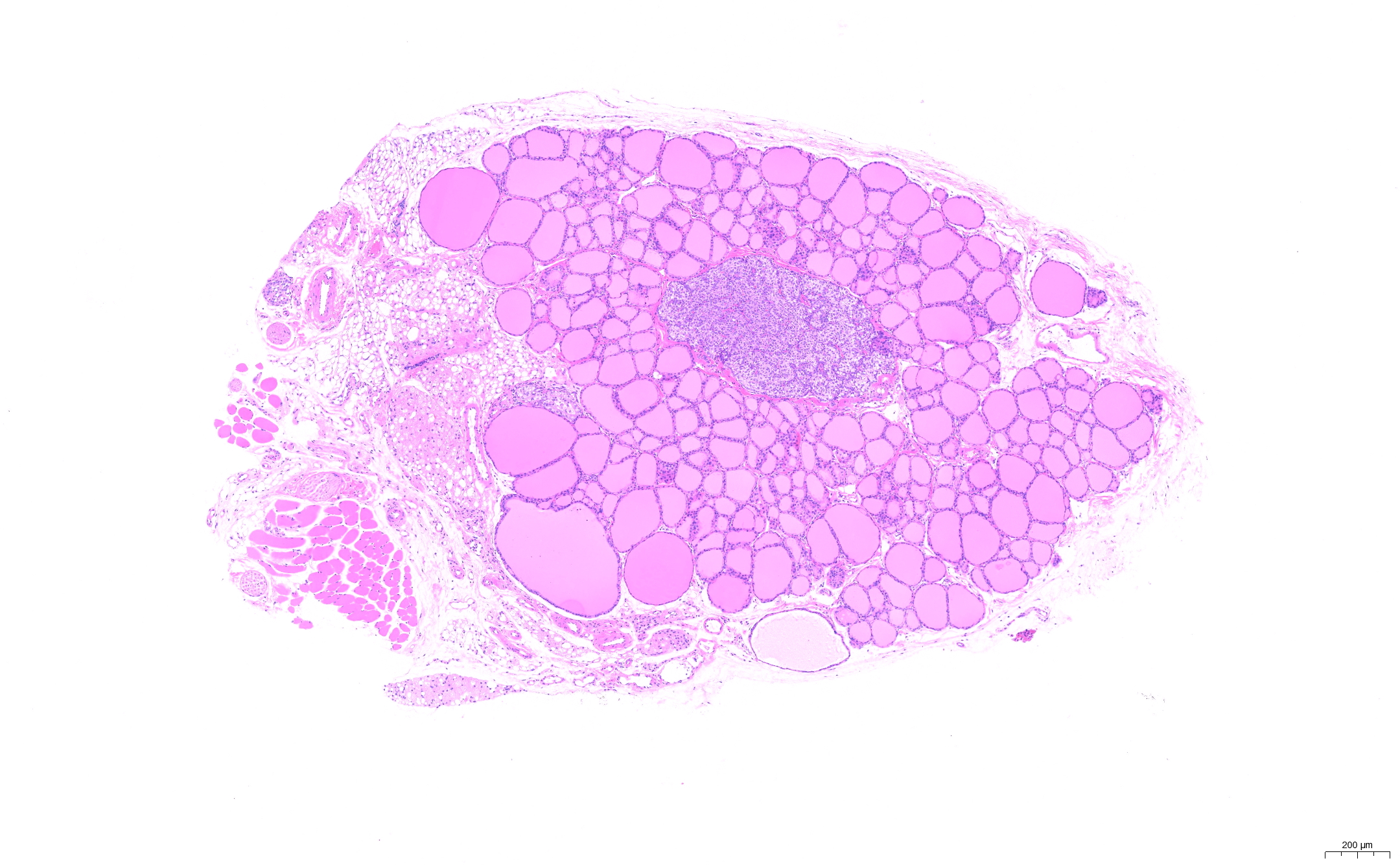

Supplement: Supplementary file 14 [file DataSheet14.zip › Hematoxylin-eosin staining images/HE(Control).svs_5.0x.jpg]

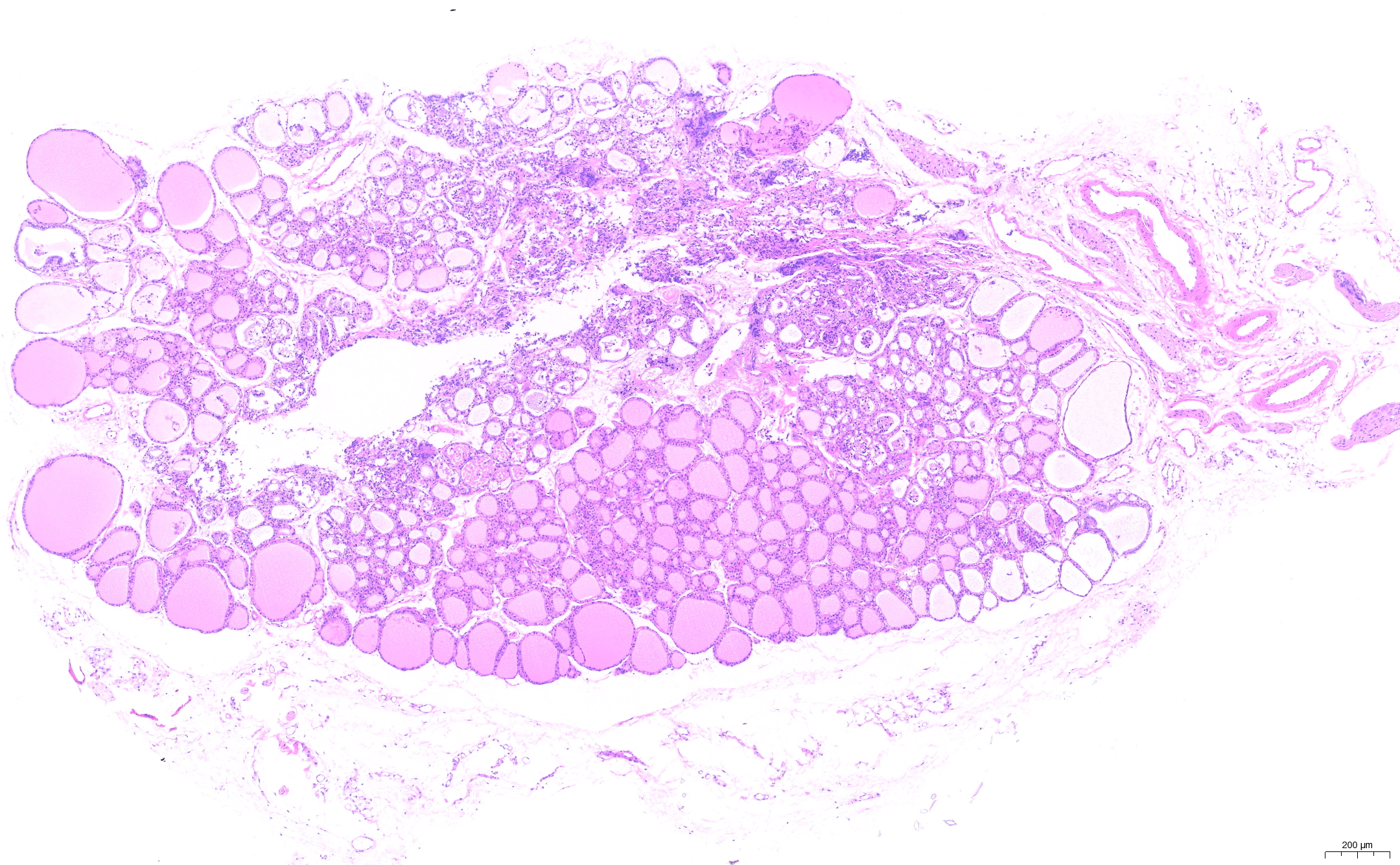

Supplement: Supplementary file 14 [file DataSheet14.zip › Hematoxylin-eosin staining images/HE(Model).svs_5.0x.jpg]

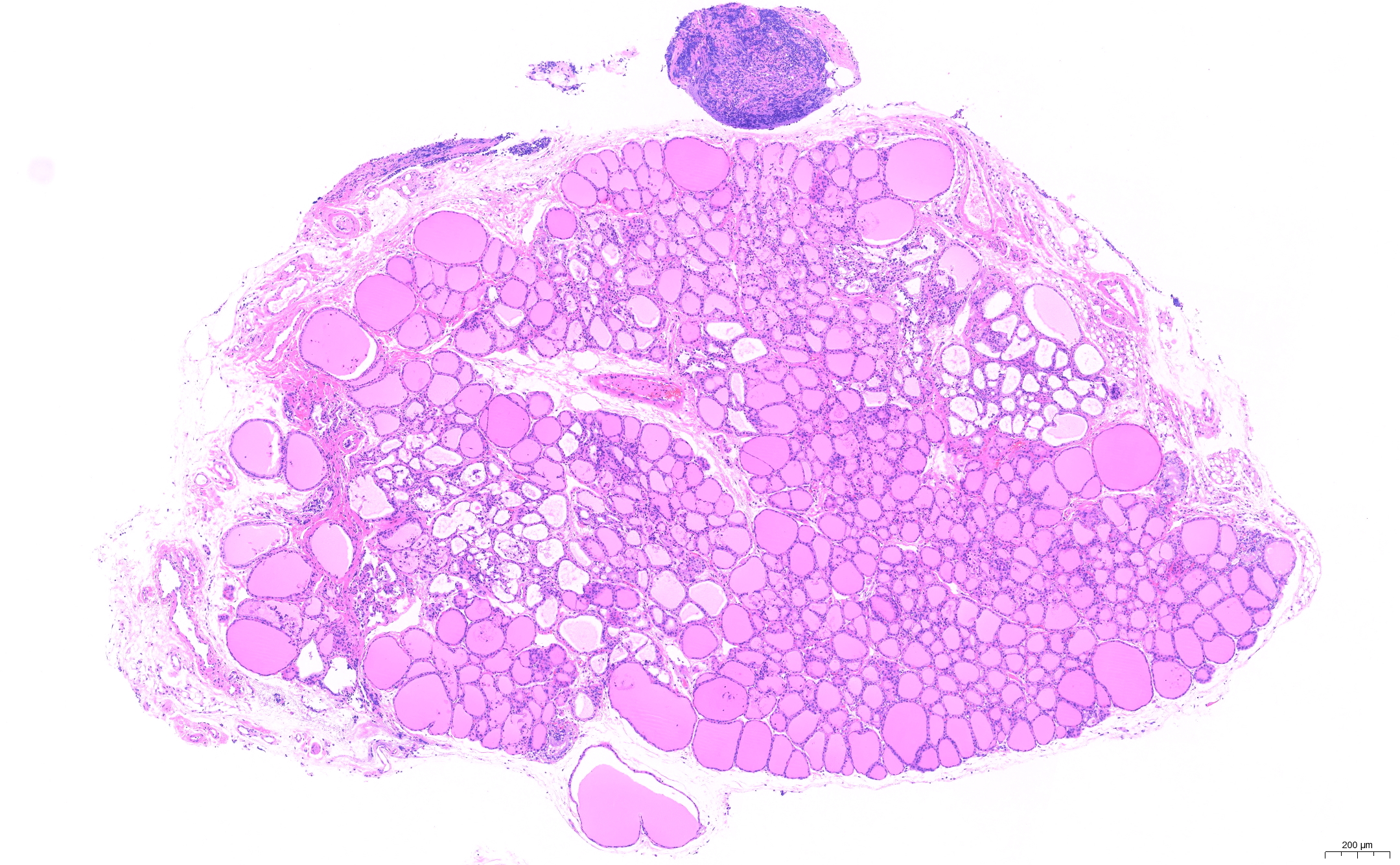

Supplement: Supplementary file 14 [file DataSheet14.zip › Hematoxylin-eosin staining images/HE(Se-yeast).svs_5.0x.jpg]
